# Pt(II) Rollover Complexes with Poor-Donor Bipyridines: Synthesis, Reactivity, and Preliminary Antimicrobial Evaluation

**DOI:** 10.1021/acsomega.6c05687

**Published:** 2026-07-30

**Authors:** Antonio Zucca, Matteo Murineddu, Fabrizio Ortu, Yu Liu, Gabriele Manca, Germano Orrù, Alessandra Scano, Maria I. Pilo

**Affiliations:** † 9312 University of Sassari, Department of Chemical, Physical, Mathematical and Natural Sciences, Via Vienna 2, Sassari I-07100, Italy; ‡ Consorzio Interuniversitario Reattività Chimica e Catalisi (CIRCC), Bari 70126, Italy; § School of Chemistry, 4488 University of Leicester, University Road, Leicester LE1 7RH, U.K.; ∥ Istituto di Chimica dei Composti Organometallici (ICCOM)-Sede Secondaria di Bari, Consiglio Nazionale delle Ricerche (CNR), 201843 c/o Università Degli Studi di Bari “Aldo Moro”, Via Orabona 4, Bari 70126, Italy; ⊥ Department of Surgical Sciences, 3111 University of Cagliari, Cagliari 09124, Italy

## Abstract

This study describes the synthesis, characterization, and preliminary antimicrobial investigation of a series of rollover platinum complexes derived from 6-chloro-2,2′-bipyridine (bpy^Cl^) and 6-bromo-2,2′-bipyridine (bpy^Br^) ligands. The electron-withdrawing halogen substituents in the 6-position significantly influence the coordination behavior of the ligands, favoring “rollover” cyclometalation rather than conventional N^N chelation. A series of platinum complexes, including [Pt­(bpy^X^-H)­(DMSO)­Me], [Pt­(bpy^X^-H)­(PPh_3_)­Me], [Pt­(bpy^X^-H)­(DMSO)­Cl], [Pt­(bpy^X^-H)­(PPh_3_)­Cl] (where X = Br or Cl), were successfully synthesized and characterized using multinuclear NMR spectroscopy and, in two cases, X-ray crystallography. The ligands’ stereoelectronic profile was evaluated based on the steric hindrance of the substituents and the electronic properties of the nitrogen donors. Electronic donor properties were correlated with NMR coupling constant data of the complexes. The reduced Lewis basicity of these ligands results in a noticeable reluctance to adopt the traditional N^N chelation motif; this is substantiated by the X-ray crystal structure of the complex [Pt­(bpy^Br^)­Cl_2_], which reveals a severe distortion induced by the bromine substituent. Conversely, a pronounced inclination toward rollover cyclometalation was observed. Selected complexes were thoroughly characterized and subsequently evaluated in a preliminary investigation of their antimicrobial activity against a panel of Gram-positive and Gram-negative multidrug-resistant bacteria, as well as a representative yeast strain, selected among clinically relevant and high-priority pathogens. Antibiofilm activity was also assessed, revealing moderate broad-spectrum antimicrobial effects and promising antibiofilm performance. These findings provide a useful basis for future structure–activity relationship studies and further optimization of the compounds. Furthermore, the reactivity of these species was also explored, leading to the isolation of various derivatives, including rare acetimine complexes obtained via room-temperature activation of ammonia and acetone.

## Graphical abstract

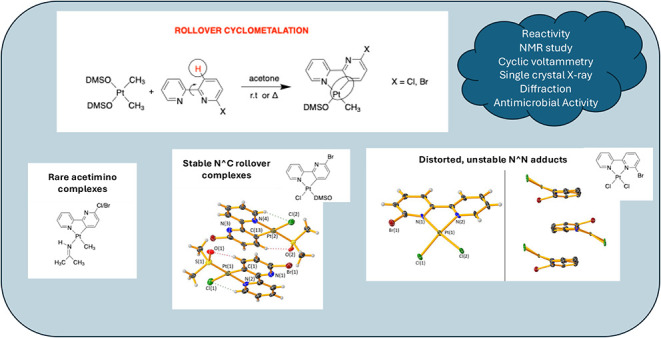

## Introduction

Cyclometalated complexes constitute an important class of organometallic compounds, characterized by exceptional stability and diverse applications in catalysis, biomedicine, and advanced materials science.^1–6^ These complexes feature a metal–carbon bond within a chelated ring, stabilized by the chelate effect. The field has evolved from classical structures derived from ligands such as 2-phenylpyridine to more sophisticated and less conventional species,^7^ such as pincer complexes,^8^ cyclometalated carbenes^9,10^ and “rollover” derivatives.^11–13^

Among these, pyridine-derived ligands exhibit remarkable coordination versatility. In particular, the combination of two six-membered aromatic rings, such as a phenyl or a pyridine, provides access to several useful coordination motifs (Figure 1), clearly differentiating 2,2’-bipyridines from related ligands such as substituted 1,10-phenanthrolines.^14–16^

**Figure 1:**
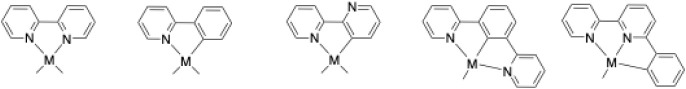
Coordination motifs of 2,2′-bipyridine and phenyl-pyridines.

These pyridine- or bipyridine-derived ligands can behave as neutral or anionic ligands, with additional features derived from the presence of other substituents.^17–22^ This versatility paves the way for numerous applications, including biomedical applications as anticancer agents^6,23–25^ and, more recently, as antimicrobial agents.^25–27^

This latter area has become of fundamental importance in recent years due to the rise of antimicrobial resistance (AMR) and multidrug resistance (MDR), which have emerged as critical threats to global public health. As conventional antibiotics progressively lose their efficacy, the development of novel antimicrobial agents has become not only urgent but essential. The World Health Organization (WHO) has classified AMR as a global health emergency, with profound implications across different contexts, such as human and animal healthcare, clinical treatments, veterinary, agriculture, food, and economy.^16,28–31^ Despite the growing demand for new antimicrobial agents, investment in their discovery remains insufficient. New compounds with innovative chemical structures and novel mechanisms of action are critically needed and coordination complexes based on noble metals have gathered increasing attention due to their unique physicochemical properties and mode of action.^27,32–35^ Notably, their multitarget mode of action, often involving mechanisms distinct from those exploited by conventional antibiotics, may reduce the likelihood of resistance development.

In the search for new compounds of potential interest, we focused on two electron-poor 2,2′-bipyridines, namely 6-chloro-2,2′-bipyridine (bpy^Cl^) and 6-bromo-2,2′-bipyridine (bpy^Br^), which exhibit significantly lower basicity than unsubstituted bipyridine-related systems. This reduced basicity is reflected in the p*K*_a_ values of the conjugate acids of the corresponding substituted pyridines (p*K*_a_(py) = 5.16; p*K*_a_(py^Cl^, 2-chloropyridine; py^Br^, 2-bromopyridine) = 2.84)^16^ and in their gas-phase proton affinity values (PA­(py) = 930.0 kJ mol^–1^; PA­(py^Br^) = 904.8 kJ mol^–1^; PA­(py^Cl^) = 900.9 kJ mol^–1^).^16^ These electronic features have important consequences for both the coordination chemistry and the potential applications of the resulting complexes.

The present work forms part of a broader systematic investigation into the synthesis, characterization, and properties of noble-metal complexes containing pyridine-derived ligands, with particular emphasis on the stereoelectronic effects of substituents and their influence on the antimicrobial activity of the resulting complexes.

## Results and Discussion

The 6-halogenated 2,2′-bipyridine ligands 6-chloro-2,2′-bipyridine (bpy^Cl^) and 6-bromo-2,2′-bipyridine (bpy^Br^) (Figure 2) possess two nitrogen donor atoms with significantly different electronic properties. One nitrogen atom exhibits characteristics similar to unsubstituted bipyridine, while the other displays substantially reduced Lewis basicity due to the electron-withdrawing effect of the halogen substituent. This electronic asymmetry is crucial for understanding the observed coordination behavior and reactivity patterns. The reduced basicity of these ligands is quantitatively demonstrated through thermodynamic data such as proton affinity^36,37^ and p*K*_a_ values^38^ of the corresponding chloro- and bromo- pyridines, indicating the increasing electron-withdrawing effect in the order H < Br < Cl (p*K*_a_ = 5.23 (H), 0.71 (Br), 0.49 (Cl); proton affinity = 930.0 (H), 904.8 (Br), 900.9 (Cl) kJ/mol). This electronic modulation directly influences the coordination chemistry and stability of the resulting metal complexes.

**Figure 2:**
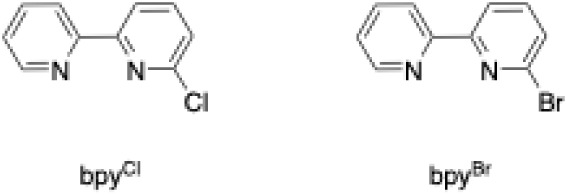
6-Chloro-2,2′-bipyridine (bpy^Cl^) and 6-bromo-2,2′-bipyridine (bpy^Br^).

The reaction of [Pt­(DMSO)_2_Me_2_] with 6-chloro-2,2′-bipyridine or 6-bromo-2,2′-bipyridine in acetone (Scheme 1) gave the cyclometalated complexes [Pt­(bpy^Cl^-H)­(DMSO)­Me], **1a**, and [Pt­(bpy^Br^-H)­(DMSO)­Me], **1b**, in high yield (*ca.* 90%). The rollover cyclometalation occurs readily, with the C^3^–H bond activation proceeding even at room temperature. However, to shorten the reaction time, complexes **1a** and **1b** can be prepared by heating at 60 °C for 1 h (see Scheme 1 for proton numbering). **1a** and **1b** were thoroughly characterized in solution by combining a suite of NMR studies, including ^1^H, ^13^C, 2D H–H COSY, H–H NOESY, H–C HSQC and H–C HMBC spectroscopies. The ^1^H NMR spectra of **1a** and **1b** exhibit the typical pattern of rollover-cyclometalated complexes: only six distinct signals appear in the aromatic region, consistent with the absence of the H^3^ proton; an AX system related to H^4^ and H^5^ protons is also consistent with rollover cyclometalation, observed at 7.93 and 7.16 ppm (**1a**) or 7.85 and 7.33 ppm (**1b**). ^195^Pt–^1^H satellites for H^4^ and H^5^ protons confirm metalation (J_Pt–H_ *ca* 83 and 16 Hz, for H^4^ and H^5^ respectively). In addition, the H^6′^ protons appear significantly deshielded at 9.70 ppm for **1a** and **1b**, and are also coupled to ^195^Pt. The coordinated DMSO and methyl ligands appear as singlets with satellites, e.g., in **1a**, 3.26 ppm (DMSO, J_Pt–H_ = 18.8 Hz) and 0.69 ppm (methyl, J_Pt–H_ = 81.8 Hz).

**Scheme 1:**
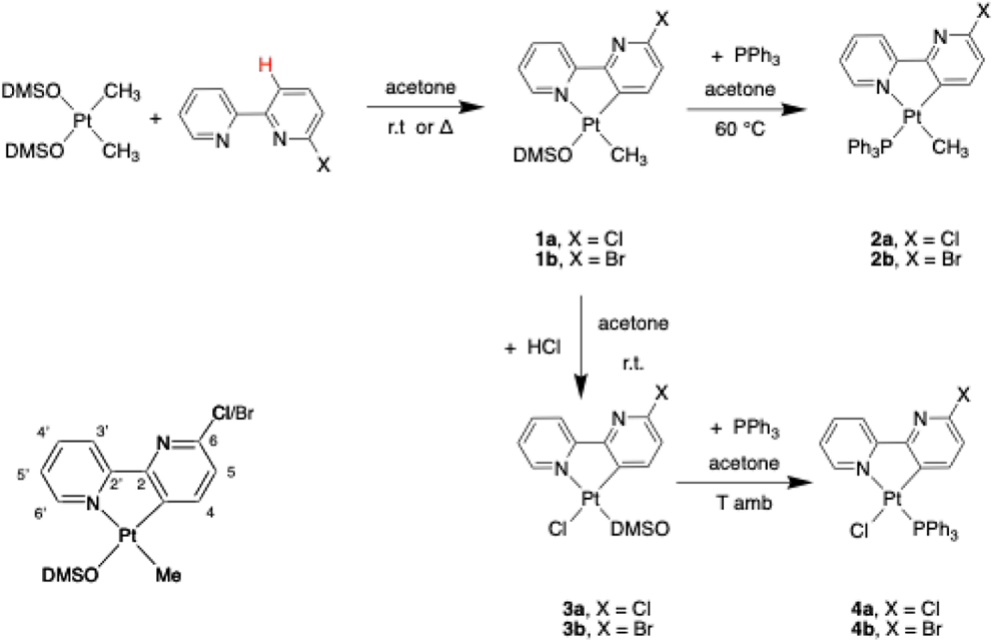
Synthesis of Rollover Complexes **1**-**4** ^a^

a On the lower left: numerical scheme for NMR discussion.

In the ^13^C NMR spectra the cyclometalated carbon C^3^ exhibits an exceptionally large direct coupling constant with ^159^Pt (**1a**, ^1^J_Pt–C_ = 1100 Hz; **1b**, ^1^J_Pt–C_ = 1104 Hz, Table 1), indicating the poor donor ability of the cyclometalated ligand. These values are consistent with the electron-withdrawing nature of the halogen substituent. As recently observed by some of us,^39^ cyclometalated donors with reduced donor properties display strong Pt–C direct couplings. This is illustrated by related cyclometalated complexes such as [Pt­(phpy-H)­(DMSO)­Me] (^1^J_Pt–C_ = 1063 Hz), [Pt­(bpy-H)­(DMSO)­Me] (^1^J_Pt–C_ = 1090 Hz), [Pt­(bpy^CF3^-H)­(DMSO)­Me] (^1^J_Pt–C_ = 1100 Hz), where phpy = 2-phenylpyridine, bpy = 2,2′-bipyridine, and bpy^CF3^ = 6-CF_3_-2,2′-bipyridine (Figure 3). The trend clearly follows the donor properties of the C-bonded pyridine ring, with higher values observed for electron-poorer donors. Furthermore, ^195^Pt–^13^C coupling constants for coordinated DMSO and methyl ligands confirmed the proposed structure.

**Figure 3:**
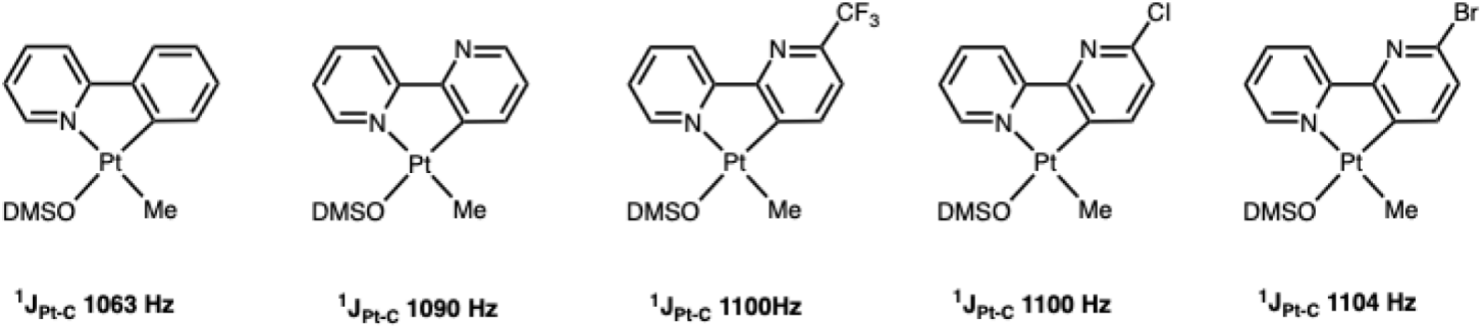
J_Pt–C_ values of comparable cyclometalated ligands.

**Table 1:**
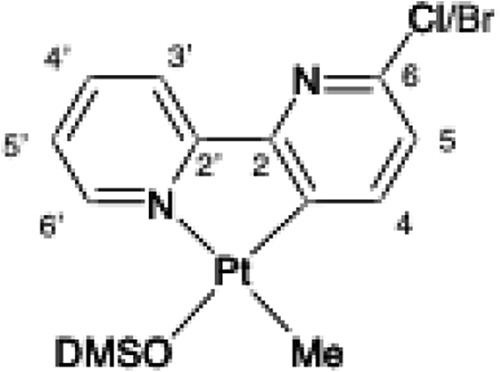
^13^C NMR Data for **1a** and **1b** (CDCl_3_, r.t.), along with the Numerical Scheme Used^a^

Carbon	[Pt(bpy^Cl^-H)(DMSO)Me] 1a	[Pt(bpy^Br^-H)(DMSO)Me] 1b
C2	165.06 (30.1)	165.68 (n.r.)
C3	143.48 (1098)	144.00 (1107)
C4	143.08	142.86 (94.4)
C5	124.10	127.81 (63.8)
C6	147.87	143.98
C2′	161.14	161.11
C3′	121.58 (23.4 Hz)	121.36 (24.2)
C4′	138.64	138.64
C5′	124.97 (9.7)	124.99
C6′	150.42	150.41
Me	–13.70 (758.7)	–13.71 (769)
DMSO	43.80 (43.8)	43.80 (45.2)

a ^1^J_Pt–H_, in Hertz, in parentheses; n.r.= not resolved.

Comprehensive 2D H–H NOESY and H–C HMBC experiments for **1a** and **1b** complete the characterization in solution: the NOESY spectra show, inter alia, the expected NOE contacts between the coordinated methyl and H^4^ and DMSO protons (Figure 4). The H–C HMBC spectra show for both **1a** and **1b** the expected correlations; as an example, long-range correlations between the coordinated methyl protons and C^3^, C^4^ and DMSO carbon atoms.

**Figure 4:**
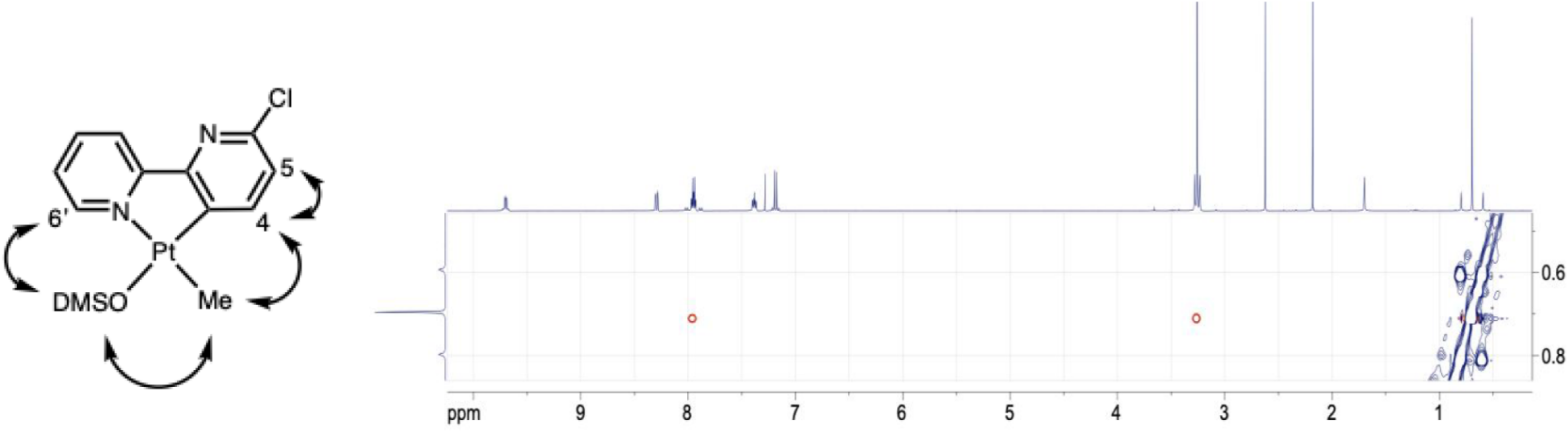
Left: NOE contacts in complex **1a**, as showed by the 2D NOESY NMR spectrum; Right: expansion of NOESY spectrum of **1a** showing NOESY contacts of the coordinated methyl.

## Rollover Reaction Insights

### Electronic and Structural Implications

One of the most intriguing aspects of rollover cyclometalation is the identification of the factors that promote it. Starting from typically stable chelating complexes, it is of paramount importance to elucidate the driving forces that underpin this process; in this regard, rollover cyclometalation differs significantly from classical cyclometalation. Furthermore, since each metal operates through distinct mechanistic pathways, conditions that favor the reaction for one metal may not apply to others. For instance, in gold­(III) complexes, the determining factors remain poorly understood and such examples are extremely rare,^40^ although the resulting complexes have exhibited promising antitumor properties.^41^ In the case of platinum, it has been demonstrated from the pioneering work by Young and co-workers^42^ that the metal displays nucleophilic behavior during the process; therefore, a highly electron-rich metal center is crucial.

In a previous study^43^ we analyzed the influence of steric and electronic factors in 6-substituted 2,2′-bipyridines. The steric hindrance in these ligands, arising from the substituent positioned adjacent to a nitrogen atom, facilitates the detachment of one pyridine ring, thereby allowing rotation around the inter-ring C–C bond. On the other hand, the electron-withdrawing ability of substituents such as CF_3_, Cl, and Br exerts a dual effect: first, it reduces the electron-donating capacity of the nitrogen atom, thus synergizing with the steric factor to promote nitrogen dissociation; second, it influences the C(3)–H bond located in the *para* position. In their pioneering 1985 work,^42^ Young and co-workers demonstrated through kinetic studies the nucleophilic character of the platinum center during the C–H activation process, which ultimately leads to the formation of the rollover cyclometalated complex.

For platinum­(II), the factors favoring rollover cyclometalation include a bulky and electron-withdrawing substituent that decreases the donor capacity of the coordinating nitrogen, favors N-detachment and renders the *para* C–H bond more susceptible to nucleophilic attack.

To quantify steric hindrance in square planar chelated complexes, we introduced the angle ζ, defined as the angle between a line which bisects the N–Pt–N angle, and a line tangent to the outermost van der Waals surface of the substituent, starting from the metal center, as depicted in Table 2 for a model complex [Pt­(N^N)­(CH_3_)_2_].^43^

**Table 2:**
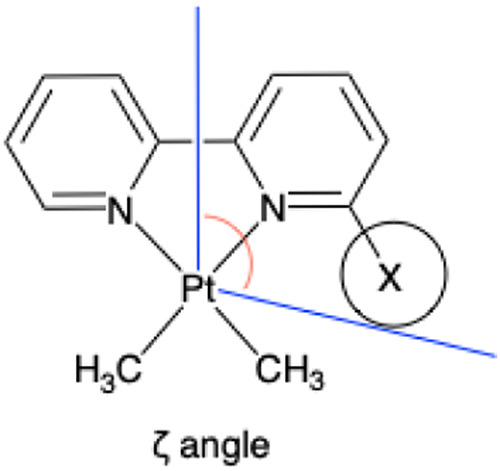
Steric Requirements (ζ Angle) for 6-Substituted 2,2′-Bipyridines^a^, Acid Dissociation Constants, Proton Affinity and Gas-Phase Basicity Values for 2-Substituted Pyridines^b^, as a Function of the Substituent, and on the Right: Depiction of the ζ angle^c^

	Substituent	ζ (deg)	p*K*_a_ ^36,37^ py^R^	Proton affinity^38^ (kJ/mol)	Gas-phase basicity^38^ (kJ/mol)
bpy/py	H	98.8	5.23	930.0^d^	898.1
bpy^F^/py^F^	F	107.3	–0.44	884.6	852.7
bpy^CH_3_^/py^CH_3_^	CH_3_	125.1	6.00	949.1	917.3
bpy^CF_3_^/py^CF_3_^	CF_3_	137.6	–	887.1	855.2
bpy^Cl^/py^Cl^	Cl	117.4	0.49	900.9	869.0
bpy^Br^/py^Br^	Br	121.2	0.71	904.8	873.0

a bpy^F^ = 6-fluoro-2,2′-bipyridine; bpy^CH3^ = 6-methyl-2,2′-bipyridine; bpy^CF3^ = 6-trifluoromethyl-2,2′-bipyridine.

b p*K*_a_ values in water for corresponding 2-substituted pyridinium ions.

c Values used for the calculation: Cl: covalent radius = 102 pm, vdW radius = 175 pm; Br, covalent radius = 120 pm, vdW radius = 187 pm.

d For comparison, the proton affinity value of unsubstituted bpy is 933.4 kJ/mol.

This electronic effect can be quantified using nitrogen gas-phase proton affinity and basicity^36,37^ of the corresponding substituted pyridines, and p*K*_a_ values in solution of the conjugated acids, i.e., the corresponding pyridinium ions.^38^

From a steric perspective, the presence of a bulky substituent near the nitrogen is highly favorable, also in this case, to promote N-detachment.

A notable example is the CF_3_ group, which exhibits a ζ angle of 137.6° and a proton affinity of 887.1 kJ/mol (Table 2) to be compared to simply 2,2′-bipyridine (98.9° and 930 kJ/mol, respectively). Both steric and electronic parameters in bpy^CF3^ strongly favor rollover metalation, which occurs rapidly at room temperature, on contrast to unsubstituted bipyridine which requires refluxing toluene.

Using our methodology[Fn ba-fn1] with bond distances derived from the covalent radii published by Cordero et al.^44^ and van der Waals radii from Bondi,^45^ we calculated a ζ angle of 117.4° for chlorine and 121.2° for bromine. These ligands exert significantly greater steric hindrance than fluorine (107.3°) and hydrogen (98.8°) due to their larger van der Waals radii and longer bond lengths, which push the atomic perimeter further outward. Nevertheless, they remain less sterically demanding than a methyl group (125.1°).

As for the donor properties of the nitrogen, p*K*_a_ values for corresponding 2-substituted pyridines have been reported, showing lower basicity for a chlorine in 2-position (p*K*_a_ 0.49) with respect to bromine (p*K*_a_ 0.71).^36,37^ Also in the gas-phase proton affinity and gas-phase basicity data show the same trend.^38^

All these data, taken together, account for a greater bulkiness for bpy^Br^ but enhanced electronic influence for the bpy^Cl^ ligand.

In addition to ligands’ properties, the large ^195^Pt–C and ^195^Pt–P coupling constants observed in the rollover complexes of bpy^Cl^ and bpy^Br^ (*e.g.*, ^1^J_Pt–C_ = 1100 Hz in **1a**, 1107 in **2a**; ^1^J_Pt–P_ = 2295 Hz in **2a** and **2b**) provide quantitative evidence for the reduced Lewis basicity of these ligands, which affects not only the easiness of the rollover process, but also the properties of the resulting complexes and, likely, the state of the platinum center.

### Mechanistic Investigation

A comprehensive Density Functional Theory (DFT) investigation was carried out to elucidate the key mechanistic aspects of the reactivity of the [Pt­(DMSO)_2_Me_2_] complex (denoted as **A** in the calculations) toward different bipyridine ligands. The two bipyridine derivatives considered were 6-chloro-2,2′-bipyridine and 6-bromo-2,2′-bipyridine, bearing Cl and Br substituents, respectively (indicated as bpy^Cl^ and bpy^Br^). The calculations were performed at the M06–2X level of theory,^46^ including dispersion corrections.^47^ Solvent effects were taken into account using the Conductor-like Polarizable Continuum Model (CPCM)^48,49^ with acetone, the same solvent employed in the experimental studies (see the Computational Details section for further information). The mechanistic analysis started from intermediates **B1** and **C1**, in which the platinum center is coordinated by the two nitrogen atoms of the bipyridine ligand and two methyl groups. The optimized structures of these intermediates are shown in Figure 5. The formation of **B1** and **C1** from **A** and 6-chloro-2,2′-bipyridine or 6-bromo-2,2′-bipyridine, respectively, was calculated to be exergonic by −8.6 and −7.5 kcal mol^–1^, respectively.

**Figure 5:**
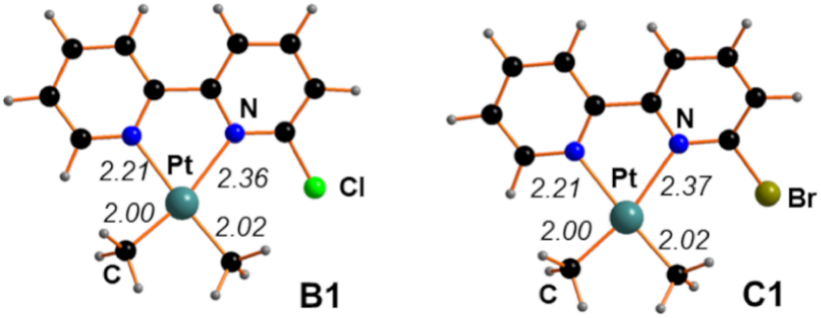
Optimized structures of square-planar platinum complexes **B1** and **C1** with two methyl and one bipyridinic chelate ligands. Selected bond lengths (in Å) are reported *in italics*.

The presence of a halogen atom at the 6-position of the pyridine ring induces a pronounced asymmetry in the Pt–N bond distances in intermediates **B1** and **C1**. Specifically, the Pt–N bond involving the substituted pyridine ring is more elongated than the corresponding bond to the unsubstituted ring by more than 0.15 Å. The final products feature coordination of the bipyridine ligand through one nitrogen atom and one carbon atom, indicating that initial C–H bond activation has occurred. The formation of this coordination mode requires a rollover of the bipyridine ligand about the inter-ring C–C bond, which is accompanied by the initial cleavage of one Pt–N bond. DFT calculations identified the corresponding transition states, **BTS1–2** and **CTS1–2**, shown in Figure 6, with computed free-energy barriers of 12.4 and 11.2 kcal mol^–1^, respectively. The same rollover process was also investigated for the platinum complex bearing unsubstituted 2,2′-bipyridine. In this case, the calculated free-energy barrier exceeds 23 kcal mol^–1^, indicating that the process is not feasible under the experimental conditions at room temperature.

**Figure 6:**
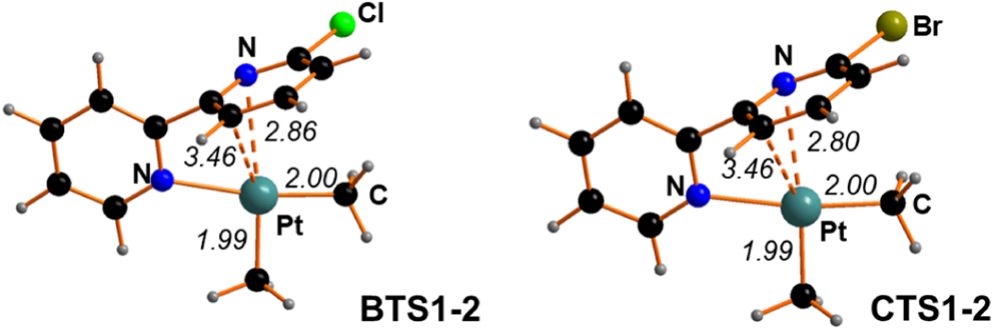
Optimized structures of transition states **BTS1–2** and **CTS1–2**. Selected bond lengths (in Å) are reported *in italics*.

The transition-state nature of **BTS1–2** and **CTS1–2** was confirmed by frequency calculations, which revealed a single imaginary frequency of −34 and −39 cm^–1^, respectively, corresponding to the cleavage of the Pt–N bond and the simultaneous formation of the new Pt–C bond. The system then evolves toward the three-coordinate intermediates **B2** and **C2** (Figure 7), which feature complete cleavage of the Pt–N bond and a T-shaped coordination geometry around the platinum center. The free-energy changes from the transition states to the corresponding intermediates were calculated to be −6.6 and −6.8 kcal mol^–1^ for the formation of **B2** and **C2**, respectively.

**Figure 7:**
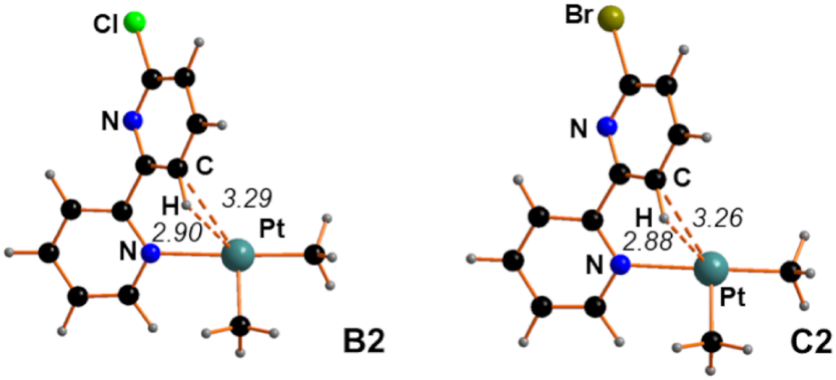
Optimized structures of intermediates **B2** and **C2**. Selected bond lengths (in Å) are reported *in italics*.

Subsequent C–H bond activation proceeds through the transition states **BTS2–3** and **CTS2–3**, with computed free-energy barriers of 15.5 and 14.9 kcal mol^–1^, respectively. This step leads to the formation of the pentacoordinate intermediates **B3** and **C3** (Figure 8), with modest free-energy gains of −4.2 and −4.7 kcal mol^–1^, respectively. In these minima, the Pt–H and Pt–C bond distances are significantly shortened by more than 1.4 and 1.2 Å, respectively, consistent with the formation of Pt–H and Pt–C bonds.

**Figure 8:**
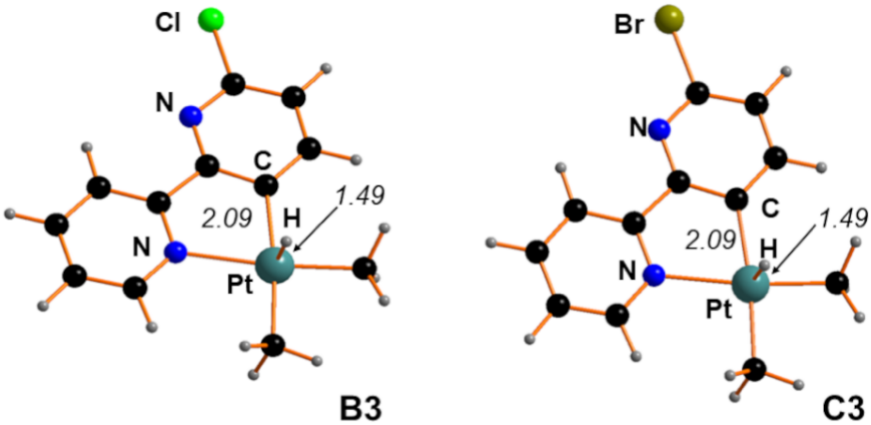
Optimized structures of penta-coordinated intermediates **B3** and **C3**. Selected bond lengths (in Å) are reported *in italics*.

The final square-planar complexes **B4** and **C4**, featuring a cyclo-metalated bipyridine ligand together with one methyl and one DMSO ligand, are formed through the transition states **BTS3–4** and **CTS3–4**, with very low computed free-energy barriers of 2.8 and 2.5 kcal mol^–1^, respectively. The optimized structures of **BTS3–4** and **CTS3–4** are shown in Figure 9. In both structures, the hydrogen atom is located between the platinum center and the methyl ligand. Accordingly, the Pt–H and Pt–C distances are elongated relative to those in the pentacoordinate intermediates **B3** and **C3**. The transition-state nature of **BTS3–4** and **CTS3–4** was confirmed by frequency calculations, which revealed a single imaginary frequency of −486 and −584 cm^–1^, respectively, corresponding to cleavage of the Pt–H bond and simultaneous formation of the C–H bond.

**Figure 9:**
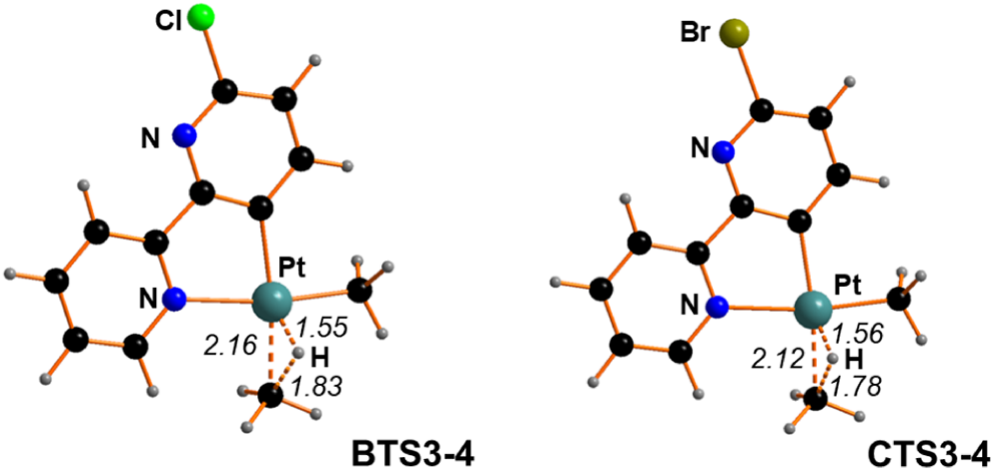
Optimized structures of transition states **BTS3–4** and **CTS3–4**. Selected bond lengths (in Å) are reported *in italics*.

The release of a methane molecule and the coordination of a DMSO one allows the formation of complexes **B4** and **C4** with large free energy gains of −38.5 and −37.0 kcal mol^–1^. DFT analysis revealed that the most stable square-planar isomers are those featuring the DMSO ligand trans to C rather than to nitrogen center with free energy stabilization larger than −18.0 kcal mol^–1^. Based on the DFT calculations, the proposed free energy pathways for the reaction between **A** with **bpy** ^**Cl**^ and **bpy** ^**Br**^ are reported in Figure 10 (black for **bpy** ^**Cl**^ and red in **bpy** ^**Br**^). The two reactions are particularly exergonic being the free energy gains as large as −27.2 and −27.4 kcal mol^–1^ for the reaction with **bpy** ^**Cl**^ and **bpy** ^**Br**^, respectively.

**Figure 10:**
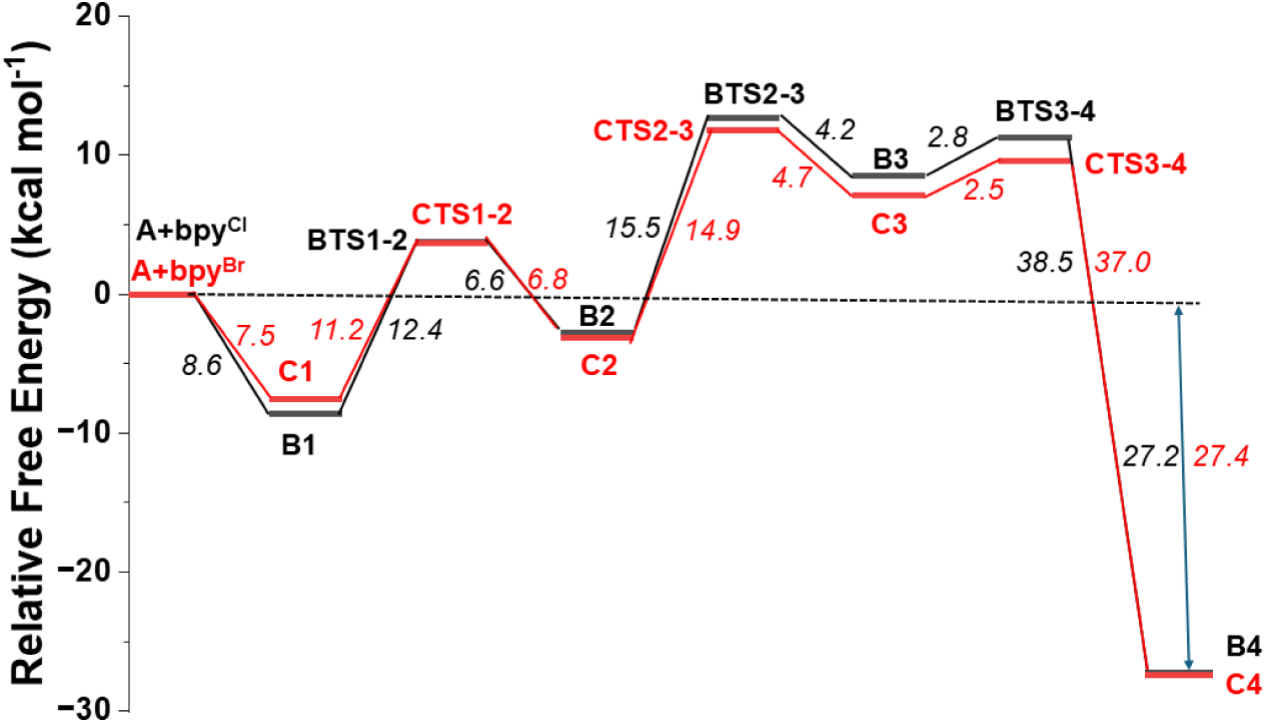
Free energy pathways (in kcal mol^–1^) for the reactivity between **A** and **bpy^Cl^** (in black) and **bpy^Br^** (in red).

### NMR Kinetic Investigation

In a previous work on rollover cyclometalation, we reported an NMR study demonstrating that the reaction between [Pt­(DMSO)_2_Me_2_] and 6-substituted 2,2′-bipyridines proceeds via a consecutive reaction.^50^ In this process, the [Pt­(N^N)­Me_2_] adduct is initially formed and subsequently converts into the rollover complex [Pt­(N^C)­(DMSO)­Me]. The reaction between [Pt­(DMSO)_2_Me_2_] (A) and the 6-substituted 2,2′-bipyridine (B) follows second-order kinetics, described by the rate law *v* = *k*[A]­[B].

In an initial attempt to simplify the kinetic analysis, the reaction between [Pt­(DMSO)_2_Me_2_] and 6-bromo-2,2′-bipyridine (bpy^Br^) was carried out under pseudo-first-order conditions by employing a large excess of bpy^Br^ (typically 10–20 equiv relative to the platinum complex). Under these conditions, the concentration of the ligand is essentially constant throughout the reaction, and the observed rate is expected to follow a first-order dependence on [Pt­(DMSO)_2_Me_2_]. However, this approach proved unsuccessful: first, the reaction is too fast to be followed by NMR and, second, in the presence of a large excess of bpy^Br^, a secondary reaction was observed, leading to the formation of a new platinum species identified as [Pt­(bpy^Br^-H)­(κ^1^-N-bpy^Br^)­Me], in which the excess bpy^Br^ displaces the coordinated DMSO ligand from the initially formed product. These factors complicated the kinetic analysis and precluded the reliable extraction of rate constants under pseudo-first-order conditions. Consequently, the kinetic study was redesigned for both ligands (bpy^Cl^ and bpy^Br^) using equimolar concentrations of [Pt­(DMSO)_2_Me_2_] and ligand ([A]_0_ = [B]_0_ = 9.0 × 10^–3^ M), as described below.

Both reactions were monitored by ^1^H NMR spectroscopy in deuterated acetone at 298 K. Spectra were acquired at regular time intervals (every 5 min over 50 min), and the concentrations of [Pt­(DMSO)_2_Me_2_] ([A]_*t*_) and the product (rollover complex, C) ([C]_*t*_) were determined by integration of the corresponding ^1^H NMR resonances relative to an internal standard. The NMR spectra showed, only initially, signals related to the adduct [Pt­(N^N)­Me_2_] (less than 1%) which likely converts rapidly to the final rollover complex.

According to our previous study, the reactions of both ligands with [Pt­(DMSO)_2_Me_2_] are expected to be bimolecular and second-order overall, with the rate law:

1 −d[A]/dt=k[A][B]

where [A] = [Pt­(DMSO)_2_Me_2_] and [B] = [ligand]. Since [A]_0_ = [B]_0_ and the reaction stoichiometry is 1:1, the condition [A]_*t*_ = [B]_*t*_ holds at all times. (eq 1) therefore reduces to −d­[A]/dt = k­[A]^2^, which integrates directly to^51,52^

2 $$1/{\left[A\right]}_{t}=1/{\left[A\right]}_{0}+k\times t$$

A plot of 1/[A]_*t*_ versus *t* is therefore expected to be linear, with slope k and intercept fixed at 1/[A]_0_ = 111.1 M^–1^. The rate constant was obtained by constrained linear regression in which the intercept was held fixed at the known value of 1/[A]_0_, and only the slope k was optimized.

Figure 11 shows the kinetic data for the reaction of [Pt­(DMSO)_2_Me_2_] with 6-chloro-2,2′-bipyridine (bpy^Cl^) and 6-bromo-2,2′-bipyridine (bpy^Br^). Panel A displays the constrained second-order plot of 1/[A]_*t*_ versus *t*, which is linear over the entire observation window (R^2^ = 0.9942), confirming the second-order kinetic model. The constrained linear regression, with the intercept fixed at 1/[A]_0_ = 111.1 M^–1^, gave a rate constant k­(bpy^Cl^) = 5.76 M^–1^ min^–1^ (0.0960 M^–1^ s^–1^). Panel B shows the evolution of [A]*_t_* and [C]*_t_* over time.

**Figure 11:**
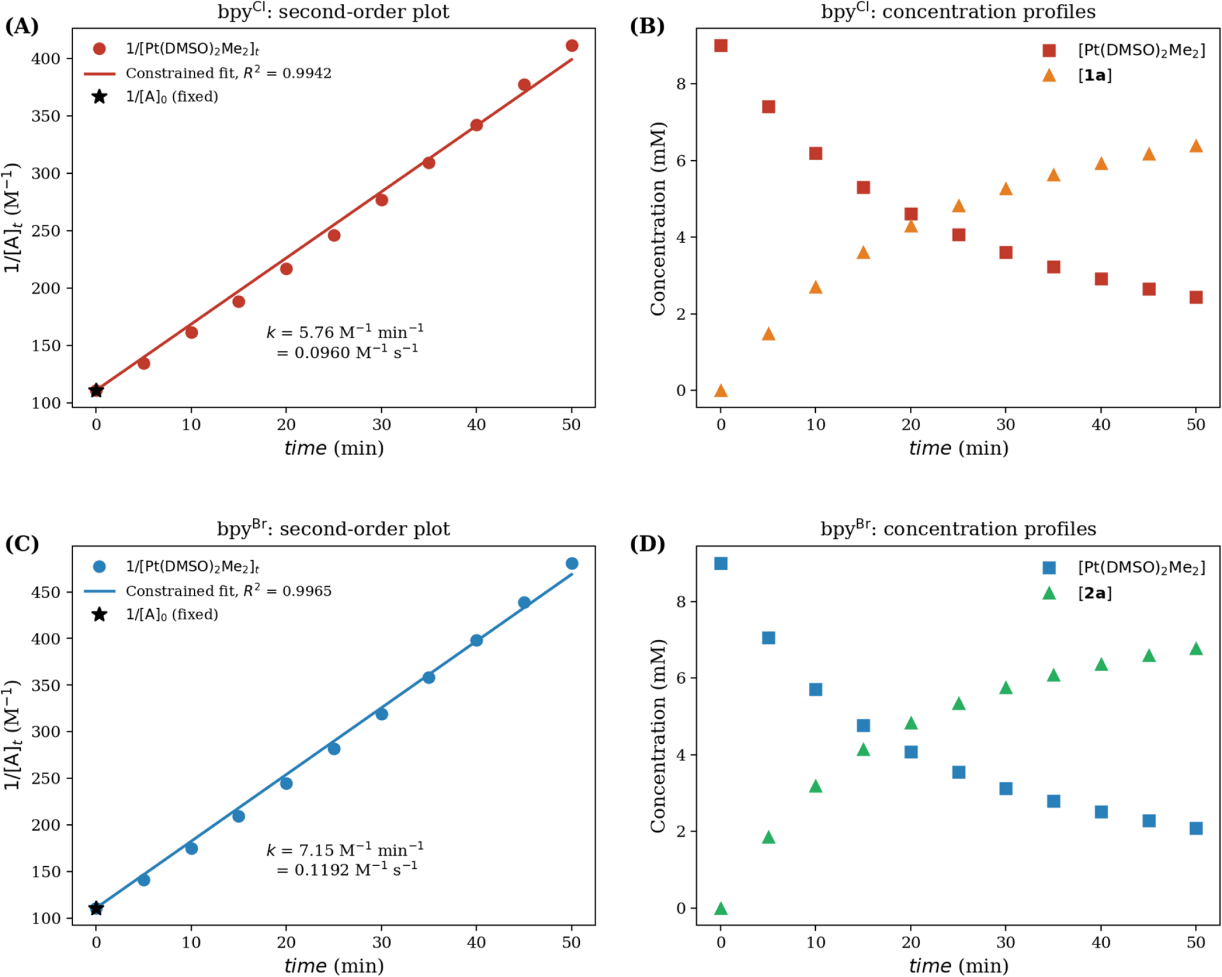
Second-order kinetic plots and concentration profiles for the reactions of Pt­(DMSO)_2_Me_2_ with 6-chloro-2,2′-bipyridine (bpy^Cl^, top row) and 6-bromo-2,2′-bipyridine (bpy^Br^, bottom row) in acetone-d6 at 298 K ([A]_0_ = [B]_0_ = 9.0 × 10^–3^ M). **Panels A and C**: plots of 1/[Pt­(DMSO)_2_Me_2_]*_t_* versus *time* for bpy^Cl^ and bpy^Br^, respectively. The solid lines are constrained linear fits with the intercept fixed at 1/[A]_0_ = 111.1 M^–1^ (★); the slope gives the second-order rate constant *k*. For bpy^Cl^: *k* = 5.76 M^–1^ min^–1^ (0.0960 M^–1^ s^–1^), R^2^ = 0.9942. For bpy^Br^: *k* = 7.15 M^–1^ min^–1^ (0.1192 M^–1^ s^–1^), R^2^ = 0.9965. **Panels B and D**: concentration profiles of Pt­(DMSO)_2_Me_2_ (■) and the respective products **1a** and **2a** (▲) as a function of *time*.

The analogous reaction with 6-bromo-2,2′-bipyridine (bpy^Br^) was conducted under identical conditions. The constrained second-order plot (Figure 11, panel C) is again linear (R^2^ = 0.9965), yielding *k*_2_(bpy^Br^) = 7.15 M^–1^ min^–1^ (0.1192 M^–1^ s^–1^).

Figure 12 provides a direct comparison of the two reactions. The rate constant for bpy^Br^ (k = 7.15 M^–1^ min^–1^) is approximately 1.24-fold larger than that for bpy^Cl^ (k = 5.76 M^–1^ min^–1^), indicating that the bromo substituent at the 6-position of the bipyridine ligand exerts a modest but measurable accelerating effect on the substitution. This trend is consistent with the greater steric hindrance of the bromo ligand relative to the chlorine, shifting the equilibrium toward the final rollover complex.

**Figure 12:**
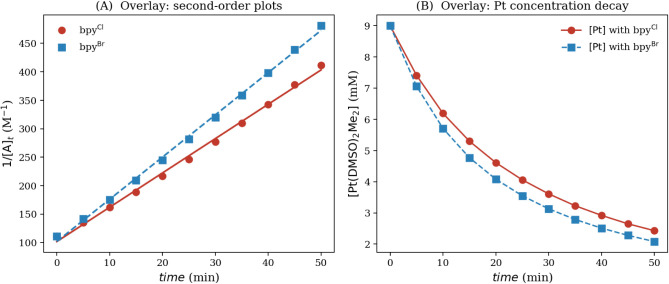
(A) Overlay of the constrained second-order kinetic plots for the reactions of Pt­(DMSO)_2_Me_2_ with bpy^Cl^ (●, red) and bpy^Br^ (■, blue) versus *time*. (B) Comparison of the Pt­(DMSO)_2_Me_2_ concentration decay for both reactions.

The Gibbs activation energy ΔG^‡^ was calculated from the Eyring–Evans–Polanyi equation of transition-state theory:

3 $$\Delta G^{‡}=RT\times \ln\left(\kappa \;k_{B}T/h\times k\times c^{{}^{\circ}}\right)$$

where k_B_ = 1.3806 × 10^–23^ J K^–1^ is the Boltzmann constant, h = 6.6261 × 10^–34^ J s is the Planck constant, R = 8.3145 J mol^–1^ K^–1^, T = 298.15 K, κ = 1 (transmission coefficient), and c° = 1 M is the standard-state concentration included to ensure dimensional consistency. The values obtained are ΔG^‡^(bpy^Cl^) = 78.83 kJ mol^–1^ and ΔG^‡^(bpy^Br^) = 78.30 kJ mol^–1^. These values are in the range typical of associative ligand substitution at square-planar Pt­(II) centers.

To evaluate the electronic effects of the halogen substituents in bpy^Cl^ and bpy^Br^ ligands, we considered the Hammett linear free-energy relationship, a well-established framework for understanding electronic modulation in aromatic systems, specifically, in our case, pyridine derivatives. For halogen substituents, standard Hammett σ constants account for their dual nature: inductive electron withdrawal (−*I* effect) countered by weak resonance π-donation +*R* effect). In the specific case of rollover cyclometalation, the activated C^3^–H bond resides in the *para* position relative to the halogen. Given that the *para* constants for chlorine and bromine are nearly equivalent (σ_*p*_ = +0.23),^53^ a comparable electronic facilitation of the C–H activation is expected for both ligands. Regarding the influence on the adjacent nitrogen atom, no reliable ortho σ scale exists for pyridine systems, since ortho substituents introduce steric interactions and coordination-geometry perturbations that may overshadow purely electronic contributions.

### Substitution Reactions

The labile DMSO ligand in both **1a** and **1b** can be readily substituted by triphenylphosphine to yield **2a**, [Pt­(bpy^Cl^-H)­(PPh_3_)­Me], and **2b**, [Pt­(bpy^Br^-H)­(PPh_3_)­Me] (Scheme 1). These reactions proceed at 60 °C in acetone, demonstrating the synthetic versatility of the DMSO precursors.

The ^1^H NMR spectra of **2a** and **2b** are consistent with the proposed formulations: the H^6’^ protons appear strongly shielded, at 8.29–8.30 ppm (**2b**), reflecting the presence of the *cis*-coordinated PPh_3_ in close proximity (Δδ between **1a**–**b** and **2a**–**b** ca. 1.40 ppm). The coordinated methyl appears as a doublet with satellites due to coupling with the *cis*-phosphorus donor (J_P–H_ = 7.6–7.7 Hz). In the ^31^P NMR spectrum a singlet with satellites at characteristic chemical shift, *ca* 32 ppm, with J_Pt–P_ = 2295 Hz for both **2a** and **2b**, confirms coordination in trans to carbon atom.

The ^13^P–^195^Pt coupling constants observed in **2a** and **2b**, in agreement with a trans P–Pt–C coordination, are relatively large when compared to similar complexes, indicating a weak *trans*-influence of the cyclometalated carbon, which is associated with the poor donor properties of the cyclometalated pyridine, due to Br and Cl substituents.

As recently reported by some of us,^391^J_Pt–P_ coupling constant values constitute a good indicator for the donor properties of cyclometalated ligands. The values of 2295 Hz found for **2a** and **2b** can be compared to those observed for the unsubstituted bpy (2229 Hz), bpy^Me^ (2226 Hz), bpy^Ph^ (2233 Hz) and bpy^CF3^ (2279 Hz) ligands (Figure 13). To the best of our knowledge, the J_Pt–P_ values reported here for **2a** and **2b** are the highest reported to date for neutral cyclometalated [Pt­(N^C)­(PPh_3_)­Me] complexes, surpassed only by cationic, protonated rollover complexes (in these cases, J_Pt–P_ up to 2500 Hz).

**Figure 13:**
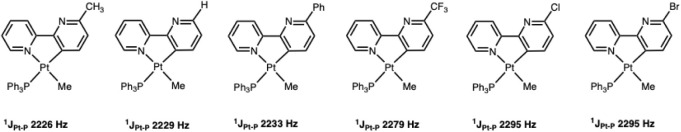
Scale of donor properties, X substituent in position 6 (^1^J_Pt–P_ values in Hz): X = Me > H > Ph > CF_3_ > Cl, Br. The indications given by ^31^P–^195^Pt coupling constants appear very useful for evaluating the donor properties of the cyclometalated ligand.

### Electron-Poorer Complexes

The methyl complexes **1a** and **1b** can be converted to the corresponding, electron-poorer, chloro derivatives by reaction with aqueous HCl; the reaction allowed isolation of [Pt­(bpy^Cl^-H)­(DMSO)­Cl], **3a**, and [Pt­(bpy^Br^-H)­(DMSO)­Cl], **3b** (Scheme 1) providing access to electron-poor alternative complexes. An efficient one-pot procedure was also employed, starting from the precursor [Pt­(DMSO)_2_Me_2_], where the initial cyclometalation is followed directly by HCl treatment, yielding the chloro complexes in good overall yields (70–75%).

As expected, in the ^1^H NMR spectrum of **3a** and **3b** the H^6′^ protons appear strongly deshielded at 9.57 ppm for both complexes, with the H^4^ proton also showing characteristic deshielding (8.46–8.56 ppm) due to proximity to the DMSO ligands. The DMSO signals (singlets with satellites) are almost unaffected in the chemical shift values, appearing at 3.64 ppm, but with larger ^3^J_Pt–H_ values (^3^J_Pt–H_ ca. 24 Hz in **3a**–**b** vs ca. 19 Hz in **1a**–**b**), due to the *trans* S–Pt–N coordination.

Crystals of complex **3b** suitable for single-crystal X-ray diffraction were obtained by slow diffusion of diisopropyl ether into a solution of the complex in chloroform. Compound **3b** crystallizes in the centrosymmetric monoclinic space group *P*2_1_/*n* and the resulting structure confirms the proposed formulation, showing rollover cyclometalation of 6-bromo-2,2′-bipyridine and coordination of the chloride ligand in *trans* with respect to the carbon donor of the cyclometalated bipyridyl ligand (Figure 14). The complex displays a square planar coordination geometry around the metal center. The Pt–N bond distance is typical of analogous bipyridyl complexes [Pt(1)–N(2) 2.072(9) Å], and so is the Pt–C distance [1.985(10) Å] compared with analogous cyclometalated complexes previously reported in the literature, for instance, the Pt–C distance in the cyclometalated complex [Pt­(bpy^tBu^)­Cl­(SMe_2_)] (Hbpy^tBu^ = 6-*tert*-butyl-2,2′-bipyridine) [1.990(5) Å].^54^ If we extend these comparisons to complexes containing different halide donors and neutral coligand (*e.g.*, phosphines), the cyclometalated Pt–C distances are, generally, statistically equivalent to **3b**, *e.g.*, 2.011(3) Å in [Pt­(bpy-H)­(Cl)­(PPh_3_)].^55^ The complex presents π-stacking interactions between the pyridyl rings not involved in the cyclometalation [3.796(9) Å], with the Pt centers placed normal to one of the pyridyl rings of the neighboring complex unit [Pt···ring 3.864(5) Å]. Finally, two weak hydrogen bonding interactions can be detected between the H^6’^ proton and respective Cl ligand [H(10)···Cl(1) 2.571 Å], and the H^3′^ proton with DMSO ligand [H(2)···O(1)] 2.213 Å with distances below the sum of the van der Waals radii (*r*_H_ = 1.10 Å, *r*_Cl_ = 1.76 Å, *r*_O_ = 1.58 Å) in all cases. Both hydrogen bonding interactions could be considered relatively weak, as evidenced by their significant deviation from the ideal X–H···D angle of 180° [C10–H(10)–Cl(1) 126.0°; C2–H(2)–O(1) 140.0°],^56^ and likely driven by the constrained position of the C–H bonds and acceptors (O and Cl) in **3b**.

**Figure 14:**
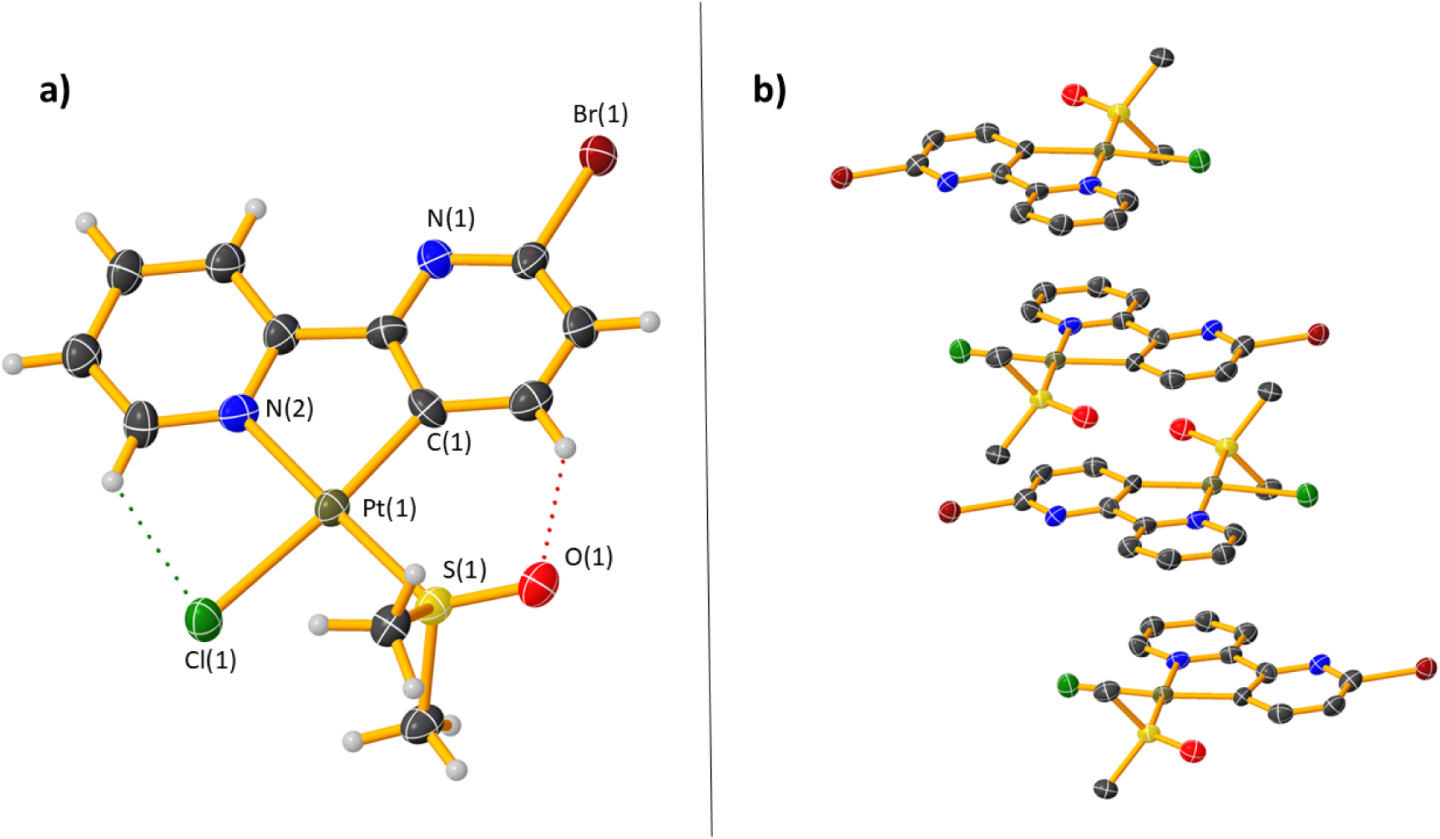
Molecular structure of **3b**: (a) asymmetric unit with selective labeling and (b) long-range π-stacking interactions. Ellipsoids are shown at 30% probability level and hydrogens have been removed for clarity in the π-stacking representation.

All structural parameters are summarized in Table S1.

Treatment of [Pt­(bpy^X^-H)­(DMSO)­Cl] complexes with triphenylphosphine at room temperature yielded [Pt­(bpy^Cl^-H)­(PPh_3_)­Cl] (**4a**), and [Pt­(bpy^Br^-H)­(PPh_3_)­Cl] (**4b**). The^1^ H NMR spectra show the expected high-field shift of the H^4^ protons (e.g., δ = 6.77 ppm in **4a**) due to proximity of PPh_3_, and are also coupled to both ^195^Pt and ^31^P nuclei. Finally, the ^31^P NMR spectra showed a singlet with satellites with values in line with the proposed formulation (see experimental).

It is worth noting that complexes **1**-**4** may exist as two geometric isomers; as an example, complexes **1a** and **1b** may have the methyl coordinated in *trans* to the nitrogen or to the cyclometalated carbon. However, as previously reported for similar [Pt­(N^C)­(L)­X] derivatives, only one isomer is always formed and observed, *i.e.*, the one with the methyl group trans to the pyridine nitrogen atom,^43,57,58^ owing to the large difference in *trans* influence between the coordinated ligands. For this reason, once N^C coordination is fixed, the methyl ligand (*i.e.*, the ligand with the highest *trans*-influence) adopts coordination in *trans* to the donor with lowest *trans*-influence, *i.e.*, the pyridyl nitrogen. The opposite happens for the chloride ligand in complexes **3** and **4**.

With the intention of enhancing the hydrophilicity of one complex of this series we employed the water-soluble phosphine PTA (PTA = 1,3,5-triaza-7-phosphaadamantane) to synthesize [Pt­(bpy^Br^-H)­(PTA)­Me], **5b**. The use of PTA was intended to enhance aqueous solubility for potential biological applications. However, despite the hydrophilic nature of PTA, the resulting complex **5b** remained sparingly soluble in water. As for the analogous complex **2b**, the coordinated methyl signal in the ^1^H NMR spectrum appears as a doublet with satellites (δ 0.87 ppm, J_P–H_ = 7.8 Hz), due to coupling with the phosphorus coordinated in *cis* position. In the ^13^P NMR spectrum a singlet with satellites at −65.91 ppm (^1^J_Pt–P_ = 1971.9 Hz) confirms PTA coordination (Scheme 2).

**Scheme 2:**
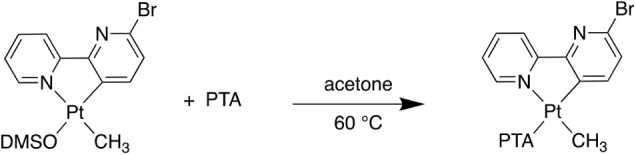
Synthesis of Complex **5b**

### Electrochemistry

The voltammetric behavior of complexes **1a**–**b** – **4a**–**b** (Table 3) was first investigated in dichloromethane to avoid coordination by solvent molecules. No significant differences were observed between the bpy^Cl^ and the bpy^Br^ derivatives. No electrode process is detectable in the available potential window for DMSO-derivatives **1a**–**b** and **3a**–**b**, maybe occurring at potential values higher than the solvent system oxidation. On the other hand, in the case of **2a**–**b** and **4a**–**b**, where PPh_3_ is one of the ancillary ligands, an irreversible anodic process can be observed very close to the solvent system oxidation (0.96–0.98 V and 1.14–1.17 V respectively, at 100 mV s^–1^ as the potential scan rate). Cyclic voltammetry experiments performed between 20 and 500 mV s^–1^ show an anodic shift of the peak potentials at increasing scan rate, as expected for a nonreversible process (Figure S59). Moreover, the linear relationship between peak current (i_p_) and potential scan rate square root (v^1/2^) (Figure S59, inset) confirms the diffusive nature of the electrode process. Irreversible anodic processes on Pt­(II) derivatives are usually ascribed to an EC process where the charge transfer is followed by a chemical reaction to give unstable, formal Pt­(III) species. However, according to previous evidence on Pt-bpy cyclometalated complexes,^55,57,59^ the anodic process involves the metal center as well as the bpy ligand and also chloride, when it acts as monodentate ligand (as in the case of **4a**–**b**). No response in the direct cathodic scan was evident for any of the compounds.

**Table 3:** Voltammetric Data of Complexes **1a**–**b** – **4a**–**b** in CH_2_Cl_2_/0.1 M TEAPF_6_ and CH_3_CN/0.1 M TEAPF_6_ Solvent System^a^

Complex	Ep (V), CH_2_Cl_2_-TEAPF_6_	Ep (V), CH_3_CN-TEAPF_6_
Pt(bpy^Cl^-H)(DMSO)Me] (1a)	n.d.	0.05 + 0.15 (overlapping)
Pt(bpy^Br^-H)(DMSO)Me] (1b)	n.d.	–0.03 + 0.18 (overlapping)
Pt(bpy^Cl^-H)(PPh_3_)Me] (2a)	0.96	0.37 (shoulder) 0.91
Pt(bpy^Br^-H)(PPh_3_)Me] (2b)	0.98	0.27 (shoulder) 0.85
Pt(bpy^Cl^-H)(DMSO)Cl] (3a)	n.d.	0.50 (broad)
Pt(bpy^Br^-H)(DMSO)Cl] (3b)	n.d	0.77 (broad)
Pt(bpy^Cl^-H)(PPh_3_)Cl] (4a)	1.14	0.59 + 0.71 (overlapping)
Pt(bpy^Br^-H)(PPh_3_)Cl] (4b)	1.17	0.58

a Potential values referred to *E*_1/2_ of the redox couple Fc/Fc^+^.

With the aim to thoroughly explore the oxidation processes of these complexes, a further CV characterization was carried out using acetonitrile instead of dichloromethane as the solvent, widening the accessible potential window. Different behaviors were observed by varying the set of ligands other than bpy. When DMSO and Me are the ligands (complexes **1a**–**b**), two very close anodic processes are observed between −0.05 and 0.20 V (potential scan rate: 100 mV s^–1^), poorly reproducible in position and respective intensity at the beginning of the voltammetric experiment, with an associated cathodic process at −0.69 V (Figure S60). Such a behavior suggests the exchange of DMSO with the stronger ligand CH_3_CN, as supported by NMR spectra. To ascertain this point, we recorded the ^1^H NMR spectra of complexes **1a** and **1b** in CD_3_CN, observing the complete release of the coordinated DMSO (singlet at 2.53 ppm, integrating to 6H, corresponding to free DMSO). For both complexes, only a single species is observed in solution, showing ^195^Pt satellites for both the methyl group and the H^6^ and H^3^ hydrogens of the bipyridine ligands; this demonstrates that the Pt­(N^C)­Me scaffold remained intact. Most likely, the species observed are the two rollover complexes [Pt­(bpy^Cl^)­(CD_3_CN)­Me] and [Pt­(bpy^Br^)­(CD_3_CN)­Me]. Both spectra are included in the Supporting Information (Figures S55–S58). The resulting electron-richer species probably arranges in two different forms that originate overlapping peaks at low potentials (−0.05 – 0.20 V). When the couple of ancillary ligands is DMSO/Cl (complexes **3a**–**b**), only a very broad peak, with low intensity, is observed at about 0.6 V, reasonably ascribable to two concomitant events: (i) the exchange of the DMSO ligand with the solvent, and (ii) the oxidation of the chloride ligand. On the other hand, complexes **2a**–**b** (Figure S61), bearing PPh_3_ and -Me as the ligands, show an anodic process at 0.91 V (**2a**) and 0.85 V (**2b**), respectively, with a shoulder at 0.37 V (**2a**) and 0.27 V (**2b**). An associated cathodic peak is detected in the reverse scan at −0.79/–0.81 V. The main anodic voltammetric process is a diffusive-type, as evidenced by the i_p_/v^1/2^ linear relationship (Figure S61, inset). According to the comparison with the behavior in methylene chloride, this process can be ascribed to the main species, whereas the preceding shoulder is reasonably due to the species originated by the exchange of the triphenylphosphine ligand with the solvent molecules, as already observed for the analogues **1a**–**b**. A similar behavior is shown by **4a**–**b** (Figure S62), where PPh_3_ and -Cl are the monodentate ligands. In particular, the voltammetric response of **4a** shows two overlapping anodic peaks at 0.59 and 0.71 V, whereas a single peak at 0.58 V is detected in the case of **4b**. Furthermore, in this last case a reverse, broad, cathodic peak is visible around −0.50 V. Also in the case of **4a**–**b**, the charge-transfer process is assumed to be diffusive, according to i_p_/v^1/2^ curve (Figure S62, inset). Following the same argument previously mentioned, the voltammetric system can be ascribed to a partial exchange PPh_3_/MeCN combined with the oxidation of the chloride ligand.

### Antimicrobial Activity

This initial series of four pairs of rollover complexes, [Pt­(bpy^X^-H)­(L)­X] (**1a**–**b** – **4a**–**b**), was comprehensively characterized through a multianalytical approach, including NMR spectroscopy, X-ray crystallography, cyclic voltammetry, and an evaluation of the ligands’ stereoelectronic parameters. Building upon this structural foundation, the study was extended to a preliminary assessment of their antimicrobial activity, a research direction necessitated by the escalating global health threat of antimicrobial resistance (AMR).

The widespread use and misuse of conventional antibiotics has driven the emergence of highly resistant pathogens, underscoring the urgent need for novel antimicrobial classes structurally and chemically distinct from existing commercial drugs. In this context, noble metal complexes emerge as promising candidates due to their often multimodal mechanisms of action, which can significantly hinder the development of bacterial resistance.

For the biological screening, complex **5b** was included alongside the initial eight compounds. Consequently, these rollover complexes were evaluated for their antimicrobial efficacy against a selection of Gram-positive, Gram-negative multiresistant bacteria and a yeast, specifically targeting critical strains identified by the World Health Organization (WHO).

The newly synthesized compounds, characterized in terms of their chemical properties, were evaluated for: (i) antibacterial activity against bacteria grown in liquid medium under planktonic conditions; and (ii) antimicrobial susceptibility against bacteria cultured *in vitro* as biofilms.

The antimicrobial activity of compounds [Pt­(bpy^Cl/Br^-H)­(DMSO)­Me] (**1a** and **1b**), [Pt­(bpy^Cl/Br^-H)­(PPh_3_)­Me] (**2a** and **2b**), [Pt­(bpy^Cl/Br^-H)­(DMSO)­Cl] (**3a** and **3b**), Pt­(bpy^Br^-H)­(PPh_3_)­Cl] (**4a** and **4b**), and [Pt­(bpy^Br^-H)­(PTA)­(Me)] (**5b**), were tested on Gram-positive (*Staphylococcus aureus* *MDR* and *Enterococcus faecalis*), Gram-negative (*Klebsiella pneumoniae*, *Escherichia coli* and *Pseudomonas aeruginosa*) bacteria and a yeast (*Candida albicans*). The compounds [Pt­(bpy^Br^-H)­(DMSO)­Me] (**1b**) and [Pt­(bpy^Br^-H)­(PPh_3_)­Me] (**2b**) did not exhibit antimicrobial activity against *E. coli*, *S. aureus*, *K. pneumoniae*, *C. albicans*, *E. faecalis*, and *P. aeruginosa*.

Primary antimicrobial investigations were performed in accordance with the procedures described by the Clinical and Laboratory Standards Institute (CLSI).

An initial screening was carried out using the agar diffusion (Kirby–Bauer) method. Based on this assay, the compounds showed no activity against *E. coli*, *K. pneumoniae*, *P. aeruginosa*, *E. faecalis*, or *C. albicans*, with inhibition zones of approximately 0 mm. However, activity against the Gram-positive bacterium *S. aureus* was observed for only two compounds: [Pt­(bpy^Cl^-H)­(DMSO)­Me] (**1a**) and [Pt­(bpy^Br^-H)­(PTA)­(Me)] (**5b**) (Table 4).

**Table 4:** Antimicrobial Profile of *S. Aureus* ^a^

Strain	Compound	Inhibition diameter (mm)
*S. aureus*	[Pt(bpy^Cl^-H)(DMSO)Me] (**1a**)	15
*S. aureus*	[Pt(bpy^Br^-H)(PTA)(Me)] (**5b**)	16

a Inhibition diameter of Kirby-Bauer test for complexes **1a** and **5b** (C = 0.01 M).

The antimicrobial evaluation was further extended to the determination of minimum inhibitory concentration MIC, minimum bactericidal concentration MBC, and biofilm inhibition/eradication parameters, as reported in Table 5.

**Table 5:** Minimum Inhibitory Concentration (MIC), Minimum Bactericide Concentration (MBC), and Antibiofilm Assays (MBIC) Values (mol/L) for Complexes **1a** and **5b**

	1a			5b		
Compound	MIC	MBC	MBIC	MIC	MBC	MBIC
*E. faecalis*	1.25 × 10^–3^	1.25 × 10^–3^	1.25 × 10^–3^	5 × 10^–3^	5 × 10^–3^	5 × 10^–3^
*E. coli*	>1 × 10^–2^	>1 × 10^–2^	>1 × 10^–2^	2.5 × 10^–3^	2.5 × 10^–3^	2.5 × 10^–3^
*K. pneumoniae*	2.5 × 10^–3^	2.5 × 10^–3^	2.5 × 10^–3^	5 × 10^–3^	5 × 10^–3^	5 × 10^–3^
*P. aeruginosa*	2.5 × 10^–3^	2.5 × 10^–3^	2.5 × 10^–3^	2.5 × 10^–3^	2.5 × 10^–3^	2.5 × 10^–3^
*C. albicans*	>1 × 10^–2^	>1 × 10^–2^	>1 × 10^–2^	>1 × 10^–2^	>1 × 10^–2^	>1 × 10^–2^
*S. aureus* MDR	6.25 × 10^–4^	6.25 × 10^–4^	6.25 × 10^–4^	1.25 × 10^–3^	1.25 × 10^–3^	1.25 × 10^–3^

The different outcomes of the Kirby-Bauer test, the antimicrobial test, and the antibiofilm test may be related to limited diffusion of the compounds through the agar matrix due to steric hindrance which stopped the tested substances from diffusing.

It is important to emphasize that this discrepancy between the agar diffusion method (Kirby–Bauer) and liquid-based assays (MIC) is a common phenomenon observed with bulky metal complexes, where the physical properties of the compounds, such as solubility and diffusion rates within the agar matrix, do not always correlate with their intrinsic biological activity in solution. Therefore, the MIC, MBC, and MBIC values reported herein must be considered the most reliable measures of the intrinsic antimicrobial efficacy of these complexes, as the Kirby–Bauer assay primarily reflects the transport properties of the metal complexes within a solid medium rather than their actual pharmacological potential.

Overall, compound **1a** exhibited superior antibacterial potency compared to compound **5b**, as evidenced by its consistently lower MIC values against most tested strains. In particular, compound **1a** showed enhanced activity against *E. faecalis*, *K. pneumoniae*, and *S. aureus* MDR, with MIC values 2–4-fold lower than those of compound **5b**. Notably, the highest activity was observed against *S. aureus* MDR (MIC = 6.25 × 10^–4^ mol·L^–1^), highlighting the strong efficacy of compound **1a** against resistant Gram-positive pathogens.

In contrast, compound **5b** demonstrated a broader antibacterial spectrum, retaining activity against *E. coli* (MIC = 2.5 × 10^–3^ mol·L^–1^), whereas compound **1a** was inactive against this strain (MIC > 1 × 10^–2^ mol·L^–1^). This suggests that structural differences between the two compounds may influence permeability or target interactions, particularly in Gram-negative bacteria. Importantly, MIC, MBC, and MBIC values were identical for all susceptible strains, indicating that both compounds exert a bactericidal effect and are equally effective in inhibiting biofilm formation.

The antimicrobial screening of complexes **2a**, **3a**, **4a**, and **3b** (Tables 6, 7) revealed overall moderate activity with compound-dependent and strain-dependent effects. Complex **2a** showed the most balanced profile, exhibiting comparable MIC, MBC, and MBIC values (2.5–5 × 10^–3^ mol·L^–1^ and 5 × 10^–3^ mol·L^–1^) across both Gram-negative (*E. coli*, *K. pneumoniae*, *P. aeruginosa*) and Gram-positive (*E. faecalis*) strains, indicating a broad but moderate antimicrobial effect.

**Table 6:** Minimum Inhibitory Concentration (MIC), Minimum Bactericide Concentration (MBC), and Antibiofilm Assays (MBIC) Values (mol/L)

	**2a**			**3a**		
Compound	MIC	MBC	MBIC	MIC	MBC	MBIC
*E. faecalis*	5 × 10^–3^	5 × 10^–3^	5 × 10^–3^	2.5 × 10^–3^	2.5 × 10^–3^	2.5 × 10^–3^
*E. coli*	5 × 10^–3^	5 × 10^–3^	5 × 10^–3^	>1 × 10^–2^	>1 × 10^–2^	>1 × 10^–2^
*K. pneumoniae*	2.5 × 10^–3^	2.5 × 10^–3^	2.5 × 10^–3^	5 × 10^–3^	5 × 10^–3^	5 × 10^–3^
*P. aeruginosa*	2.5 × 10^–3^	2.5 × 10^–3^	2.5 × 10^–3^	>1 × 10^–2^	>1 × 10^–2^	>1 × 10^–2^

**Table 7:** Minimum Inhibitory Concentration (MIC), Minimum Bactericide Concentration (MBC), and Antibiofilm Assays (MBIC) Values (mol/L)

	**4a**			**3b**		
Compound	MIC	MBC	MBIC	MIC	MBC	MBIC
*E. faecalis*	2.5 × 10^–3^	2.5 × 10^–3^	>1 × 10^–2^	2.5 × 10^–3^	2.5 × 10^–3^	>1 × 10^–2^
*E. coli*	2.5 × 10^–3^	2.5 × 10^–3^	2.5 × 10^–3^	2.5 × 10^–3^	2.5 × 10^–3^	2.5 × 10^–3^
*K. pneumoniae*	5 × 10^–3^	5 × 10^–3^	5 × 10^–3^	5 × 10^–3^	5 × 10^–3^	5 × 10^–3^
*S. aureus* MDR	5 × 10^–3^	5 × 10^–3^	5 × 10^–3^	5 × 10^–3^	5 × 10^–3^	5 × 10^–3^

In contrast, complex **3a** retained activity against *E. faecalis* and *K. pneumoniae* (MIC/MBC/MBIC = 2.5 × 10^–3^ mol·L^–1^ and 5 × 10^–3^ mol·L^–1^, respectively), but lost efficacy against *E. coli* and *P. aeruginosa*, where no activity was observed at concentrations up to 10^–2^ mol·L^–1^. This suggests a reduced permeability or lower intrinsic potency toward more resistant Gram-negative species. Complexes **4a** and **3b** displayed similar antibacterial profiles, with consistent MIC and MBC values (2.5–5 × 10^–3^ mol·L^–1^) against all tested strains, including multidrug-resistant *S. aureus*. However, both compounds showed reduced antibiofilm activity against *E. faecalis*, with MBIC values exceeding 10^–2^ mol·L^–1^, indicating a lower efficacy in disrupting established biofilms compared to planktonic cells.

Overall, the results highlight that while these complexes maintain moderate broad-spectrum antimicrobial activity, their antibiofilm performance is more limited and highly structure- and strain-dependent.

## Other Aspects of Reactivity

### Classical N^N Coordination

The classical and typical coordination mode of 2,2′-bipyridines is chelation; indeed, 2,2′-bipyridine is considered the prototypical chelating ligand.^60^ Several attempts were made to synthesize N^N chelated complexes with 6-chloro-2,2′-bipyridine and 6-bromo-2,2′-bipyridine, such as [Pt­(N^N)­(Me)_2_], [Pt­(N^N)­(Cl)­Me], and [Pt­(N^N)­(Cl)_2_] using various platinum precursors. These efforts met with limited success, highlighting the thermodynamic preference for rollover cyclometalation over N^N chelation for these ligands. Reactions with electron-rich [Pt­(DMSO)_2_Me_2_] immediately favored rollover complex formation, whereas treatment with [Pt­(DMSO)_2_MeCl] and [Pt­(DMSO)_2_Cl_2_] yielded complex mixtures of products. In the case of the reaction between [Pt­(DMSO)_2_MeCl] and bpy^Br^ the ^1^H NMR spectrum of the main component shows signals compatible with the monodentate adduct [Pt­(κ^1^ N-bpy^Br^)­(DMSO)­(Me)­(Cl)], as suggested by a coordinated DMSO ligand having two diastereotopic DMSO methyls at 3.43 and 3.44 ppm. Furthermore, the rollover complex [Pt­(bpy^Br^-H)­(DMSO)­Cl] was not identified among the products. On the whole, the acquired data indicate a certain difficulty of bpy^Cl^ and bpy^Br^ to act as chelating ligands.

Despite the challenges in forming other N^N chelates by bpy^Cl^ and bpy^Br^ ligands, [Pt­(bpy^X^)­Cl_2_] complexes were successfully synthesized, in moderate yields, using modified Morgan conditions, starting from K_2_PtCl_4_ in aqueous HCl at 100 °C. The two adducts [Pt­(bpy^Cl^)­Cl_2_], **6a**, and [Pt­(bpy^Br^)­Cl_2_], **6b**, were characterized by means of ^1^H NMR spectroscopy and, in the case of **6b**, by X-ray crystallography.

The ^1^H NMR spectra of **6a** and **6b** show the expected resonances, with, in particular, a strongly deshielded H^6′^ signal, at 9.76 (**6a**) and 9.51 ppm (**6b**), as expected by proximity with the chloride ligand.

X-ray diffraction analysis of **6b** revealed a distorted square planar coordination geometry around the platinum center. Pt–N [2.022(7) Å and 2.032(8) Å] and Pt–Cl distances [2.327(2) Å and 3.331(2) Å] are statistically equivalent to those exhibited by **3b**. However, the presence of the bromine substituent vicinal to one of the nitrogen donors is likely responsible for a slight twist of the pyridyl rings, resulting in a loss of planarity of the bipyridyl ligand which is exemplified by the torsion angles around the C(5)–C(6) bond [N(1)–C(5)–C(6)–N(2) 3.6(12)°; C(4)–C(5)–C(6)–C(7) 4.2(11)°] and the fold angle between the rings [11.8(3)°]. Typically, we would expect the Pt coordination plane and the bipyridine ligand to be coplanar;^61^ however, as a result of this distortion described above, the idealized coordination plane of the Pt center—i.e. Pt(1), Cl(1), Cl(2), N(1) and N(2)—and the mean bipyridine plane are not coplanar, exhibiting a significant fold angle of 27.3(2)°. Furthermore, the platinum center is positioned below the mean bipyridine plane (0.68 Å) and sits slightly above the mean plane described by the four donor atoms N(1), N(2), Cl(1) and Cl(2) [Pt···mean plane 0.151 Å], which in turn causes the chloride ligands to be located at a considerable distance from the ligand plane (1.32 Å and 1.83 Å). Finally, the angles between the bipyridine plane and the metal center—defined by the dihedral angles Cl(1)–Pt(1)–N(1)–C(5) (32.5(6)°) and Cl(2)–Pt(1)–N(2)–C(6) (25.0(6)°)—further highlight a substantial deviation from an ideal square planar geometry. This pronounced distortion correlates with the large ζ angle (*vide supra*) and is associated with the facile dissociation of one of the bipyridyl nitrogen atoms from the metal center, which in turn promotes rollover cyclometalation in related dimethyl complexes. Similarly to **3b**, π-stacking interactions [3.55 Å and 3.83 Å] are present which are responsible for the propagation of the structure in the lattice (Figure 15).

**Figure 15:**
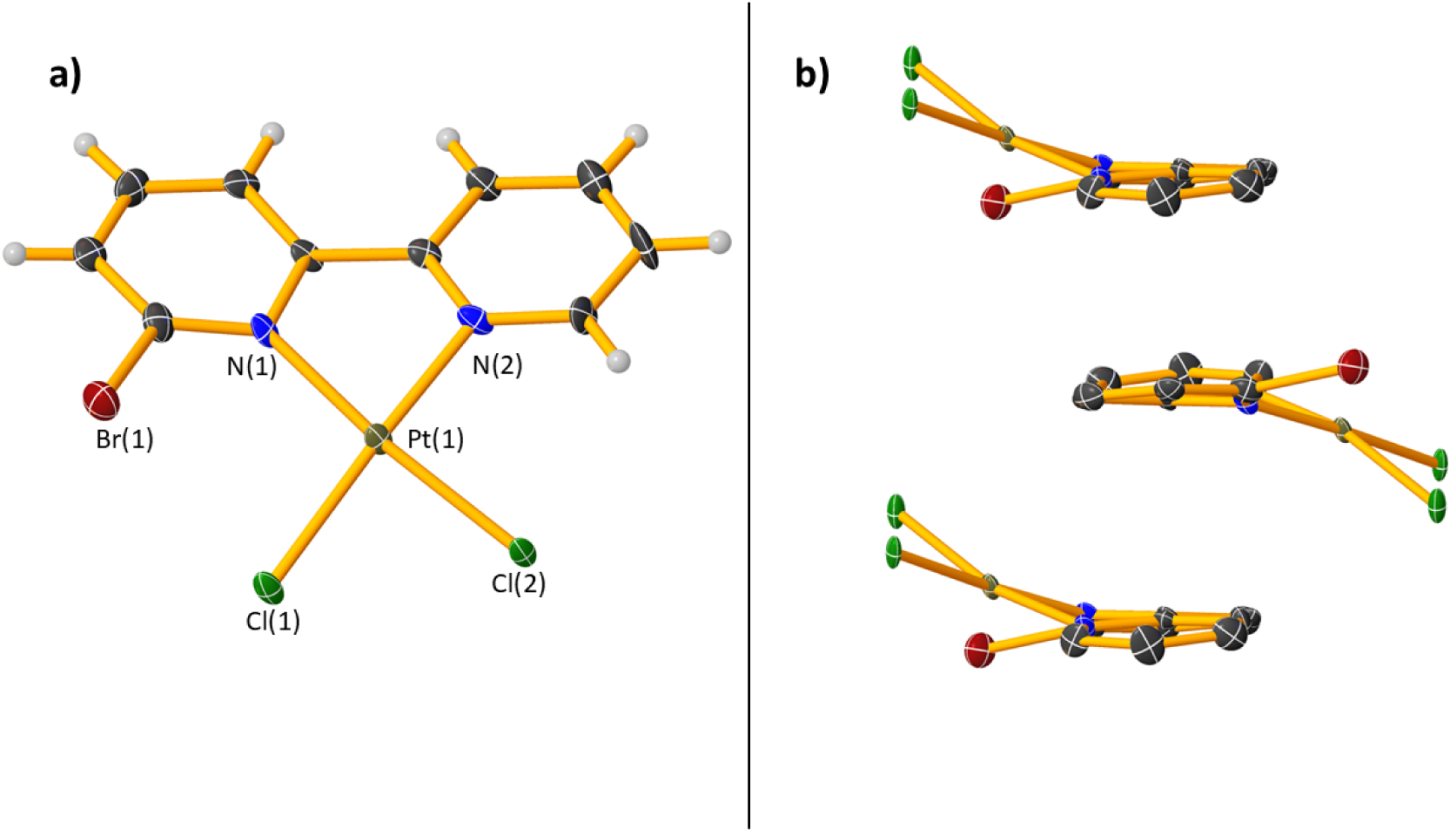
Molecular structure of **6b**: (a) asymmetric unit with selective labeling and (b) long-range π-stacking interactions. Ellipsoids are shown at 30% probability level and hydrogens have been removed for clarity in the π-stacking representation.

### Other Reactivity Aspects of Rollover Complexes

Cyclometalated complexes of formal Pt­(IV) have encountered attention in the last years.^62–67^ A useful method consists in the reaction of electron rich Pt­(II) complexes such as [Pt­(C^N)­(PR_3_)­Me] derivatives with MeI under mild conditions. The reaction rate is dependent on the basicity of the coordinated ligands, but may also occur, slowly, with scarcely donating cyclometalated complexes.

These reactions have been studied in detail by several groups, both for classical and rollover complexes. Rashidi, Nabavizadeh and coworkers conducted a series of detailed kinetic studies on methyl iodide activation by cyclometalated platinum­(II) complexes of the type [Pt­(N^C)­(PR_3_)­Me].^68–71^ The reaction with methyl iodide proceeds via a classical S_N_2 mechanism, as proved by second-order kinetics and large negative activation entropies, consistent with an associative transition state. Worth of interest, rollover complexes reacted more slowly than their classical cyclometalated counterparts derived from 2-phenylpyridine (phpy): this has been attributed to the presence of the electronegative nitrogen atom in the bpy-H ligand that renders it a slightly weaker donor compared to phpy-H.

Reaction of complexes **2a** and **2b** with methyl iodide at room temperature allowed the synthesis of Pt­(IV) complexes [Pt­(bpy^Cl^-H)­I­(Me)_2_(PPh_3_)] **7a** and [Pt­(bpy^Br^-H)­I­(Me)_2_(PPh_3_)] **7b**. The complexes were isolated as impure mixtures, due to the presence of secondary species, for which successive recrystallizations were needed, allowing the isolation of pure **7a** but not of pure **7b** (Scheme 3).

**Scheme 3:**
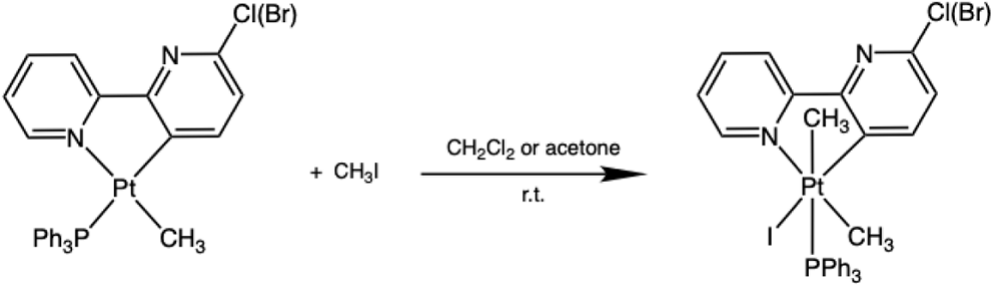
Synthesis of Complex **7a**. Complex **7b** Was Isolated as an Impure Mixture

The ^1^H and ^31^P NMR spectra of **7a** and **7b** showed characteristic NMR signals consistent with Pt­(IV) complexes: in particular, the ^1^H NMR spectra displayed two distinct methyl signals, as an example at δ 1.65 and 1.17 ppm in the case of **7b**. The Pt–H coupling constant values, of 71.0 and 53.6 Hz, respectively, are in line with methyls coordinated to Pt­(IV). The H^6′^ protons appear notably deshielded (*e.g*, 9.58 ppm in **7b**) due to the presence of a coordinated iodide in *cis* position. The ^31^P NMR spectra showed a single resonance at δ −11.50 (**7a**) and −11.56 ppm (**7b)** with platinum satellites; the Pt–P coupling constant values are in line with a Pt­(IV) coordination (J_Pt–P_ = 970 (**7a**) and 957 Hz (**7b**)).

Pt­(IV) complexes such as **7a** and **7b** might be the basis for further application, such the synthesis of newly 3,6-disubstituted bipyridines, as recently reported for similar rollover complexes.^72^

Such Pt­(IV) rollover species can be useful intermediates for the synthesis of 3-methylated 2,2′-bipyridines by reductive elimination forming a new C­(sp^2^)-C­(sp^3^) carbon–carbon bond. Attempts were made to isolate the 3-methyl-6-X-2,2′-bipyridine (X = Cl, Br) ligands from the two [Pt­(bpy^X^-H)­I­(Me)_2_(PPh_3_)] complexes by abstracting the coordinated iodide with a silver salt (AgBF_4_). The resulting pentacoordinate Pt­(IV) species is unstable and promotes a reductive elimination via C–C coupling assisted by a retro-rollover process. Subsequent treatment with dppe (dppe = bis­(1,2-diphenylphosphino)­ethane) leads to the release of the new ligands, which, however, were only observed in solution via NMR in a mixture with other species, and could not be isolated in pure form.

Although the reaction proceeds, in part, as desired, future synthetic efforts will be required to isolate these two compounds in their pure form and fully characterize them. This different behavior, compared to similar bipyridine-rollover complexes reminds us that the nature of the substituent in six-position is relevant.

A second oxidative addition reaction was performed on the complex [Pt­(bpy^Br^-H)­(PPh_3_)­Me], **2b**, using 3,5-di-*tert*-butylbenzyl bromide. In this case, the reaction proceeds differently: the oxidative addition is followed by a rapid, spontaneous reductive elimination, yielding the complex [Pt­(bpy^Br^-H)­Br­(PPh_3_)], **8b**. The ^1^H NMR spectrum of **8b** reveals the absence of signals in the aliphatic region, the presence of 21 protons in the aromatic region, and a highly deshielded H^6’^ proton at 10.02 ppm. To confirm this structural hypothesis, a ligand substitution reaction was carried out on [Pt­(bpy^Br^-H)­Cl­(PPh_3_)] with KBr in ethanol. The substitution reaction proceeded with quantitative yield to afford complex **8b** corroborating the formulation of the complex obtained via reductive elimination (Scheme 4).

**Scheme 4:**
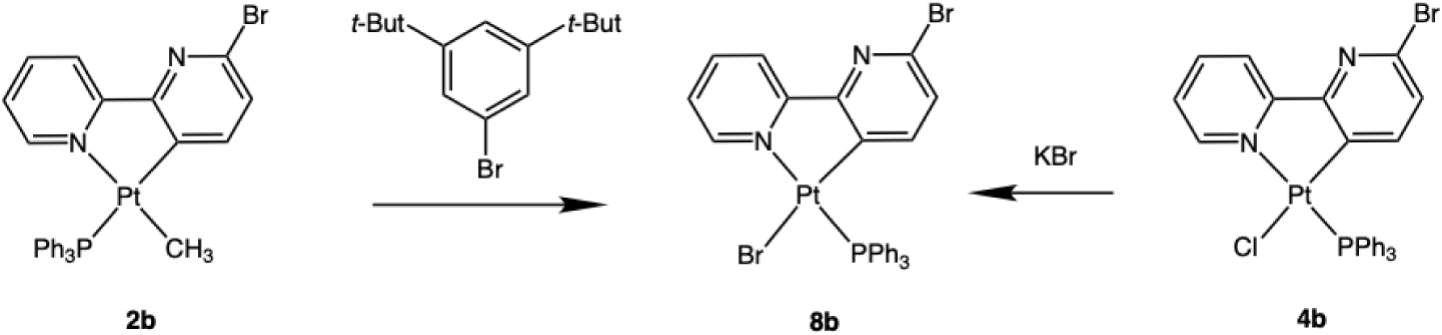
Diverse Synthetic Routes Leading to Complex **8b**

The study on the reactivity of these complexes continued with the synthesis of other cyclometalated species. Through reaction of the complex [Pt­(bpy^Br^-H)­(DMSO)­Me] with unsubstituted 2,2′-bipyridine, bpy, in the presence of [18-crown-6H_3_O]­[BF_4_] acid, in dichloromethane at room temperature, the complex [Pt­(bpy^Br^-H)­(bpy)]­[BF_4_], **9b**, is obtained (Scheme 5). As previously reported, the use of [18-crown-6-H_3_O]­[BF_4_] as a methyl abstraction agent promoted methane release, liberating a coordination site and facilitating chelation of 2,2′-bipyridine. The reaction outcome is confirmed by the absence, in the ^1^H NMR spectrum, of both the coordinated methyl and DMSO signals. In the aromatic region, instead, three signals are present relating to “H^6^” type hydrogens, *i.e.*, in positions adjacent to a pyridinic nitrogen: at δ 9.14, 8.95, and 8.67 ppm. The H^6^ signal is usually detectable in the 1H NMR spectra of bipyridines, appearing as a ddd multiplet (when well resolved), the only one with a reduced ^3^J_H–H_ *ortho* coupling with values below 6 Hz.

**Scheme 5:**
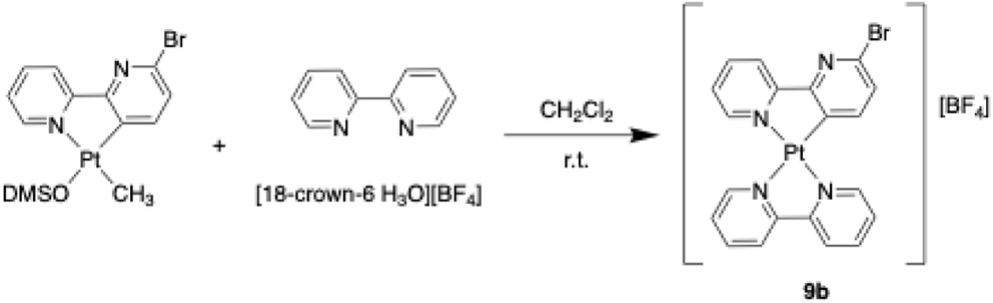
Synthesis of Complex **9b**

Complex **9b** is sparingly soluble in common organic solvents with the exception of DMSO. A more in-depth NMR study in solution was performed on this compound. The 2D NOESY/EXSY spectrum shows the existence of a dynamic process in solution that interconverts the two pyridinic rings of the unsubstituted bipyridine, at 9.14 and 8.95 ppm. This observation suggests a fluxional behavior, possibly involving partial dissociation/reassociation of the neutral bipyridine ligand. These dynamic processes for N^N chelated bipyridines or phenanthrolines have been observed in the past^14,73^ and are likely promoted by associative processes involving the solvent. Two clear NOESY cross-peaks show, inter alia, the proximity of the H^4^ proton (δ 7.66 ppm) with both H^6‴^, at 8.95 ppm and H^6″^, at 9.14 ppm (Figure 16).

**Figure 16:**
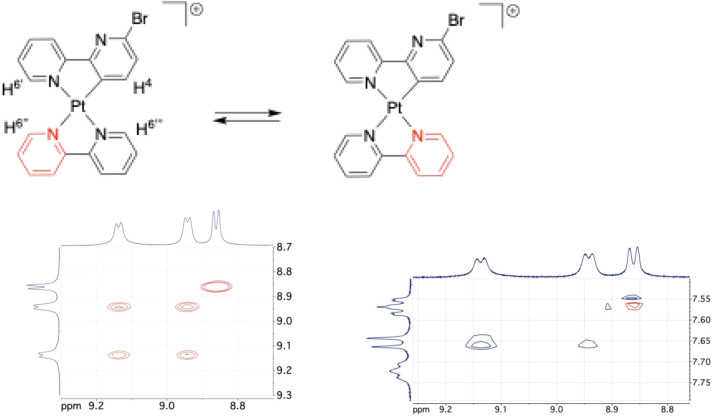
Dynamic process in solution found for complex **9b**. Below, two sections of the NOESY-EXSY NMR spectrum showing exchange cross peaks (in red, negative values) between H^6‴^ and H^6‴^ hydrogens of fluxional 2,2′-bipyridine (left), and NOESY cross-peaks) in blue, positive values) between H4, at 7.65 ppm, and H^6″^ and H^6‴^ hydrogens.

### Imine Complex Formation

We have recently found that treatment of Pt­(II) rollover complexes with ammonia gave an uncommon condensation reaction at room temperature, affording a series of rare complexes.^59^ The reaction likely occurs through a nucleophilic attack of ammonia on coordinated acetone. Operating under the same experimental conditions we obtained complexes **10a** and **10b** (Scheme 6)

**Scheme 6:**
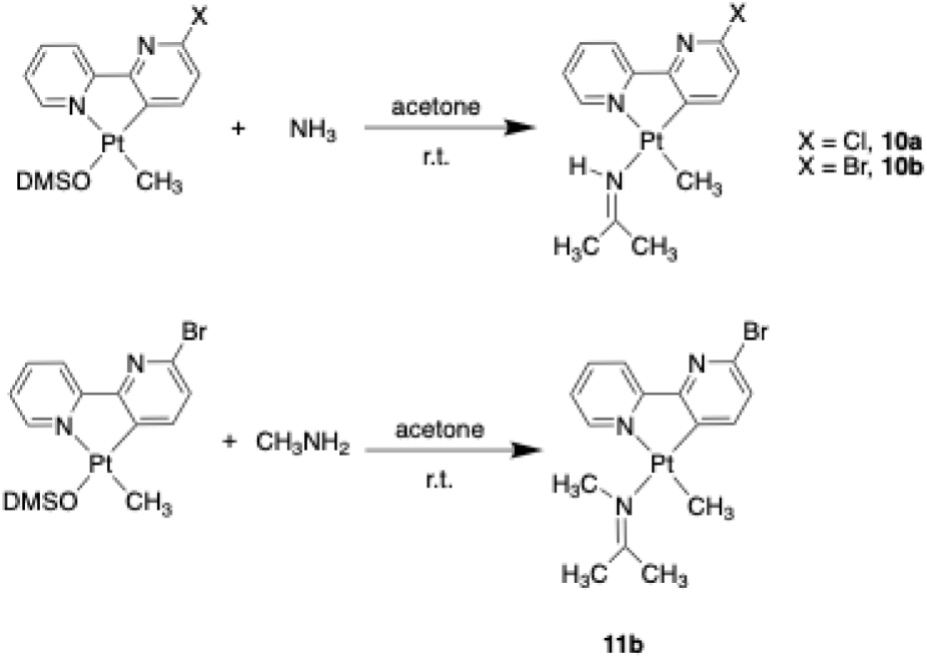
Room Temperature of Ammonia and Ammine Activation to Give Acetimino Complexes **10–11**

The ^1^H NMR spectra of **10a** and **10b** show the expected signals, such as the imine methyls around 2.28/2.30 and 2.20/2.25 ppm. The Pt-Me signal appeared in both cases at 0.77 ppm with characteristic platinum satellites (^2^ *J*_Pt–H_ = 84 Hz). The NH proton is found at low field, 9.71 (**10a**) and 9.56 (**10b**) ppm. The spectra show a difference of about 0.15 ppm between the signals of the H^4^ and H^5^ protons of the two complexes due to different anisotropic effects of Cl and Br.

Complex **10b** was obtained with a 65% yield, representing the highest yield among all cyclometalated imine synthesized complexes. This reaction represents a classic example of ligand activation at a metal center, where the coordinated DMSO ligand is displaced by acetone, which subsequently undergoes condensation with the amine nucleophile, mediated by the metal center, to form an imine ligand.

The condensation reaction may be extended to primary amines: in the case of bpy^Br^ the reaction with methylamine gave the *N*-methylated imine complex **11b**. The ^1^H NMR spectrum of **11b** showed the *N*-methyl group at δ 3.56 ppm (coupled to ^195^Pt) and two imine methyl signals at δ 2.46 and 2.16 ppm. The Pt-Me resonance appeared at δ 0.77 ppm with platinum satellites (J_Pt–H_ = 84.7 Hz), similar to **10b**. The slightly higher platinum-methyl coupling constant in **11b** compared to **10b** may reflect subtle electronic differences introduced by *N*-methylation. The ^13^C NMR spectrum of **11b** shows all the expected signals, in particular the N–CH_3_ signal at 43.19 ppm (s with sat, J_Pt–C_ = 25.0 Hz) and the other two acetimine methyls, at 29.85 and 32.71 ppm.

## Conclusions

The “rollover” cyclometalation process, where the bipyridine ligands undergo C–H activation at the 3-position, represents an important reaction for accessing novel organometallic complexes with unique bonding modes and properties. The incorporation of cyclometalated bipyridine ligands into platinum coordination spheres offers opportunities to tune the electronic and steric properties of the resulting complexes. Halogen-substituted bipyridine ligands provide additional versatility in complex design, allowing for systematic modification of the electronic properties of the metal center.

Platinum­(II) rollover complexes bearing electron-poor ligands, such as bpy^Cl^ and bpy^Br^, were readily obtained via C–H bond activation at room temperature. This reactivity can be attributed to the nucleophilic character of Pt­(II) during the rollover process, enhanced by steric and electronic properties of the ligands, which can be rationalized by means of ζ angle and p*K*_a_ and proton affinity data. The study includes a series of complexes of the general formula Pt­(N^C)­(L)­X, where the ancillary ligands (DMSO, PPh_3_, Cl, and Me) were systematically selected to provide a range of stereoelectronic properties.

This work has been extended to some aspects of reactivity, such as ligand exchange reactions, oxidative additions, treatment with acids, condensation reactions with ammonia and methylamine to give rare acetimino complexes.

Overall, the observed electronic and steric effects lead to several significant consequences:

1 Enhanced C–H bond Activation: The presence of electron-withdrawing halogens facilitates the rollover cyclometalation process by reducing the electron density at the C3 position, thereby favoring a nucleophilic activation pathway.
2 Thermodynamic Stability: The poor donor ability of the halogenated ligands reduces the basicity of the proximal nitrogen atom, promoting its detachment from the metal center. This makes the rollover cyclometalation thermodynamically more favorable compared to the classical chelation.
3 Steric Factors: The steric hindrance, as quantified by the ζ angle, further facilitates the decoordination (or prevents the coordination) of the proximal nitrogen.

Furthermore, the electronic modulation significantly alters the properties of the metal center. This effect can be effectively evaluated by observing NMR coupling constant values. Specifically, the halogen substituents markedly reduce the ligand basicity, as evidenced by the high ^1^J_Pt–P_ coupling constants in [Pt­(N^C)­(PPh_3_)­Me] complexes.

Finally, a preliminary investigation of the antimicrobial and antibiofilm activity of chosen complexes showed a moderate broad-spectrum antimicrobial activity and a more limited antibiofilm performance. The antimicrobial data presented in Tables 5–7 underscore that the antimicrobial profile is highly dependent on the ancillary ligand (L). The superior activity of **1a** ([Pt­(bpy^Cl^-H)­(DMSO)­Me]) against *S. aureus* MDR suggests that the DMSO/Me coordination sphere provides an optimal electronic environment for targeting Gram-positive cell walls. Conversely, the transition to the PTA ligand in **5b** induces a shift in selectivity, granting efficacy against *E. coli* where **1a** proved inactive. This suggests that the PTA ligand, through its specific cone angle and electronic properties, allows for a more favorable interaction with the bacterial machinery of Gram-negative strains. Regarding the influence of the 6-halogen substituent (Cl *vs* Br), our data indicate that while both halides facilitate the necessary rollover cyclometalation, the chlorine substituent in **1a** yields a more effective antimicrobial agent than its bromine-substituted counterparts in specific strains, pointing toward a subtle tuning of the platinum center’s electrophilicity by the halogen.

In a broader context, this work is part of an extensive study on a large series of chelated and cyclometalated noble metal complexes featuring pyridine-derived ligands. The data presented herein will be instrumental for a comprehensive comparison across a diverse range of complexes, characterized by various structural, steric, and electronic properties.

## Experimental Section

### General Procedures

All reactions were performed under argon atmosphere using standard techniques. Solvents were dried and purified according to standard procedures. DMSO was purified by filtration through activated alumina under argon. NMR spectra were recorded on a Bruker Avance III 400 spectrometer operating at 400.13, 100.61, and 161.97 MHz for ^1^H, ^13^C, and ^13^P, respectively. Chemical shifts (δ) are reported in ppm, referenced to TMS (^1^H and ^13^C) and H_3_PO_4_ (^31^P). NMR spectra are reported in the Supporting Information (S1–S38).

Cyclic voltammetry (CV) characterizations were performed under argon atmosphere in a single-compartment, three-electrode, electrochemical cell, using an AUTOLAB PGSTAT12 instrument interfaced with a personal computer, working with the specific software NOVA 2.1. A Pt disk (2 mm diameter) was the working electrode, Ag/AgCl with a suitable salt bridge was the reference electrode, and a graphite bar was the auxiliary electrode. The working electrode was polished with 1 and 0.3 μm alumina powder and rinsed with distilled water before each experiment. All the experiments were carried out in CH_2_Cl_2_ and in CH_3_CN (anhydrous, ≥ 99.8%, packaged under nitrogen) as solvents, using 0.1 M tetraethylammonium hexafluorophosphate (TEAPF_6_) as supporting electrolyte. All potential values are referred to the half-peak potential of the ferrocene/ferricinium ion (Fc/Fc^+^) redox couple.

### Crystallographic Methods

The crystal data for compounds **3b** and **6b** are compiled in Table S1. Crystals were examined using a Bruker D8 Quest diffractometer with a Photon III detector and a microfocus source with Cu–Kα radiation (λ = 1.54178). Intensities were integrated from data recorded on 1.0° frames by ω rotation. A multiscan method absorption correction with a beam profile was applied for both structures.^74,75^ The structures were solved using SHELXS or SHELXT;^76^ the data sets were refined by full-matrix least-squares on reflections with *F* ^2^ ≥ 2σ­(*F* ^2^) values, with anisotropic displacement parameters for all non-hydrogen atoms, and with constrained riding hydrogen geometries;^77^ *U*_iso_(H) was set at 1.2 (1.5 for methyl groups) times *U*_eq_ of the parent atom. The largest features in final difference syntheses were close to heavy atoms and were of no chemical significance. SHELX^76,77^ or OLEX2 ^78^ were employed for structure solution and refinement. The structures have been deposited with the Cambridge Crystallographic Data Centre (CCDC 2548065 and 2548066). This information can be obtained free of charge from www.ccdc.cam.ac.uk/data_request/cif.

### Computational Details

All the intermediates and the transition states have been optimized at M062X-DFT level of theory^46^ with the inclusion of the dispersion forces^47^ within the Gaussian 16 software^79^ and validated as minima and/or transition states by computed vibrational frequencies. All the calculations were based on the CPCM model^48,49^ for the acetone, the same solvent used in the experiments. The effective Stuttgart/Dresden core potential (SDD)^80^ was adopted for the platinum atom, while for all the other atomic centers the def2tzvp basis set^81^ was used. Cartesian coordinates and free energies of all the structures optimized are listed in Supporting Information Section.

### Antimicrobial Assays

Antimicrobial activity was evaluated using the standard broth microdilution method in sterile 96-well microplates. Serial dilutions of each compound (0.5%–0.0004%) prepared in nutrient broth were tested against bacterial suspensions adjusted to 1 × 10^6^ CFU/mL. After incubation for 24–48 h at 37 °C under appropriate atmospheric conditions, microbial growth was assessed by visual inspection and spectrophotometric measurement at 550 nm. The minimum inhibitory concentration (MIC) was defined as the lowest compound concentration preventing visible growth, corresponding to the absorbance of the negative control.

For determination of the minimum bactericidal concentration (MBC), aliquots from wells showing no visible growth were plated onto appropriate agar media and incubated for 24 h at 37 °C. The MBC was defined as the lowest concentration resulting in a 99.9% reduction in viable bacterial cells. All experiments were performed in triplicate.

The minimum biofilm inhibitory concentration (MBIC) was determined using a crystal violet staining assay, based on the protocol of the Montana State University Center for Biofilm Engineering with minor modifications. After 48 h of incubation under appropriate atmospheric conditions, the medium was removed and the wells were gently washed with 0.9% NaCl. Biofilms were stained with 0.1% crystal violet for 10 min, washed again to remove excess dye, and the bound stain was solubilized using 30% acetic acid. Absorbance was then measured at 620 nm using a microplate reader. The MBIC was defined as the lowest compound concentration yielding an absorbance comparable to the negative control.

### Syntheses

Synthesis of [Pt­(bpy^Cl^-H)­(DMSO)­Me], **1a**. To a solution of [Pt­(DMSO)_2_Me_2_] (154.0 mg, 0.403 mmol) in acetone (30 mL) an equimolar amount of 6-chloro-2,2′-bipyridine was added under argon atmosphere a vigorous stirring. The solution was heated to 60 °C for 1 h, then it was concentrated to small volume; the product was precipitated with hexane and filtered to yield 1a as an orange solid. Yield 90%. M.P.: 155–158 °C. Elemental Analysis calc for C_13_H_15_ClN_2_OPtS: C, 32.67%; H, 3.16%; N, 5.86%; found: C, 32.94%; H, 3.31%; N, 5.67%.^1^H NMR (CDCl_3_): δ 9.70 (ddd with sat, J_H–H_ = 5.7, 1.4, 0.7 Hz, J_Pt–H_ = 14.8 Hz, 1H, H^6′^), 8.29 (ddd, J_H–H_ = 8.0, 1.4, 0.7 Hz, 1H, H^3′^), 7.95 (td, J_H–H_ = 7.6, 1.5 Hz, 1H, H^4′^), 7.95 (d with sat, J_H–H_ = 8.0 Hz, J_Pt–H_ = 52.0 Hz, 1H, H^4^), 7.36 (ddd, 1H, H^5′^), 7.16 (d, J = 8.0 Hz, J_Pt–H_ = 16.0 Hz, 1H, H^5^), 3.26 (s, J_Pt–H_ = 18.8 Hz, 6H, DMSO), 0.69 (s, J_Pt–H_ = 81.8 Hz, 3H, Me). ^13^C NMR (CDCl_3_): δ 165.06 (C^2^), 161.14 (C^2′^), 150.42 (C^6′^), 147.87­(C^5^), 143.48 (J_Pt–C_= 1100 Hz, C^3^), 143.08 (J_Pt–C_= 94.0 Hz, C4), 138.64 (C4′), 124.97 (J_Pt–C_ = 9 Hz, C^5′^), 124.10 (J_Pt–C_= 64.4 Hz, C^5^), 121.58 (J_Pt–C_= 23.4 Hz, C^3′^), 43.80 (J_Pt–C_= 43.8 Hz, DMSO), −13.07 (J_Pt–C_= 759 Hz, Pt-Me). NMR characterization based on ^1^H, ^13^C, H–H COSY. H–H NOESY, H–C HMQC and H–C HMBC spectra.

Synthesis of [Pt­(bpy^Br^-H)­(DMSO)­Me], **1b**. Complex **1b** was obtained following the same procedure used for **1a**, adding 6-bromo-2,2′-bipyridine in place of the chloro analogue. Orange solid. Yield: 90%). M.P. = 117–120 °C. Elemental Analysis calc for C_13_H_15_BrN_2_OPtS: C, 29.89%; H, 2.89%; N, 5.36%; found: C, 30.11%; H, 3.02%; N, 5.33%. ^1^H NMR (CDCl_3_): δ 9.70 (ddd, J_H–H_ = 5.5, 1.4, 0.7 Hz, 1H, H^6′^), 8.29 (ddd, J_H–H_ = 7.7, 1.4, 0.7 Hz, 1H, H^3′^), 7.95 (td, J_H–H_= 7.2 Hz, 1H, H^4′^), 7.85 (d with sat, J = 7.9 Hz, J_Pt–H_ = 52.5 Hz, 1H, H^4^), 7.38 (ddd, J_H–H_ = 7.2, 18.0, 5.6 Hz, 1H, H^5′^), 7.33 (d with sat, J_H–H_ = 8.0 Hz, J_Pt–H_ = 15.5 Hz, 1H, H^5^), 3.26 (s with sat, 6H, J_Pt–H_ = 19.1 Hz, DMSO), 0.69 (s with sat, J_Pt–H_ = 83.5 Hz, 3H, Me) ^13^C NMR (CDCl_3_): δ 165.7, 161.0, 150.4 (C^6′^), 144.0 (C^3^, J_Pt–C_ = 1107 Hz), 142.9 (C^4^), 138.6 (C^4′^), 138.6, 127.8 (C^5^), 125.0 (C^5′^), 121.6 (C^3′^), 43.8 (DMSO, J_Pt–C_ = 43.9 Hz), −13.7 (Me, J_Pt–C_ = 769 Hz). NMR characterization based on ^1^H, ^13^C, H–H COSY. H–H NOESY, H–C HMQC and H–C HMBC spectra. ^13^C NMR selected data (acetone d6) 145.7 ppm­(C, ^3^J_Pt–C_= 1104 Hz).

General Procedure for the synthesis of [Pt­(bpy^Cl^-H)­(PPh_3_)­Me] (**2a**) and Pt­(bpy^Br^-H)­(PPh_3_)­Me] (**2b**)

**Method A.** The DMSO-containing complex **1a**–**b** (1 equiv) and the phosphine ligand (1.1 equiv) were dissolved in acetone and stirred at 60 °C for 2 h under argon atmosphere. The product was isolated by concentration and precipitation with hexane or diethyl ether with ca. 95% yield.

**Method B.** (One-pot Synthesis). A solution of [Pt­(DMSO)_2_Me_2_] (1 equiv) and **1a** or **1b** (1:1 equiv)) were dissolved in acetone and stirred under argon at 60 °C for 30 min. PPh_3_ (1:1 equiv) was then added and the reaction mixture was heated at 60 °C for 2 h. Workup as described in Method A afforded the product in 90–95% yield.

**2a** (yellow solid): M.P. 233–235 °C. Elemental Analysis calc for C_29_H_24_ClN_2_PPt: C, 52.61%; H, 3.65%; N, 4.23%; found: C, 52.81%; H, 3.30%; N, 4.47%. ^1^H NMR (CDCl_3_): δ (ppm) = 8.30 (dd with sat, 1H, J = 7.5 Hz, H6′); 8.20 (dd with sat, J_H–H_ = 8.1 Hz, J_P–H_ = 5.2 Hz, J_Pt–H_ = 47.1 Hz, 1H, H^4^); 7.80–7.68 (m, 8H, aromatics); 7.47–7.35 (m, 9H, aromatics); 7.22 (dd with sat, 1H, J = 8.5 Hz, H5); 6.68 (ddd, 1H, H5′) 0.73 (d with sat, 3H, J = 7.6 Hz, J_Pt‑Me_ = 82.8 Hz, Me). ^31^P NMR: δ (ppm) = 32.22 (s with sat, J_Pt–P_ = 2295 Hz).

**2b**: Yellow solid. M.P.: 185–188 °C. Analysis calc for C_29_H_24_BrN_2_PPt: C, 49.30%; H, 3.42%; N, 3.97%; found: C, 49.65%; H, 3.22%; N, 4.18%. ^1^H NMR (CDCl_3_): δ 8.29 (dd, J = 8.0 Hz, 1H, H6′), 8.07 (dd, J = 8.1 Hz, 1H, H4), 7.78–7.68 (m, 8H, Ar–H), 7.50–7.31 (m, 10H, Ar–H), 6.68 (ddd, 1H, H5′), 0.72 (d, J = 7.7 Hz, ^2^J_Pt–H_ = 82.7 Hz, 3H, Pt-Me). ^13^P NMR (CDCl_3_): δ 32.30 (s, ^1^J_Pt–P_ = 2294.6 Hz).

Synthesis of [Pt­(bpy^Cl^-H)­(DMSO)­Cl], **3a**. A solution of Pt­(DMSO)_2_(Me)_2_ (197.6 mg, 0.517 mmol) and bpy^Cl^ (99.0 mg, 0.519 mmol) in acetone (15 mL) was stirred at 60 °C for 30 min under argon. Then aqueous HCl (5.5 mL, 0.1 M, 0.550 mmol) was added, and the mixture was stirred at room temperature for 1 h. The resulting precipitate was collected by filtration and washed with acetone to give the first fraction as a yellow solid, yield 65%. Melting point: >250 °C. Analysis calc for C_12_H_12_Cl_2_N_2_OPtS: C, 28.93%; H, 2.43%; N, 5.62%; found: C, 28.66%; H, 2.63%; N, 5.49%. ^1^H NMR (CDCl_3_): δ 9.57 (d, J = 5.6 Hz, 1H, H6′), 8.56 (d, J = 8.5 Hz, 1H, H4), 8.21 (dd, J = 8.0 Hz, 1H, H3′), 7.98 (td, J = 14.0 Hz, 1H, H4′), 7.42 (ddd, J = 11.6 Hz, 1H, H5′), 7.09 (d, J = 8.4 Hz, 1H, H5), 3.64 (s, ^2^J_Pt–H_ = 23.9 Hz, 6H, DMSO-Me).

Synthesis of [Pt­(bpy^Br^-H)­(DMSO)­Cl], **3b**. A solution of Pt­(DMSO)_2_(Me)_2_ (153.1 mg, 0.401 mmol) and bpy^Br^ (99.1 mg, 0.421 mmol) in acetone (15 mL) was stirred at 60 °C for 30 min under argon. Then aqueous HCl (4.5 mL, 0.1 M, 0.450 mmol) was added, and the mixture was stirred at room temperature for 1 h. The resulting precipitate was collected by filtration and washed with acetone to give the first fraction as a yellow solid (38.5 mg, 0.240 mmol, 65% yield). Melting point: > 250 °C. Analysis calc for C_12_H_12_BrClN_2_OPtS: C, 26.56%; H, 2.23%; N, 5.16%; found: C, 28.79%; H, 2.65%; N, 5.29%. ^1^H NMR (CDCl_3_): δ 9.57 (d, J = 5.8 Hz, 1H, H6′), 8.46 (d, J = 8.3 Hz, 1H, H4), 8.21 (dd, J = 8.0 Hz, 1H, H3′), 7.98 (td, J = 14.0 Hz, 1H, H4′), 7.41 (ddd, J = 11.6 Hz, 1H, H5′), 7.23 (d, J = 8.3 Hz, 1H, H5), 3.64 (s, ^2^J_Pt–H_ = 23.8 Hz, 6H, DMSO-Me).

One-Pot Synthesis of Chloro Complexes [Pt­(bpy^Cl^-H)­(DMSO)­Cl] **3a** and [Pt­(bpy^Br^-H)­(DMSO)­Cl] **3b**.

For the synthesis of [Pt­(bpy^X^-H)­(DMSO)­Cl], **3a**–**b**, the initial cyclometalation reaction was followed by treatment with HCl (0.1 M solution) at room temperature for 4 h. The products were isolated by filtration and characterized by NMR spectroscopy. Typical yields 60–65% for the one-pot procedure.

Compound [Pt­(bpy^Cl^-H)­(PPh_3_)­Cl], **4a**. [Pt­(bpy^Cl^-H)­(DMSO)­Cl] (22.6 mg, 0.046 mmol) and PPh_3_ (12.6 mg, 0.046 mmol) were dissolved in acetone (8 mL) and stirred at room temperature for 1 h. Standard workup afforded the product as a yellow solid (27.1 mg, 0.040 mmol, 86% yield). Melting point: >250 °C. ^1^H NMR (CDCl_3_): δ 9.83 (m, 1H, H6′), 8.26 (d, J = 8.2 Hz, 1H, H3′), 7.97 (td, J = 7.6 Hz, 1H, H4′), 7.82–7.71 (m, 6H, Ar–H ortho), 7.50–7.35 (m, 10H, Ar–H meta/para + H5′), 6.77 (dd, J = 8.3 Hz, 1H, H4), 6.44 (d, J = 8.2 Hz, 1H, H5). ^13^P NMR (CDCl_3_, 121 MHz): δ 21.76 (s, ^1^J_Pt–P_ = 4248 Hz).

Synthesis of [Pt­(bpy^Br^-H)­(PPh_3_)­Cl], **4b**. [Pt­(bpy^Br^-H)­(DMSO)­Cl] (111.2 mg, 0.205 mmol) and PPh_3_ (57.6 mg, 0.220 mmol) were dissolved in acetone (8 mL) and stirred at room temperature for 1 h. Standard workup afforded the product as a yellow-green solid (255.2 mg, 0.382 mmol, 75% yield). Melting point: 233–235 °C. ^1^H NMR (CDCl_3_): δ 9.85 (m, 1H, H6′), 8.28 (d, J = 8.0 Hz, 1H, H3′), 0.88 (td, J = 7.6 Hz, 1H, H4′), 7.79 (m, 6H, Ar–H ortho), 7.52–7.38 (m, 10H, Ar–H meta/para + H5′), 6.69 (dd with sat (very broad, J = 8.3 Hz, 1H, H4), 6.60 (d, J = 8.2 Hz, 1H, H5). ^13^P NMR (CDCl_3_, 121 MHz): δ 21.73 (s, ^1^J_Pt–P_ = 4252 Hz).

Synthesis of [Pt­(bpy^Br^-H)­(PTA)­Me], **5b**. [Pt­(bpy^Br^-H)­(DMSO)­Me] (130.8 mg, 0.250 mmol) and PTA (40.3 mg, 0.256 mmol) were dissolved in acetone (8 mL) and stirred at 60 °C under argon for 5 h. The solution was concentrated under reduced pressure and treated with diethyl ether. Filtration of the precipitate formed afforded the product as a light orange solid (55% yield). Melting point: > 250 °C. ^1^H NMR (CDCl_3_): δ 8.46 (dd, J = 5.6 Hz, 1H, H6′), 8.32 (d, J = 7.5 Hz, 1H, H3′), 7.95 (m, 2H, H4′ + H4), 7.34 (dd, J = 7.8 Hz, 1H, H5), 7.28 (ddd, 1H, H5′), 4.58 (m, 6H, CH_2_–PTA), 4.32 (s, 6H, CH_2_–PTA), 0.87 (d, J = 7.8 Hz, ^2^J_Pt–H_ = 81.3 Hz, 3H, Pt-Me). ^13^P NMR (CDCl_3_): δ −65.91 (s, ^1^J_Pt–P_ = 1971.9 Hz).

Synthesis of [Pt­(bpy^Cl^)­Cl_2_], **6a**. A solution of Pt­(DMSO)_2_(Cl)_2_ (104.6 mg, 0.248 mmol) and bpy^Cl^ (48.7 mg, 0.255 mmol) in acetone (10 mL) was stirred at 60 °C for 72 h. The precipitate was collected by filtration to afford the product as a yellow solid (52.5 mg, 0.115 mmol, 46% yield). Melting point: > 250 °C. ^1^H NMR (CDCl_3_): δ 9.76 (d, J = 4.9 Hz, 1H, H6′), 8.25 (dt, J = 7.5 Hz, 2H, H4′ + H5′), 8.18 (td, J = 14.5 Hz, 1H, H3′), 8.05 (t, J = 8.1 Hz, 1H, H4), 7.64 (dd, J = 7.9 Hz, 1H, H5), 7.57 (m, 1H, H3).

Synthesis of [Pt­(bpy^Br^)­Cl_2_], **6b**. [Pt­(DMSO)_2_Cl_2_] (100.2 mg, 0.237 mmol) and 6-bromo-2,2′-bipyridine (56.1 mg, 0.239 mmol) in acetone were stirred for 48 h at 60 °C. The product was isolated by concentration, precipitation with ether, and filtration. Yield: 30%. Melting Point: > 250 °C ^1^H NMR (Acetone-d): δ 9.51 (d, J = 4.9 Hz, 1H, H6′), 8.55 (dd, J = 7.9 Hz, 1H, H3′), 8.45 (td, J = 15.0 Hz, 2H, H5′-H4′), 8.25 (t, J = 8.1 Hz, 1H, H4), 8.06 (dd, J = 7.9 Hz, 1H, H5), 7.80 (m, 1H, H3).

Synthesis of [Pt­(bpy^Cl^-H)­I­(Me)_2_PPh_3_], **7a**. Complex [Pt­(bpy^Cl^-H)­(Me)­PPh_3_] (75.0 mg, 0.114 mmol) and CH_3_I (60.0 μL, 0.6 mmol) were dissolved in CH_2_Cl_2_ and stirred for 72 h under argon at room temperature. The solution was evaporated to dryness using a rotary evaporator, then precipitated and filtered with hexane using a Hirsch funnel to obtain the analytical product, yield 80%. δ (ppm) = 9.60 (d with sat, 1H, *J* = 5.5 Hz, J_Pt–H_ = 11.0 Hz, Hz, H6′); 8.23 (d, 1H, *J* = 7.8 Hz, H3′); 7.85–7.70 (m, 3H, aromatics); 7.30–7.14 (m, 18H, aromatics); 6.97 (dd with sat, 1H, *J* = 8.4, 1.1 Hz, J_Pt–H_ = 43.5 Hz, H4); 6.83 (d with sat, 1H, *J* = 8.4 Hz, J_Pt–H_ = 12.5 Hz H5); 1.69 (d with sat, 3H, *J* = 7.8 Hz, *J*Pt–H = 70.7 Hz, Me); 1.20 (d with sat, 3H, *J* = 7.6 Hz, *J*_Pt–H_ = 60 Hz, Me). ^13^P NMR (CDCl_3_): δ (ppm) = −11.50 (s with sat, *J*_Pt–P_ = 961.2 Hz).

Synthesis of [Pt­(bpy^Br^-H)­I­(Me)_2_PPh_3_] **7b**. Complex [Pt­(bpy^Br^-H)­(Me)­PPh_3_] (54.3 mg, 0.077 mmol) and CH_3_I (60.0 μL, 0.90 mmol) were dissolved in acetone and stirred for 72 h under argon at room temperature. The solution was concentrated using a rotary evaporator, precipitated with hexane, and filtered on a Hirsch funnel to obtain complex **7b** (ivory colored) yield 40%.Melting point: 183–186 °C. ^1^H NMR (CDCl_3_): δ (ppm) = 9.58 (d with sat, 1H, *J* = 5.5 Hz, H6′); 8.20 (d, 1H, *J* = 7.8 Hz, H3′); 7.85–7.66 (m, 4H, aromatics); 7.30–7.19 (m, 8H, aromatics); 7.50–7.35 (m, 10H, meta–para + H5′); 6.93 (d with sat, 1H, *J* = 7.9 Hz, H5); 6.57 (dd with sat, 1H, *J* = 8.3 Hz, H4); 1.65 (d with sat, 3H, *J* = 7.8 Hz, *J*Pt–H = 71.0 Hz, Me); 1.17 (d with sat, 3H, *J* = 7.4 Hz, *J*Pt–H = 53.56 Hz, Me). ^13^P NMR (CDCl_3_): δ (ppm) = −11.56 (s with sat, *J*_Pt–P_ = 956.6 Hz).

Synthesis of [Pt­(bpy^Br^-H)­Br­(PPh_3_)], **8b**

**Method A.** A solution of [Pt­(bpy^Br^-H)­Me­(PPh_3_)] (58.4 mg, 0.083 mmol) and 3,5-di-*tert*-butylbenzyl bromide (96.0 mg, 0.346 mmol) in acetone was stirred for 24 h at 60 °C under an argon atmosphere. After 8 h, an additional portion of 3,5-di-*tert*-butylbenzyl bromide (94.0 mg, 0.332 mmol, MW = 283.25 g/mol) was added. The precipitate was filtered through a Hirsch funnel to yield a first analytical crop (pale yellow). Yield 42%.

**Method B.** A solution of [Pt­(bpy^Br^-H)­Cl­(PPh_3_)] (19.4 mg, 0.026 mmol) and KBr (30.0 mg, 0.25 mmol) in ethanol was stirred at room temperature for 24 h. Then further KBr (85.2 mg, 0.718 mmol) was added, and the mixture was reacted for other 72 h. The solvent was then removed under reduced pressure to yield the product [Pt­(bpy^Br^-H)­Br­(PPh_3_), **8b**, in almost quantitative yield. Mp > 250 °C. ^1^H NMR (CDCl_3_): δ (ppm) = 10.13 (m, 1H, H6′), 8.26 (d, Hz, 1H, H3′), 7.97 (td, Hz, 1H, H4′), 7.80–7.72 (m, 6H, *ortho*), 7.50–7.35 (m, 10H, *meta–para* + H5′), 6.67 (dd with Pt satellites, Hz, 1H, H4), 6.57 (d, Hz, 1H, H5). ^31^P NMR (CDCl_3_): δ (ppm) = 21.76 (s with Pt satellites, J_Pt–P_ = 4252 Hz).

Synthesis of [Pt­(bpy^Br^-H)­(bpy)]­BF_4_ **9b**. Complex [Pt­(bpy^Br^-H)­(DMSO)­(Me)] (65.2 mg, 0.125 mmol), 2,2′-bipyridine (32.6 mg, 0.210 mmol), and [18-crown-6-H_3_O]­[BF_4_] (46.5 mg, 0.125 mmol) were dissolved in CH_2_Cl_2_ and stirred at room temperature under argon for 2 h and 30 min. The orange precipitate formed was filtered to yield complex **9b**, yield 25%. A second sample was obtained from the mother liquor.

^1^H NMR (DMSO-d6): δ (ppm) = 9.14 (d with sat, 1H, *J* = 5.5 Hz, H6⁗); 8.95 (d with sat, 1H, *J* = 5.5 Hz, H6‴); 8.67 (d, 1H, *J* = 6.3 Hz, H6′); 8.62 (m, 2H, H3″ + 1H aromatic); 8.42 (m, 2H, H4″ + 1H aromatic); 8.22 (t, 1H, *J* = 8.0 Hz, H4′); 7.96 (d, 1H, *J* = 7.7 Hz, H3′); 7.88 (t, 1H, *J* = 6.2 Hz); 7.73 (t, 1H, *J* = 6.5 Hz); 7.66 (d, 1H, *J* = 8.3 Hz, H4); 7.58 (ddd, 1H); 7.32 (d, 1H, *J* = 8.3 Hz, H5).

Synthesis of [Pt­(bp^Cl^-H)­(HN = C­(Me)_2_)­Me], **10a**. Complex [Pt­(bpy^Cl^-H)­(DMSO)­Me] (103.7 mg, 0.217 mmol) was dissolved in acetone (20 mL), and 200 μL of a 33% ammonia solution in water was added under vigorous stirring. The mixture was heated to 60 °C % for 12 h under argon. The solution was concentrated to small volume and treated with *n*-hexane. The precipitate formed was filtered under vacuum to give the analytical sample as a dark orange solid (Yield 40%), M.P. = 198–202 °C. ^1^H NMR (CDCl_3_): δ (ppm) = 9.71 (s, 1H, NH); 8.22 (d, 1H, J = 7.7 Hz, H3′); 8.14 (d with sat, 1H, J = 5.5 Hz, H6′); 7.98 (d with sat, 1H, J = 8.0 Hz, H4); 7.85 (td, 1H, J = 8.0 Hz, H4′); 7.15 (ddd, 1H, H5′); 7.06 (d with sat, 1H, J = 8.0 Hz, H5); 2.28 (s, 3H, Imine Me); 2.20 (s, 3H, Imine Me); 0.77 (s with sat, 3H, J_Pt‑Me_ = 84.2 Hz, Me).

Synthesis of [Pt­(bpy^Br^-H)­(HN = C­(Me)_2_)­Me], **10b**. Complex [Pt­(bpy^Br^-H)­(DMSO)­Me] (100.5 mg, M = 522.28 g/mol, 0.192 mmol) was dissolved in acetone, followed by addition of 200 μL of 33% aqueous NH_3_ solution. The mixture was stirred at room temperature for 24 h under argon. After 8 h, an additional 200 μL of NH_3_ solution was added. The solution was concentrated using a rotary evaporator, precipitated with diethyl ether, and filtered on a Hirsch funnel to obtain the first analytical fraction (MM 333, orange, 60.7 mg, M = 503.24 g/mol, 0.121 mmol, yield 65%). The mother liquor was also analyzed, confirming formation of the desired product. Melting point: 159–162 °C. ^1^H NMR (CDCl_3_): δ (ppm) = 9.56 (s, 1H, NH); 8.20 (d, 1H, *J* = 7.6 Hz, H3′); 8.16 (d with sat, 1H, *J* = 5.5 Hz, H6′); 7.87 (m, 2H, H4 + H4′); 7.19 (m, 2H, H5′ + H5); 2.30 (s, 3H, Imine Me); 2.25 (s, 3H, Imine Me); 0.77 (s with sat, 3H, *J*_Pt–H_ = 83.9 Hz, Me).

Synthesis of [Pt­(bpy^Br^-H)­(MeN = C­(Me)_2_)­Me], **11b**. Complex [Pt­(bpy^Br^-H)­(DMSO)­Me] (103.5 mg, 0.198 mmol) was dissolved in acetone, followed by addition of 200 μL of NH_2_Me (41% aqueous solution). The mixture was stirred at room temperature for 24 h under argon, with an additional 200 μL of NH_2_Me added after 8 h. Precipitation with hexane yielded a gummy product which was dried under vacuum to give the analytical product as an orange solid (yield 40%). Melting point: 115–118 °C.^1^ H NMR (CDCl_3_): δ (ppm) = 8.22 (d, 1H, *J* = 8.0 Hz, H3′); 8.07 (d with sat, 1H, *J* = 5.4 Hz, H6′); 7.89 (td, 1H, *J* = 7.6 Hz, H4′); 7.87 (d with sat, 1H, *J* = 7.6 Hz, H4); 7.24 (ddd, 1H, H5′); 7.18 (d, 1H, *J* = 8.0 Hz, H5); 3.56 (dd, 3H, NMe); 2.46 (q, 3H, *J* = 0.8 Hz, Imine Me); 2.16 (s, 3H, Imine Me); 0.77 (s with sat, 3H, *J*Pt-Me = 84.7 Hz, Me). ^13^C NMR spectrum (CDCl_3_) δ (ppm) = 174.97 (C = N), 165.38 (C2 or C2′), 161.82 (C2 or C2′), 145,76 (CH), 143.74, 143.57, 138.77 (Cq), 137.57 (CH), 135.63 (Cq), 127.89 (CH), 124.55 (CH), 121.62 (CH), 43.19 (N–CH_3_, J_Pt–C_ = 25.0 Hz), 29.85 (C–CH_3_), 32.71 (C–CH_3_), −18.62 (CH_3_–Pt, J_Pt–C_ = 809.0 Hz).

## Supplementary Material







## Footnotes

1 The definition and calculation method for the ζ angle were reported in the Supporting Information of ref 43.

## Notes

The Supporting Information is available free of charge at https://pubs.acs.org/doi/10.1021/acsomega.6c05687. Deposition Numbers CCDC 2548065 and 2548066 contain the supplementary crystallographic data for this paper. These data can be obtained free of charge via the joint Cambridge Crystallographic Data Centre (CCDC)

(CIF)
(CIF
Table S1: selected crystallographic data for complexes **3b** and **6b**; Figures S1–S58: NMR spectra of the new complexes; Figures S59–S62: CV responses (PDF)
