# Supplementary material for: Pt(II) Rollover Complexes with Poor-Donor Bipyridines: Synthesis, Reactivity, and Preliminary Antimicrobial Evaluation
Source: ACS Omega. 2026 Jul 30;11(31):47011–31. doi: 10.1021/acsomega.6c05687 (PMC13470765; doi:10.1021/acsomega.6c05687)
Supplement: Supplementary file 1 [file ao6c05687_si_003.pdf]

Supporting Information

Pt(II) Rollover complexes with poor-donor bipyridines: synthesis, reactivity and preliminary antimicrobial evaluation

Antonio Zucca,*[a,b] Matteo Murineddu,[a] Fabrizio Ortu,[c] Yu Liu,[c] Gabriele Manca,[d] Germano Orrù,[e] Alessandra Scano,*[e] and Maria I. Pilo[a]

a) University of Sassari, Department of Chemical, Physical, Mathematical and Natural Sciences, via Vienna 2, I-07100 Sassari, Italy
b) Consorzio Interuniversitario Reattività Chimica e Catalisi (CIRCC) 70126 Bari, Italy
c) School of Chemistry, University of Leicester, University Road. Leicester, LE1 7RH (UK)
d) Istituto di Chimica dei Composti Organometallici (ICCOM)-Sede Secondaria di Bari, Consiglio Nazionale delle Ricerche (CNR), c/o Università degli Studi di Bari "Aldo Moro", Via Orabona 4, 70126 Bari, Italy
e) Department of Surgical Sciences, University of Cagliari, Cagliari 09124, Italy

email: zucca@uniss.it (AZ); alessandra.scano77@unica.it (AS)

Table of Contents

**Page 2**	**Table S1**: Selected crystallographic data for complexes **3a** **and 6b**
**Pages 3–5**	Figures S1-S6: NMR spectra of complex **1a**
**Page 6–10**	Figures S7-S15: NMR spectra of complex **1b**
**Pages 11–-12**	Figures S6-S18: NMR spectra of complex **2a**
**Pages 13–14**	Figures S19-S21: NMR spectra of complex **2b**
**Page 15**	Figures S22-S23: NMR spectra of complex **3a**
**Pages 16–17**	Figures S24-S25: NMR spectra of complex **3b**
**Pages 18–19**	Figures S26-S28: NMR spectra of complex **4a**
**Pages 19-20**	Figures S29-S31: NMR spectra of complex **4b**
**Pages 21–22**	Figures S32-S34: NMR spectra of complex **5b**
**Page 23**	Figures S35-S36: NMR spectra of complex **6a**
**Page 24**	Figures S37-S38: NMR spectra of complex **6b**
**Page 25**	Figures S39-S40: NMR spectra of complex **7a**
**Pages 26-27**	Figures S41-S43: NMR spectra of complex **8b**
**Pages 28-29**	Figures S44-S46: NMR spectra of complex **9b**
**Page 30**	Figures S47-S48: NMR spectra of complex **10a**
**Page 31**	Figures S49-50: NMR spectra of complex **10b**
**Pages 32-33**	Figures S51-S54: NMR spectra of complex **11b**
**Pages 34-35**	Figures S55-58: NMR spectra of **1a** and **1b** in CD_3CN
**Pages 36-37**	Figures S59-S62: CV responses
**Page 37**	Scheme S1: Sequence of reactions proposed in the formation of 3-methyl-6-chloro-2,2'-bipyridine and 3-methyl-6-bromo-2,2'-bipyridine.
**Pages 38-44**	Cartesian coordinates and free energies of all the structures optimized in the computational analysis

Table S1. Selected crystallographic data for complexes **3b** and **6b**.

	**3b**	**6b**
CCDC No.	2548066	2548065
formula	$C_{12}H_{12}BrClN_2OPtS$	$C_{10}H_7BrCl_2N_2Pt$
Mr	542.73	501.06
Crystal system	monoclinic	monoclininc
Space group	$P2_1/n$	$P2_1/n$
a, Å	8.9315(3)	7.1680(3)
b, Å	16.2251(6)	10.2212(5)
c, Å	10.5350(4)	16.2355(7)
α, deg.	90	90
β, deg.	110.214(2)	97.099(2)
γ, deg.	90	90
V, Å^3	1432.64(9)	1180.38(9)
Z	4	4
$\rho_{calc,}$ g/cm^3	2.516	2.820
μ, mm^{-1}	24.611	30.136
F(000)	1008	912
No. reflections (unique)	22114(2753)	10268(2305)
S[a]	1.17	1.09
$R_1(wR_2)$ ($F^2 > 2\sigma(F^2)$)	0.0892, 0.2239	0.551, 0.1485
R_{int}	0.048	0.046
Min./max diff map e Å^{-3}	–2.40, 5.51	–1.94, 4.47

[a]Conventional $R = \Sigma||Fo| - |Fc||/\Sigma|Fo|$; $Rw = [\Sigma w(Fo^2 - Fc^2)^2/\Sigma w(Fo^2)^2]^{1/2}$; $S = [\Sigma w(Fo^2 - Fc^2)^2/\text{no. data} - \text{no. params})]^{1/2}$ for all data.

NMR spectra

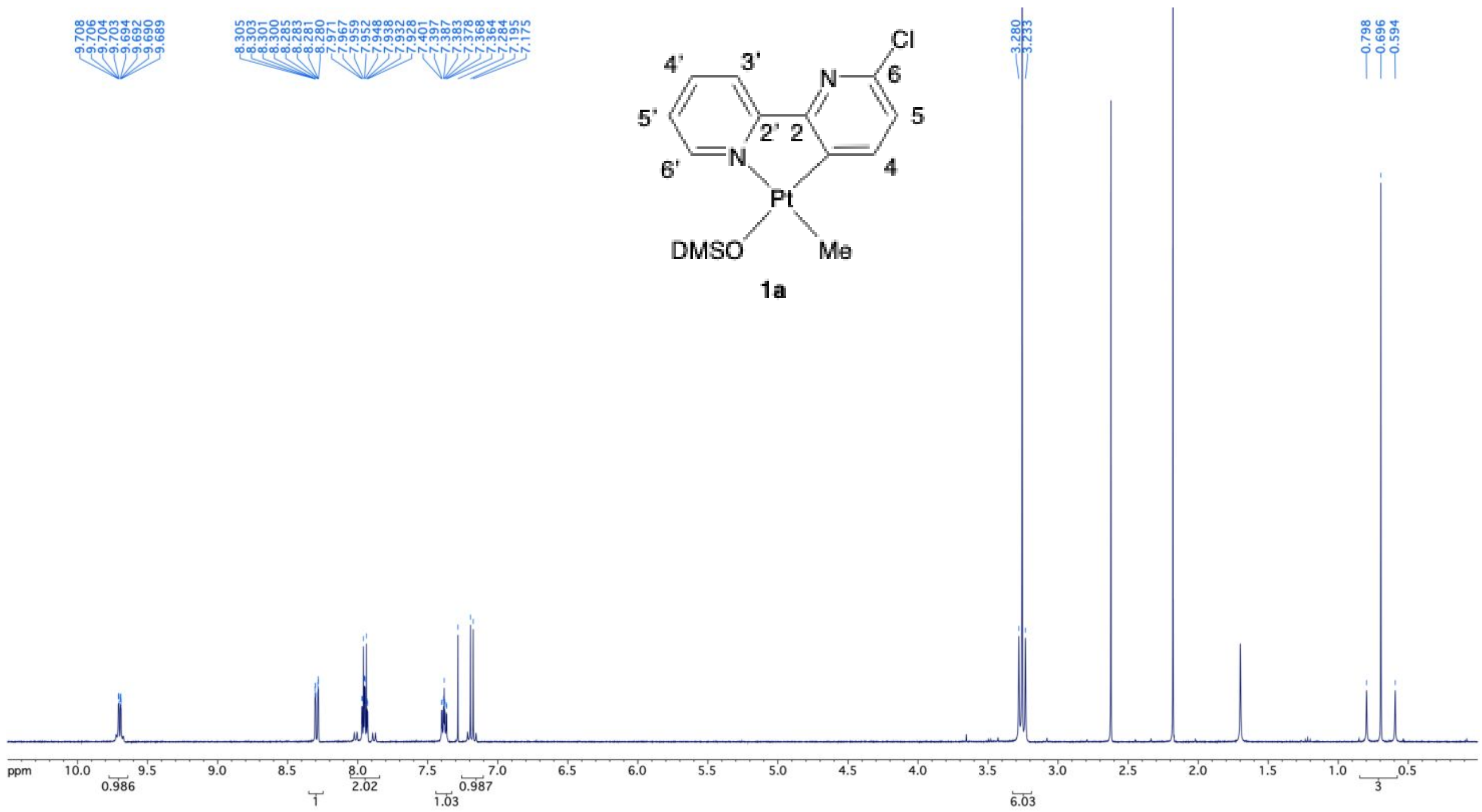

Figure S1. ^{1}H NMR spectrum ($CDCl_3$) of complex **1a**

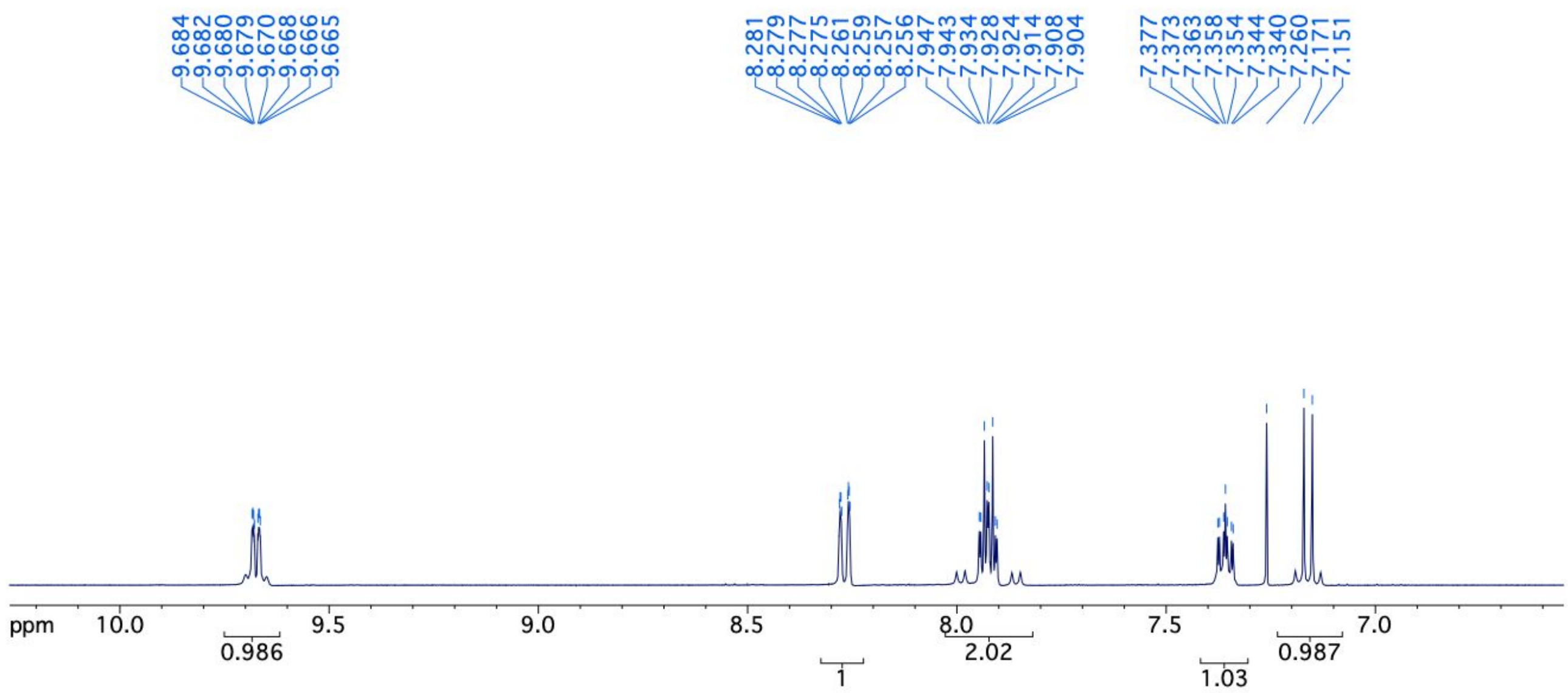

Figure S2. ^{1}H NMR spectrum (aromatic region, $CDCl_3$) of complex **1a**

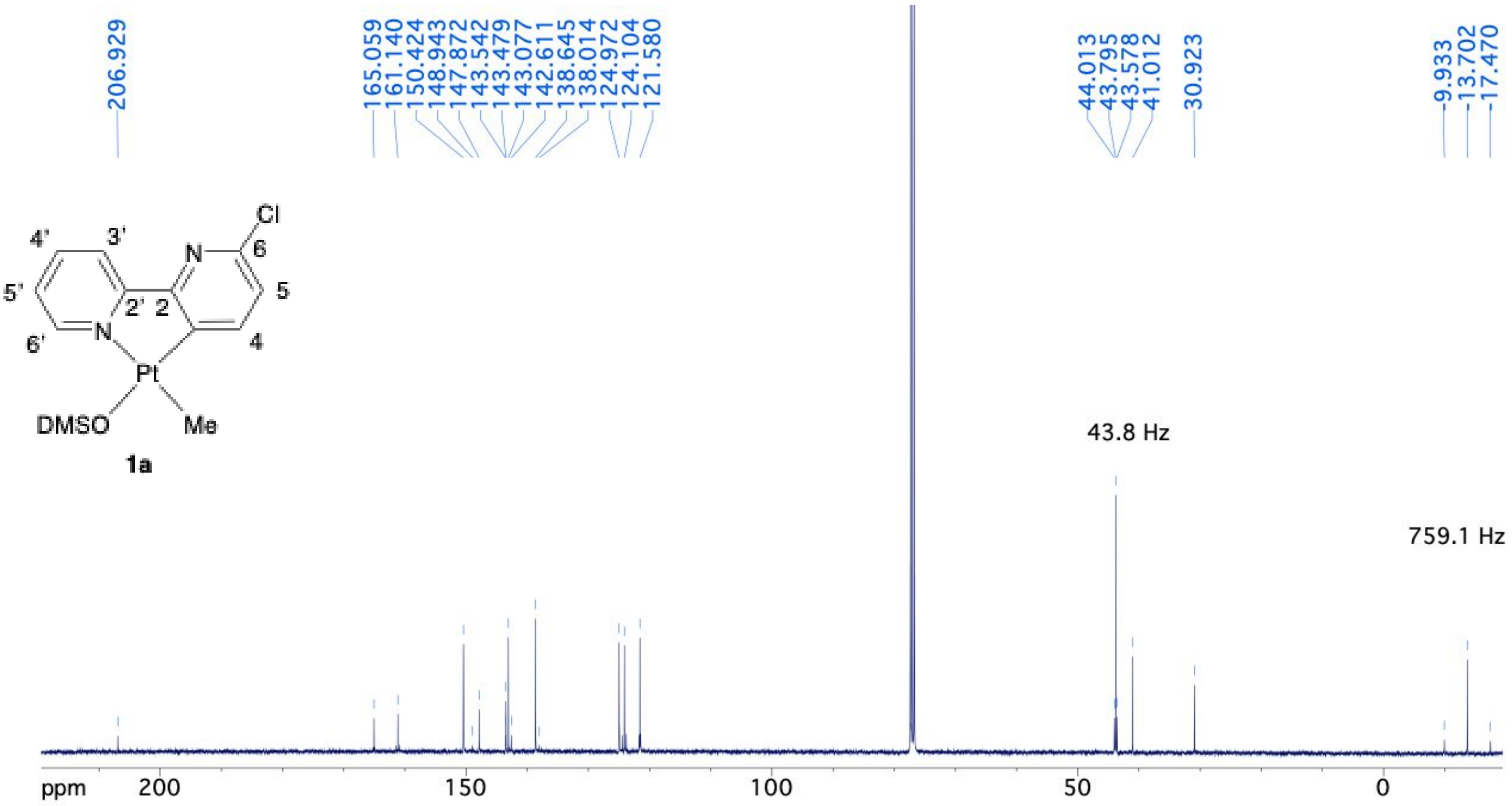

Figure S3. ^{13}C NMR spectrum (acetone-d6) of **1a**

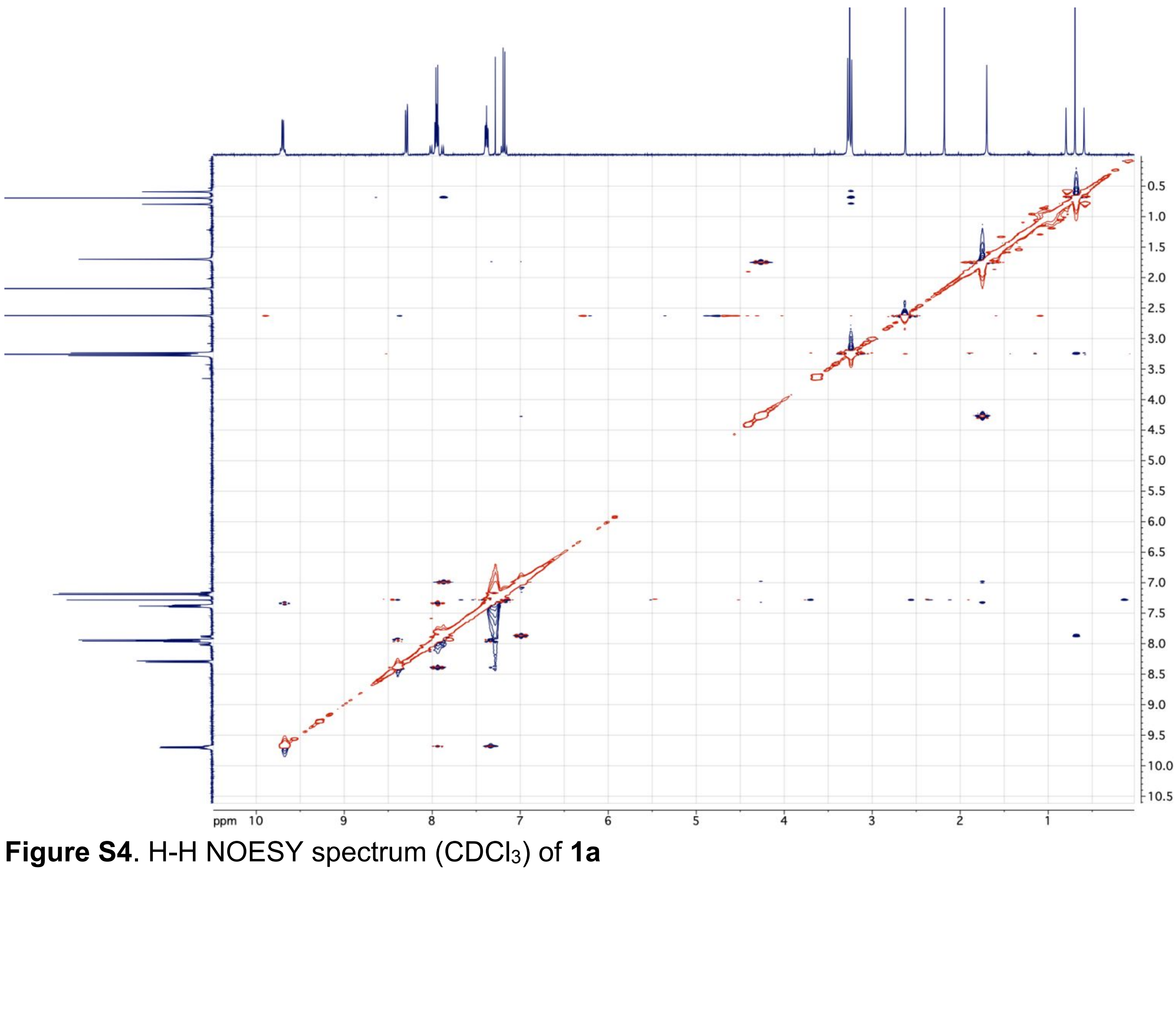

Figure S4. H-H NOESY spectrum ($CDCl_3$) of **1a**

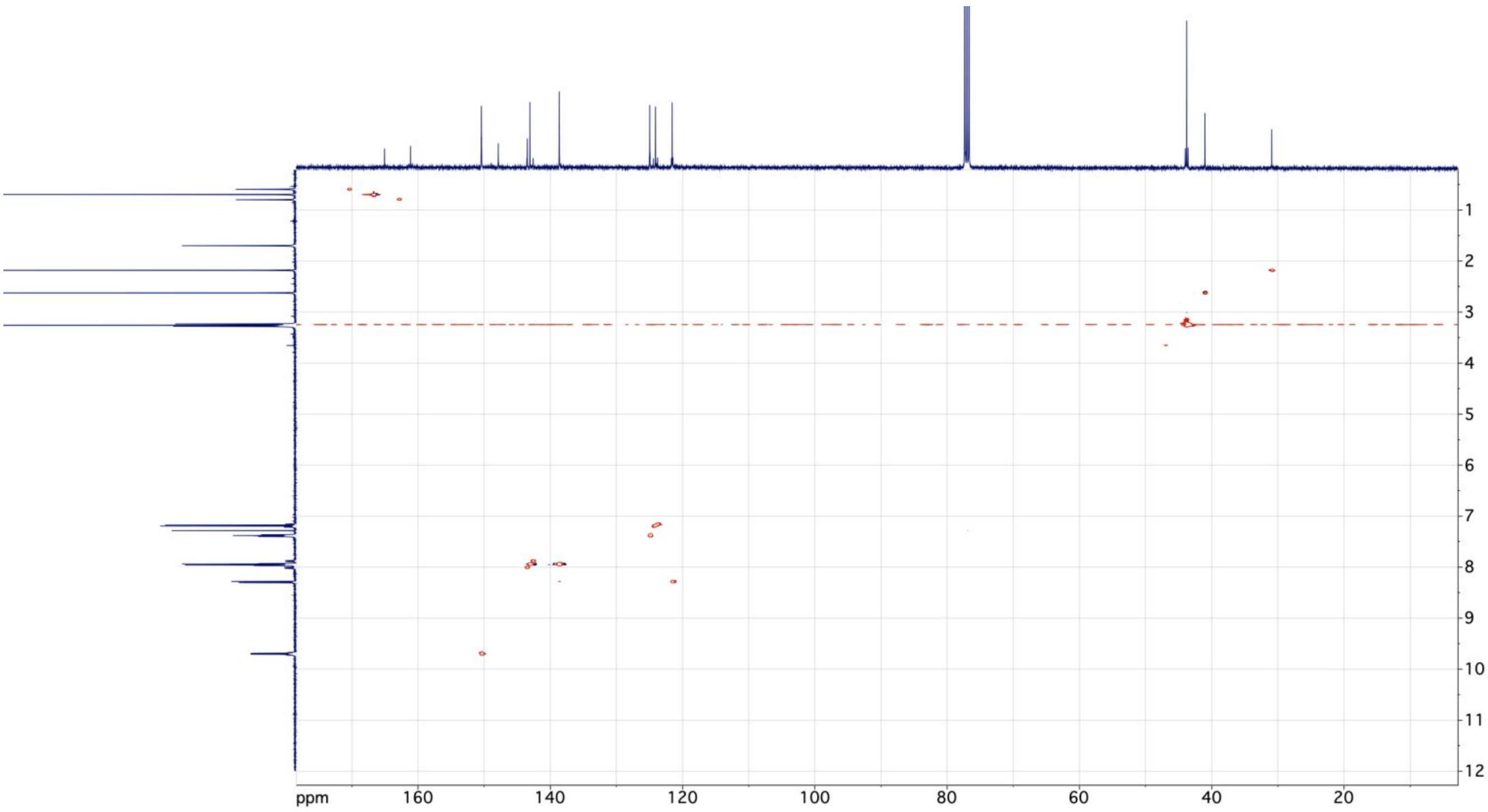

Figure S5. H-C HSQC spectrum ($CDCl_3$) of **1a**

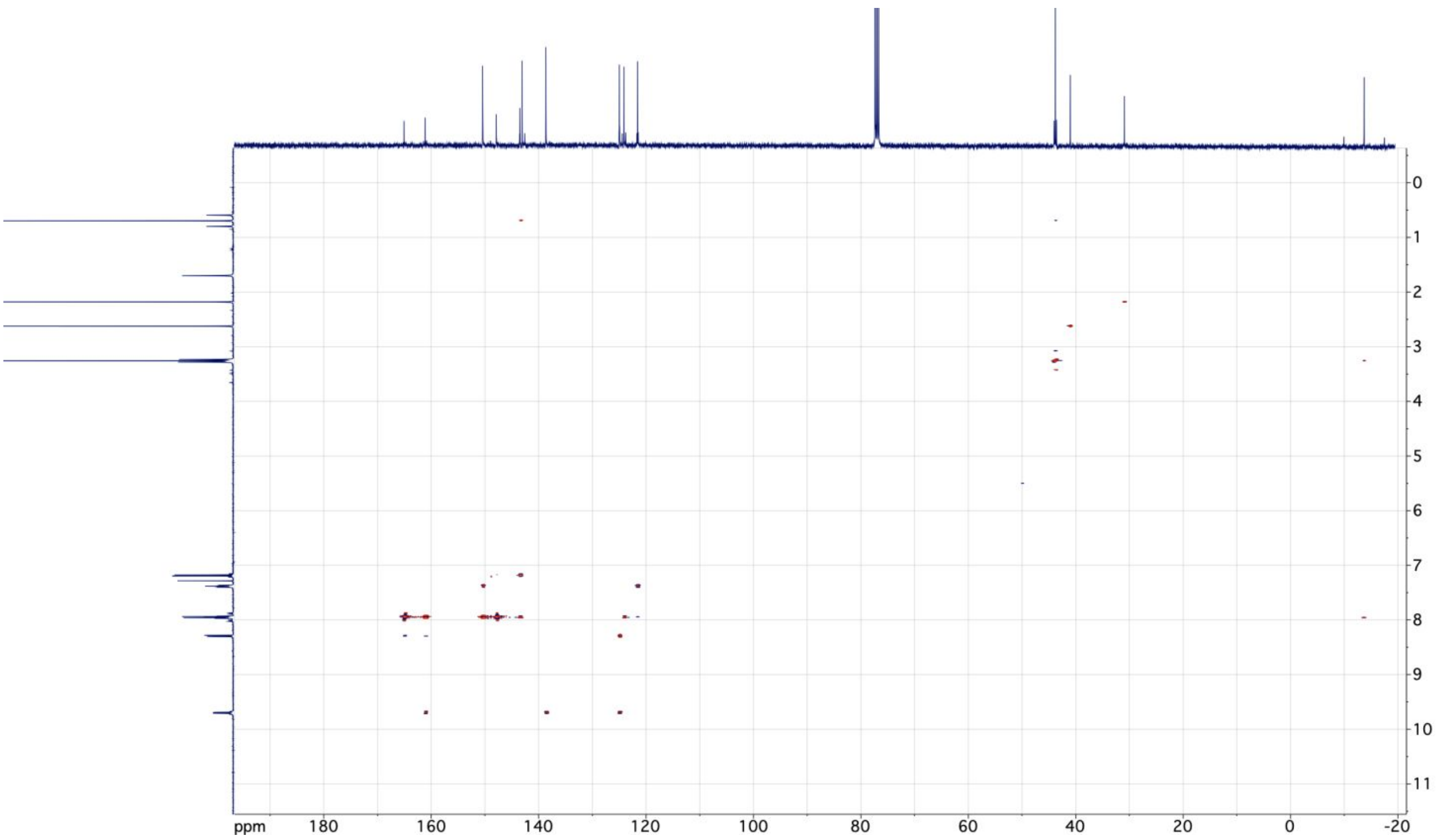

Figure S6. H-C HMBC spectrum ($CDCl_3$) of **1a**

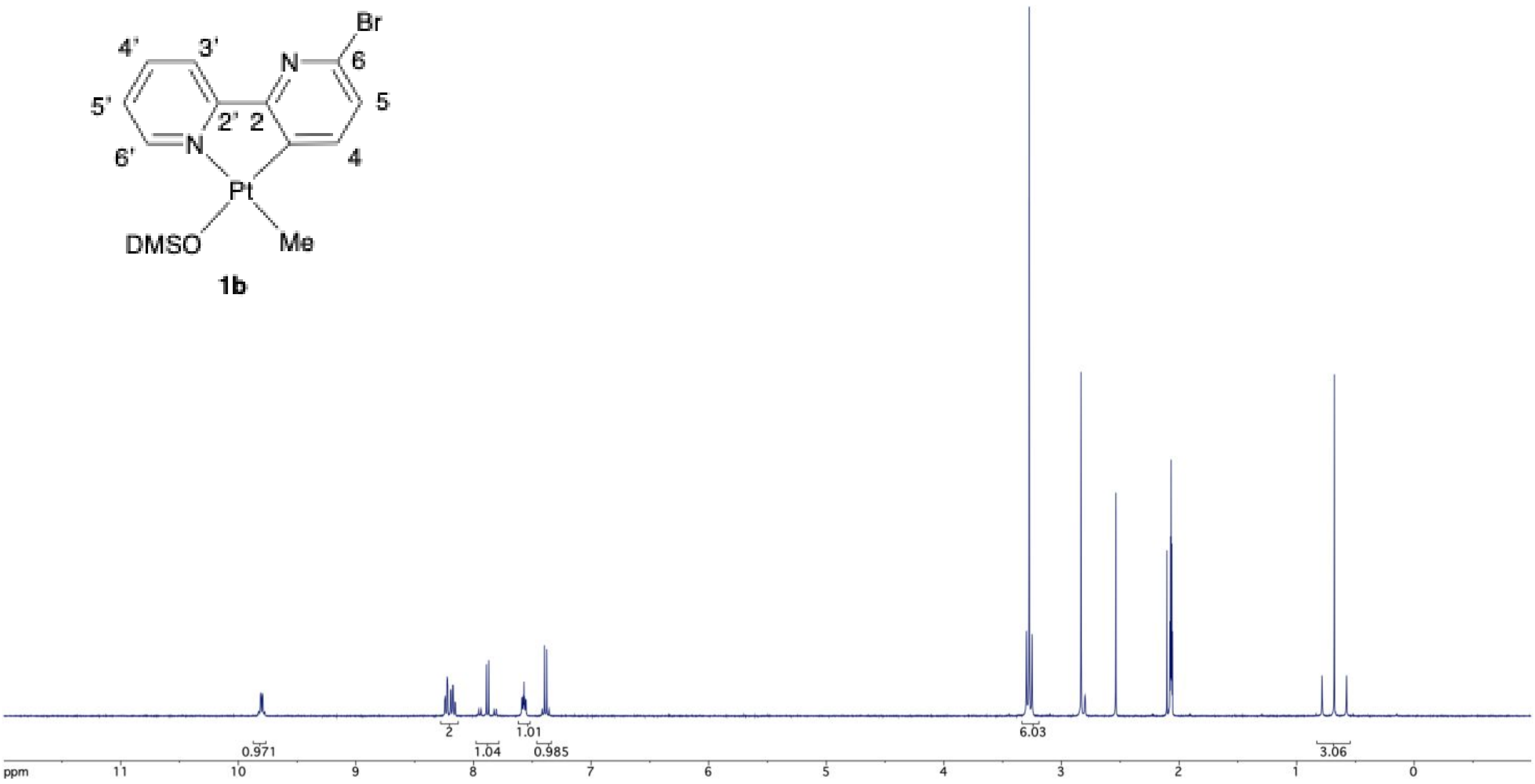

Figure S7. ^{1}H NMR spectrum (acetone-d_6) of **1b**

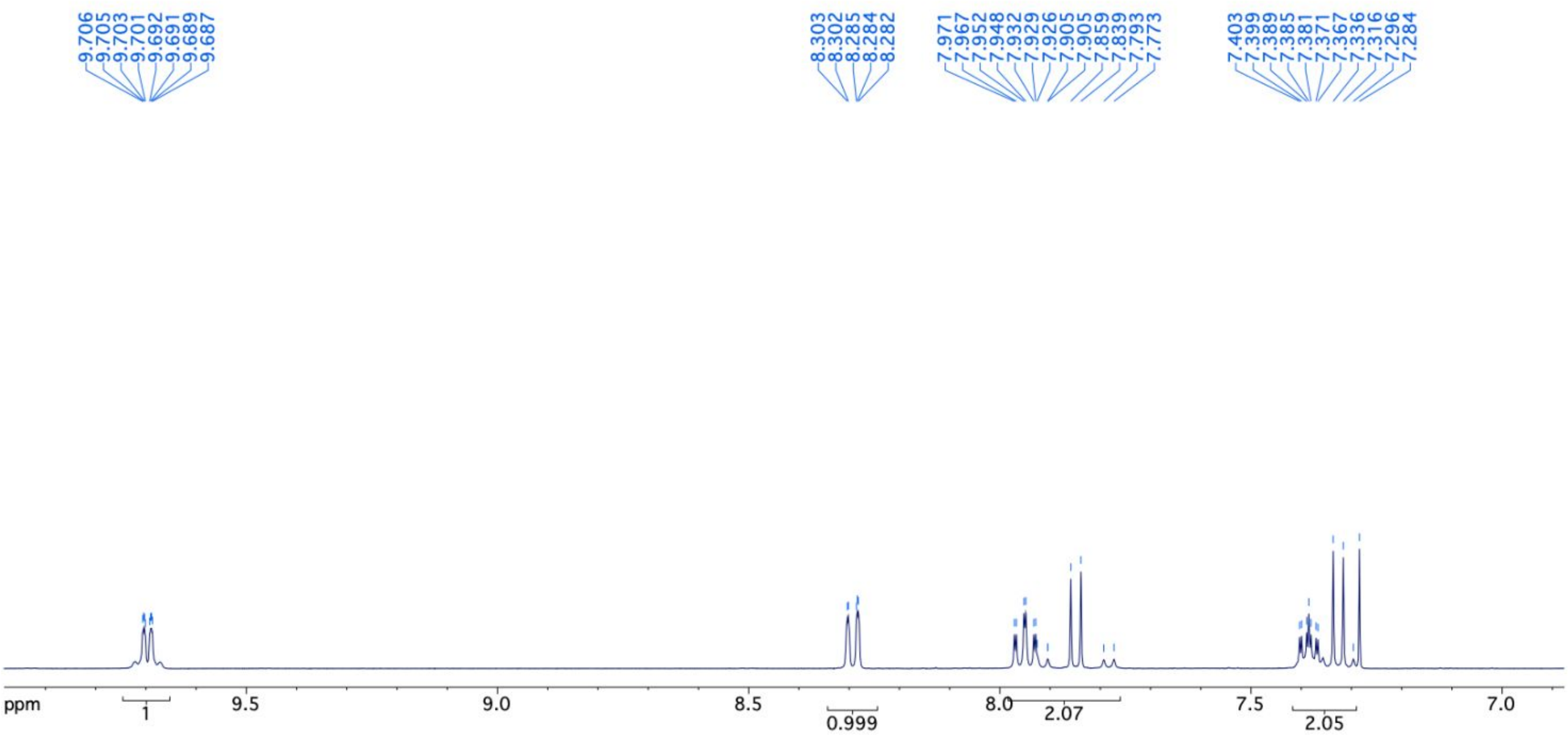

Figure S8. ^{1}H NMR spectrum (aromatic region, acetone-d_6) of **1b**

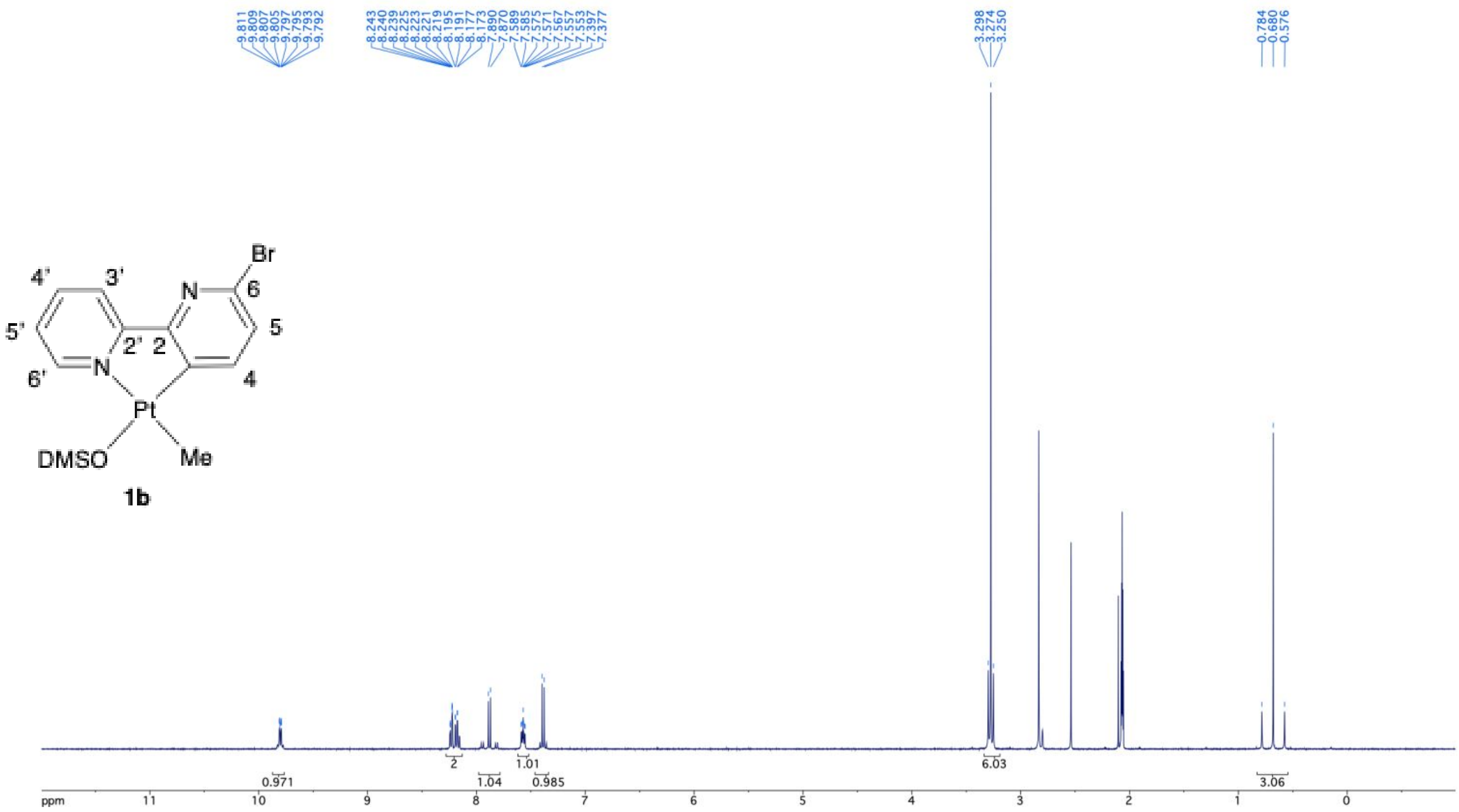

Figure S9. ^{1}H NMR spectrum (acetone-d6) of **1b**

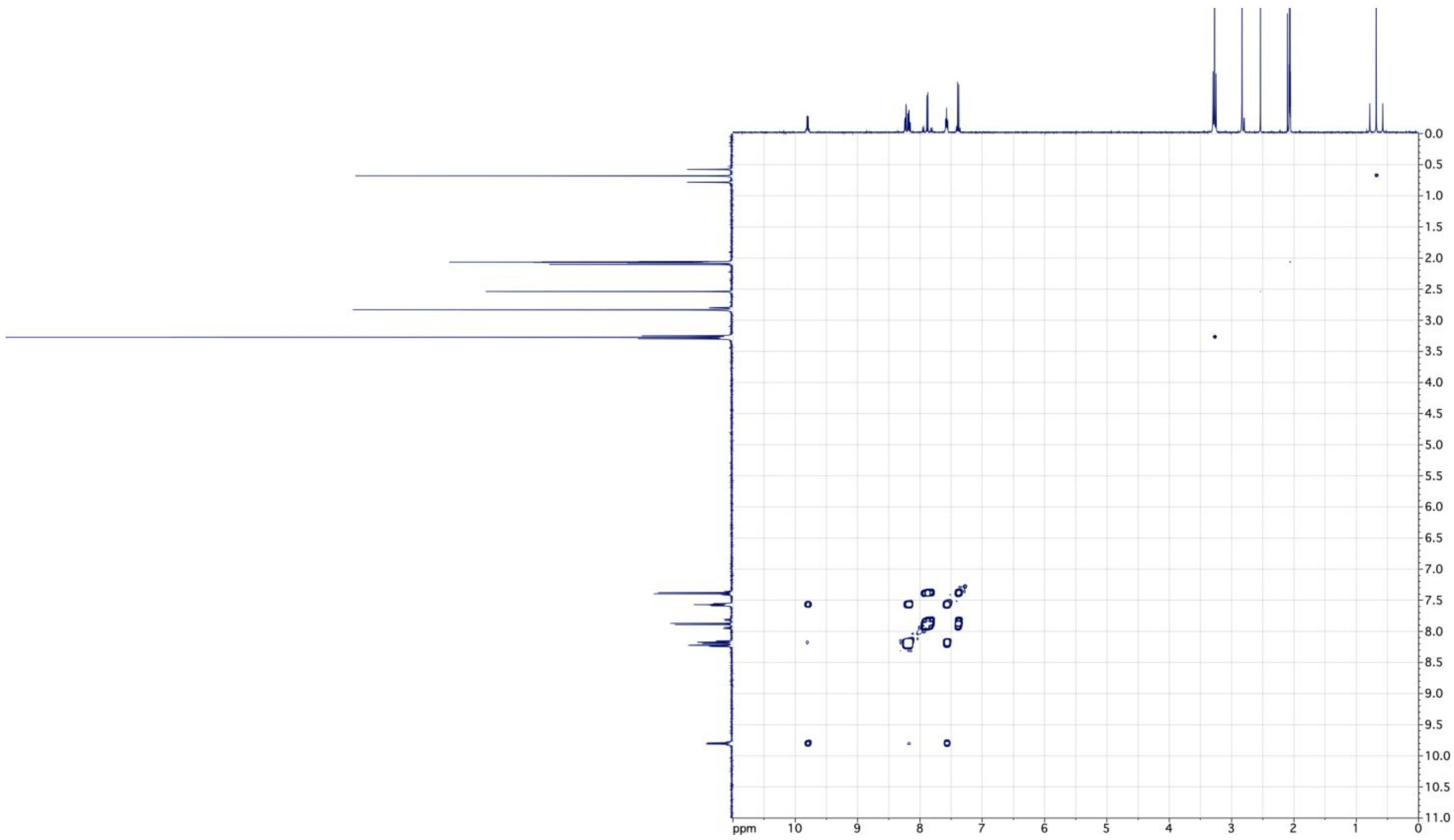

Figure S10. ^{1}H NMR COSY spectrum (acetone-d6) of **1b**

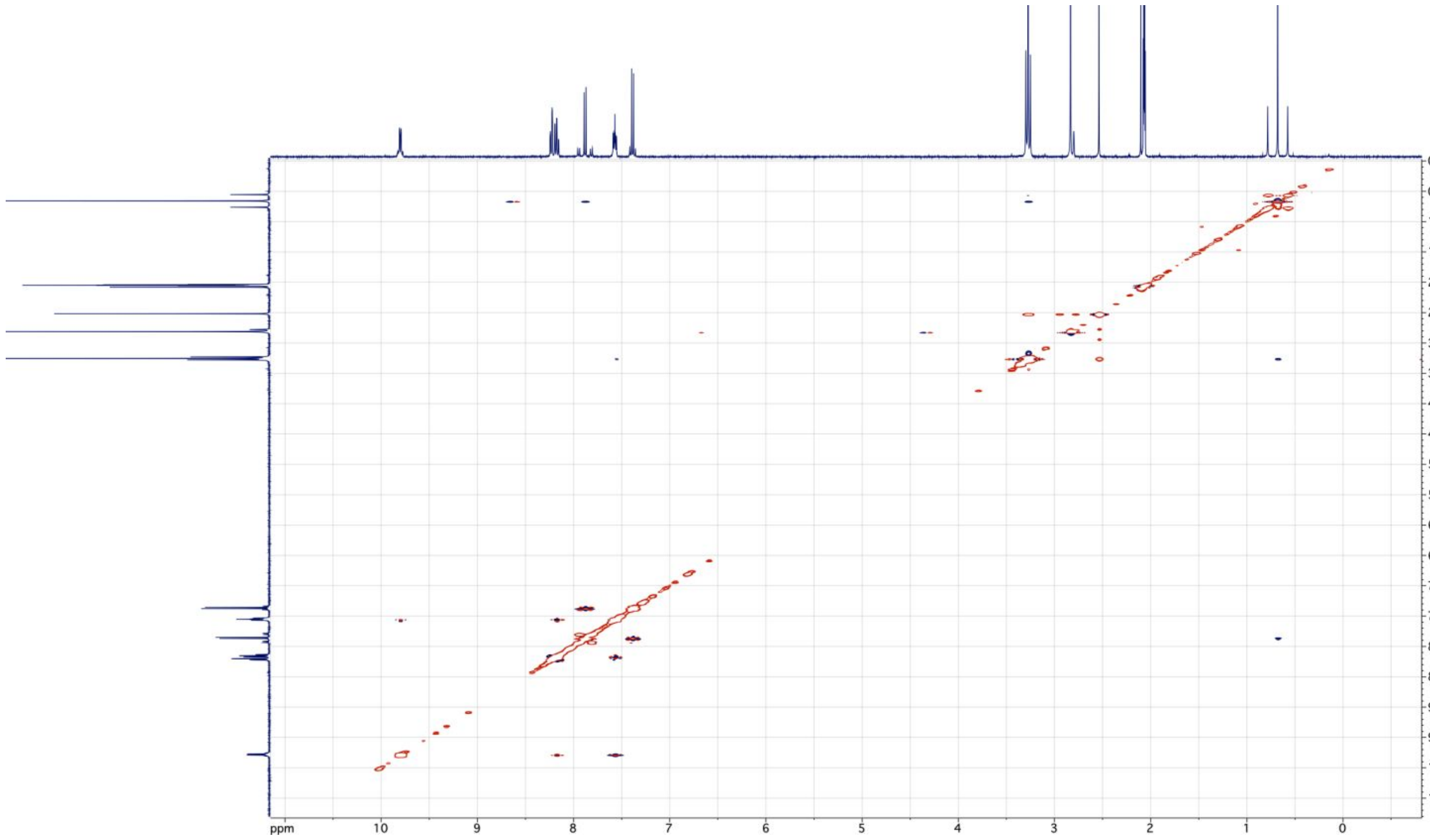

Figure S11. ^{1}H NMR NOESY spectrum (acetone-d6) of **1b**

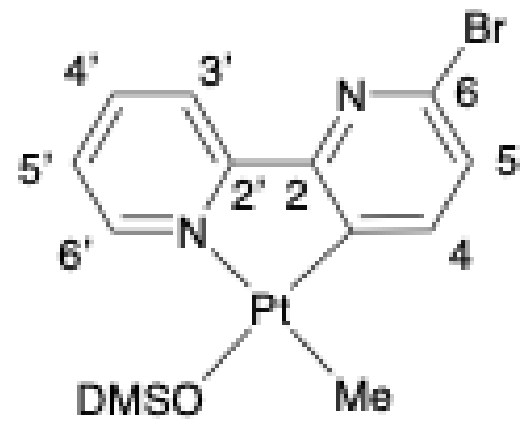

1b

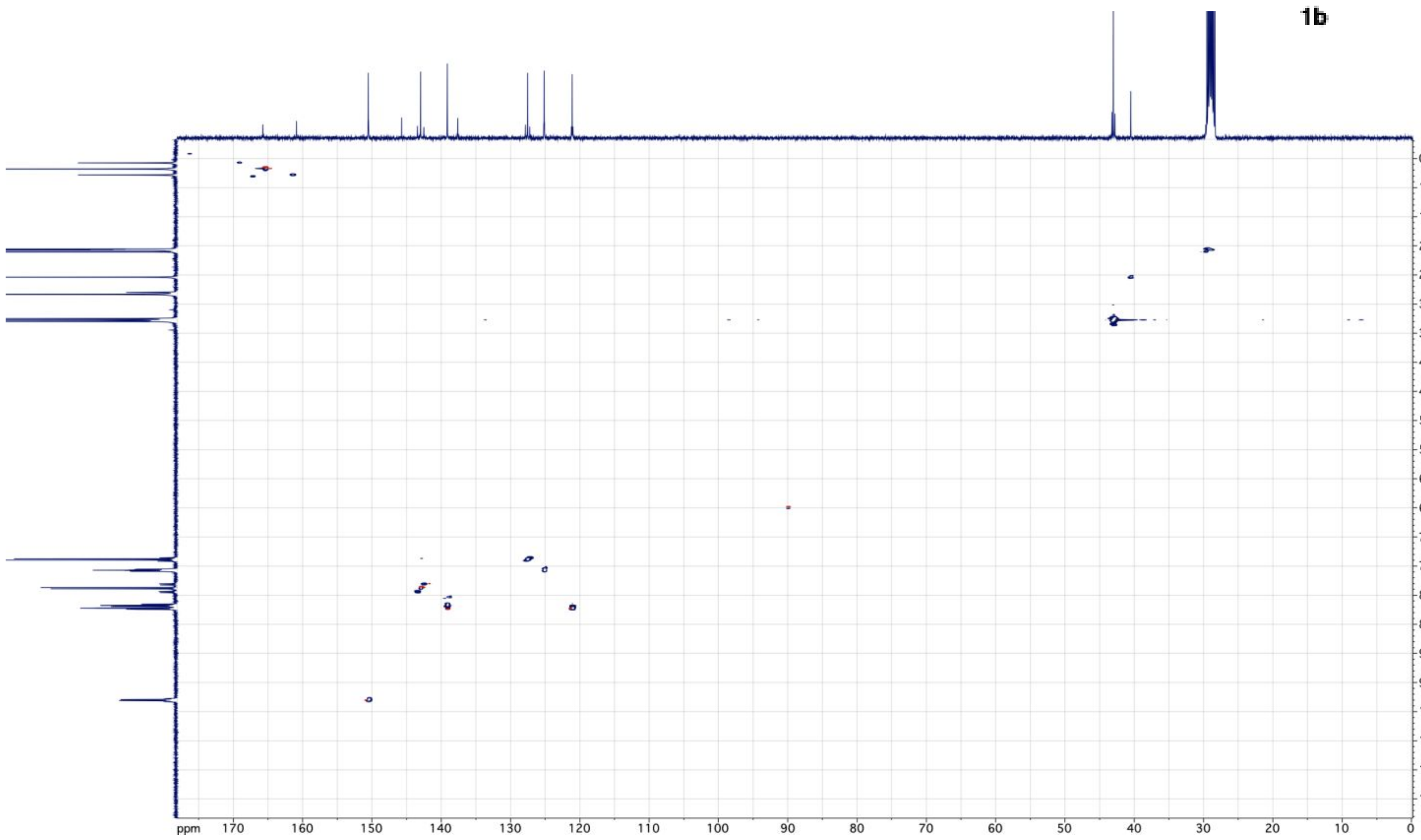

Figure S12. H-C HSQC NMR spectrum of **1b** (the methyl cross peaks appear as refolding signals due to a narrow ^{13}C spectral window)

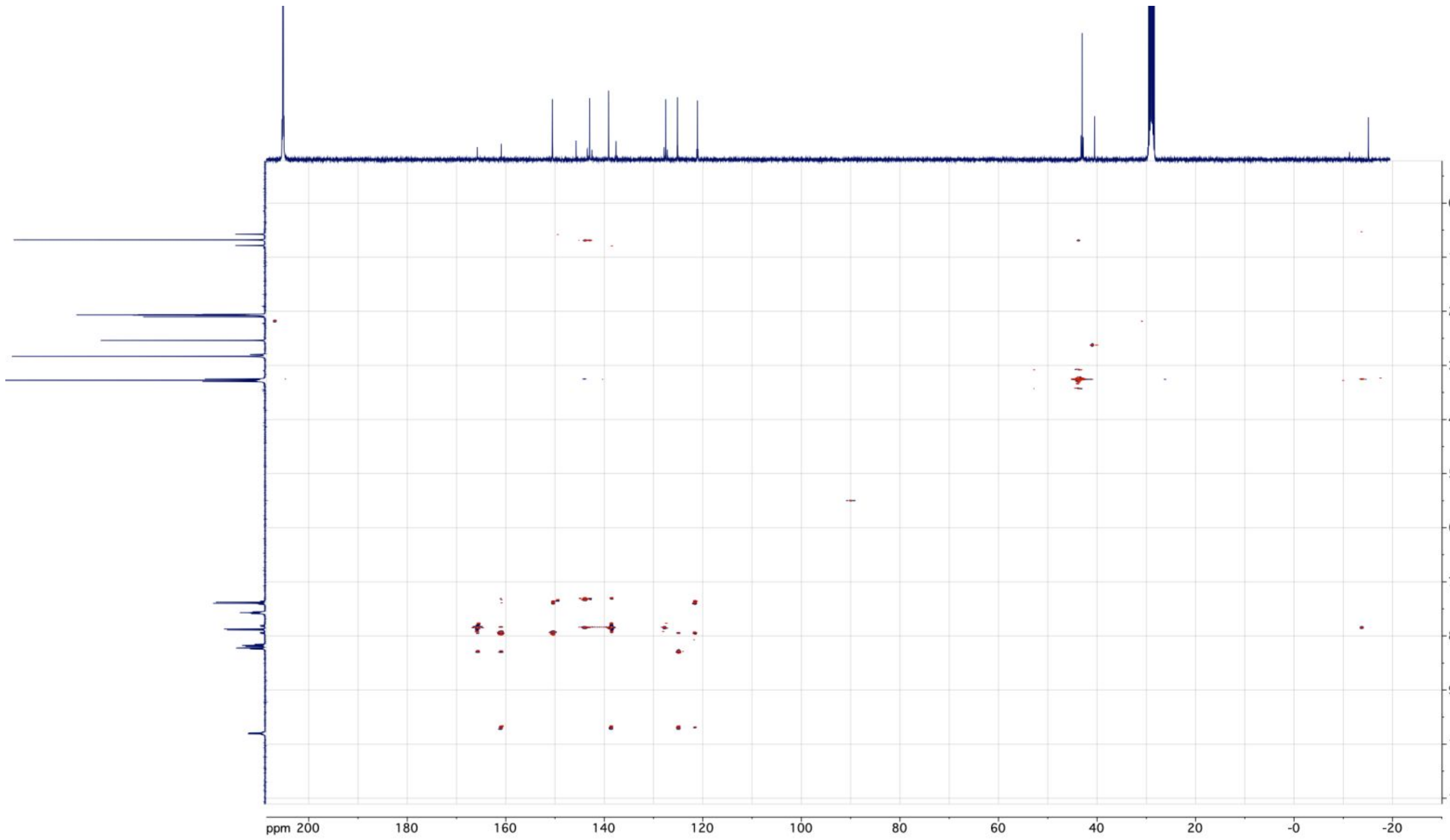

Figure S13. H-C HMBC NMR spectrum (acetone-d6) of **1b**

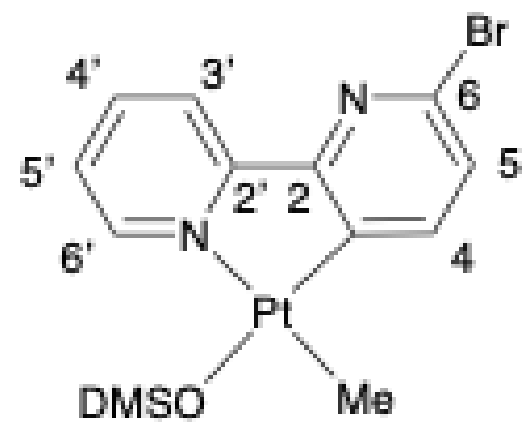

1b

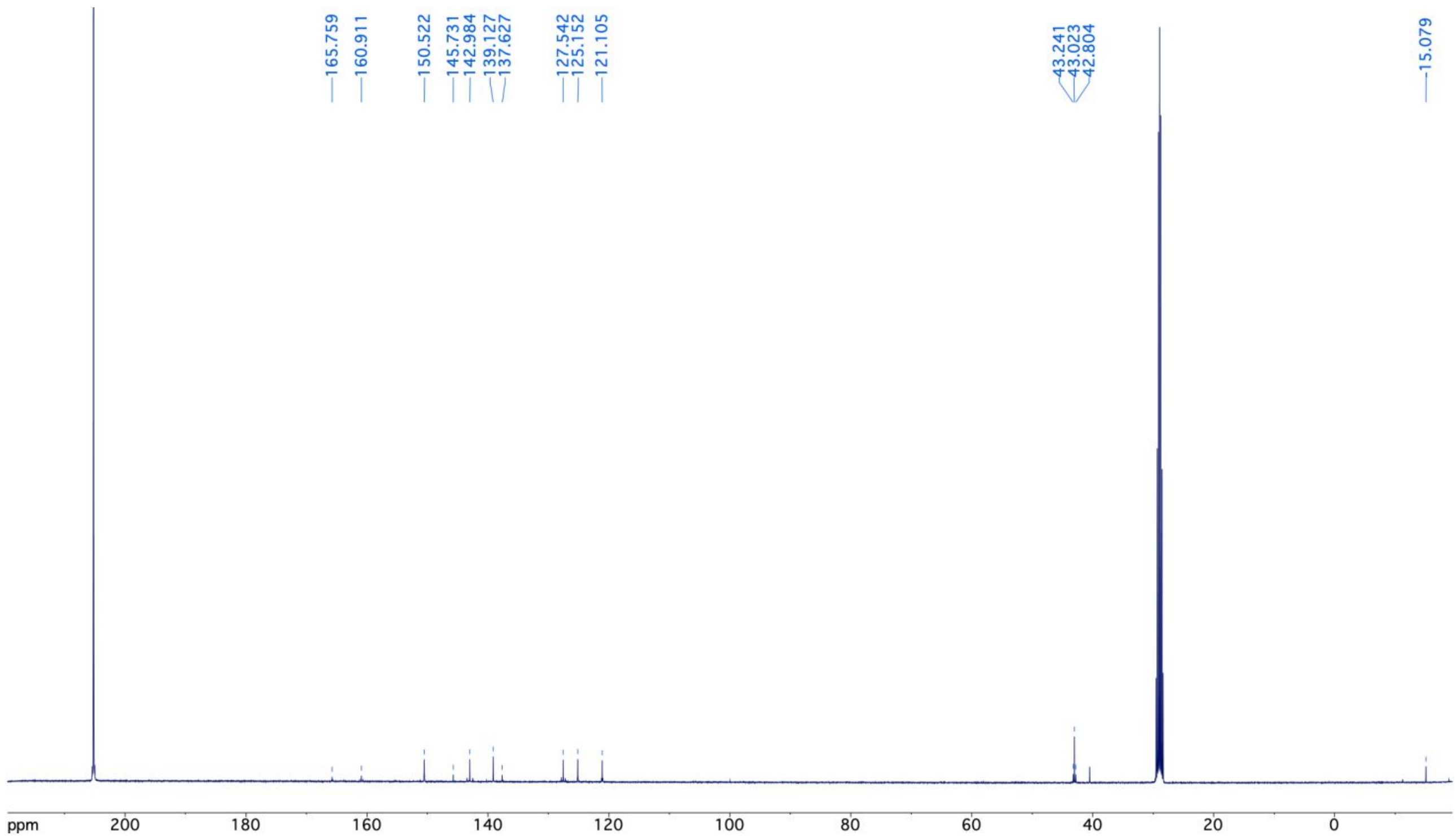

Figure S14. ^{13}C NMR spectrum of **1b** (acetone -d6)

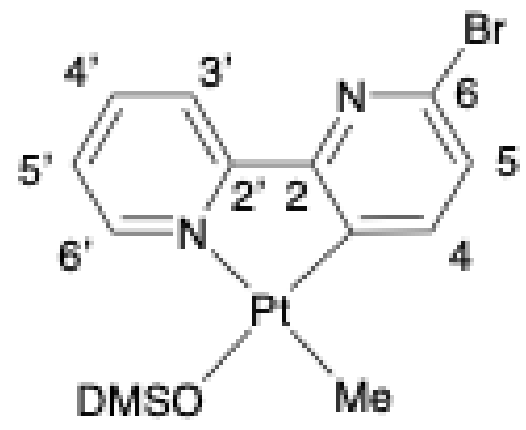

1b

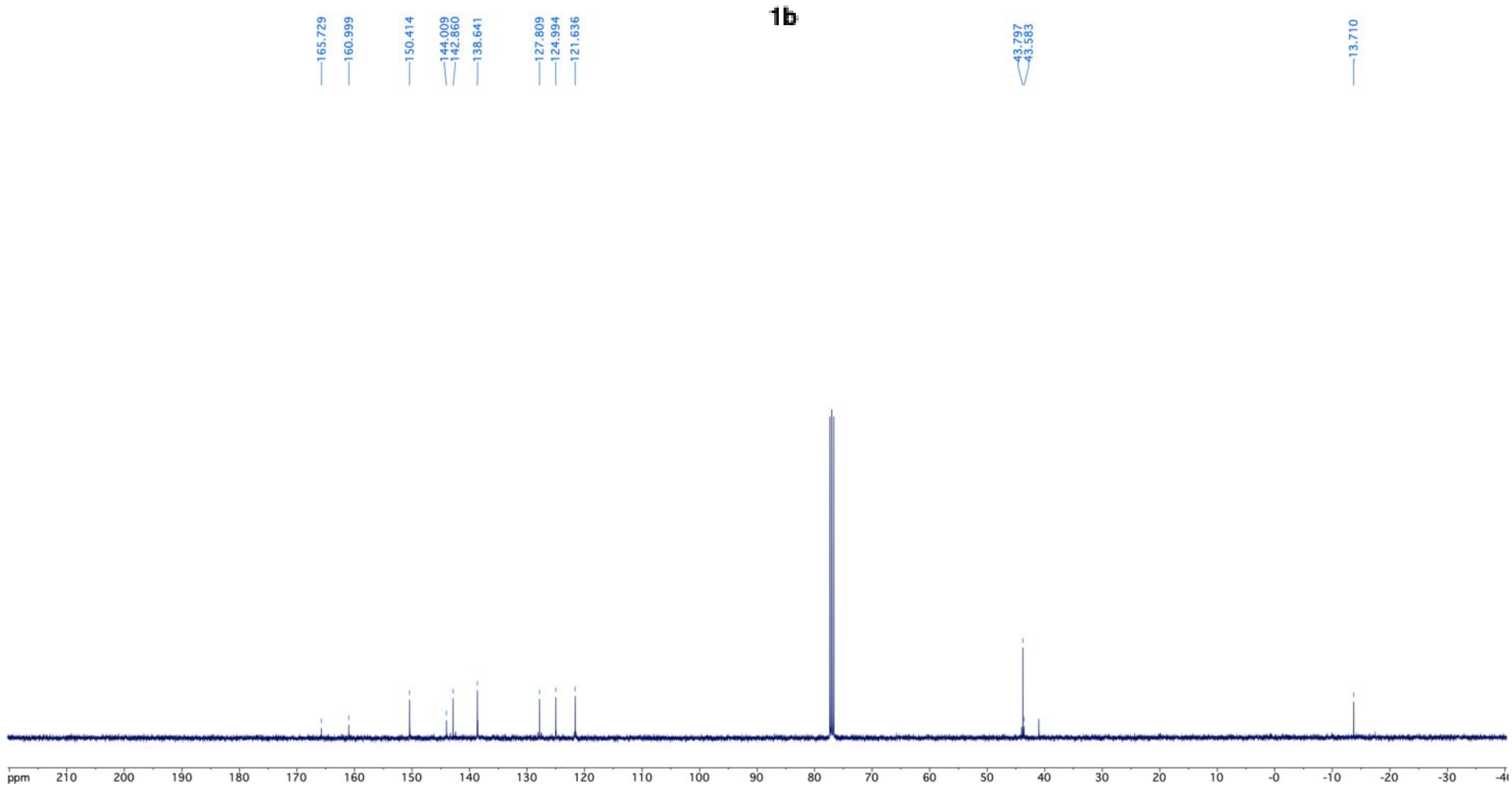

Figure S15. ^{13}C NMR spectrum ($CDCl_3$) of **1b**.

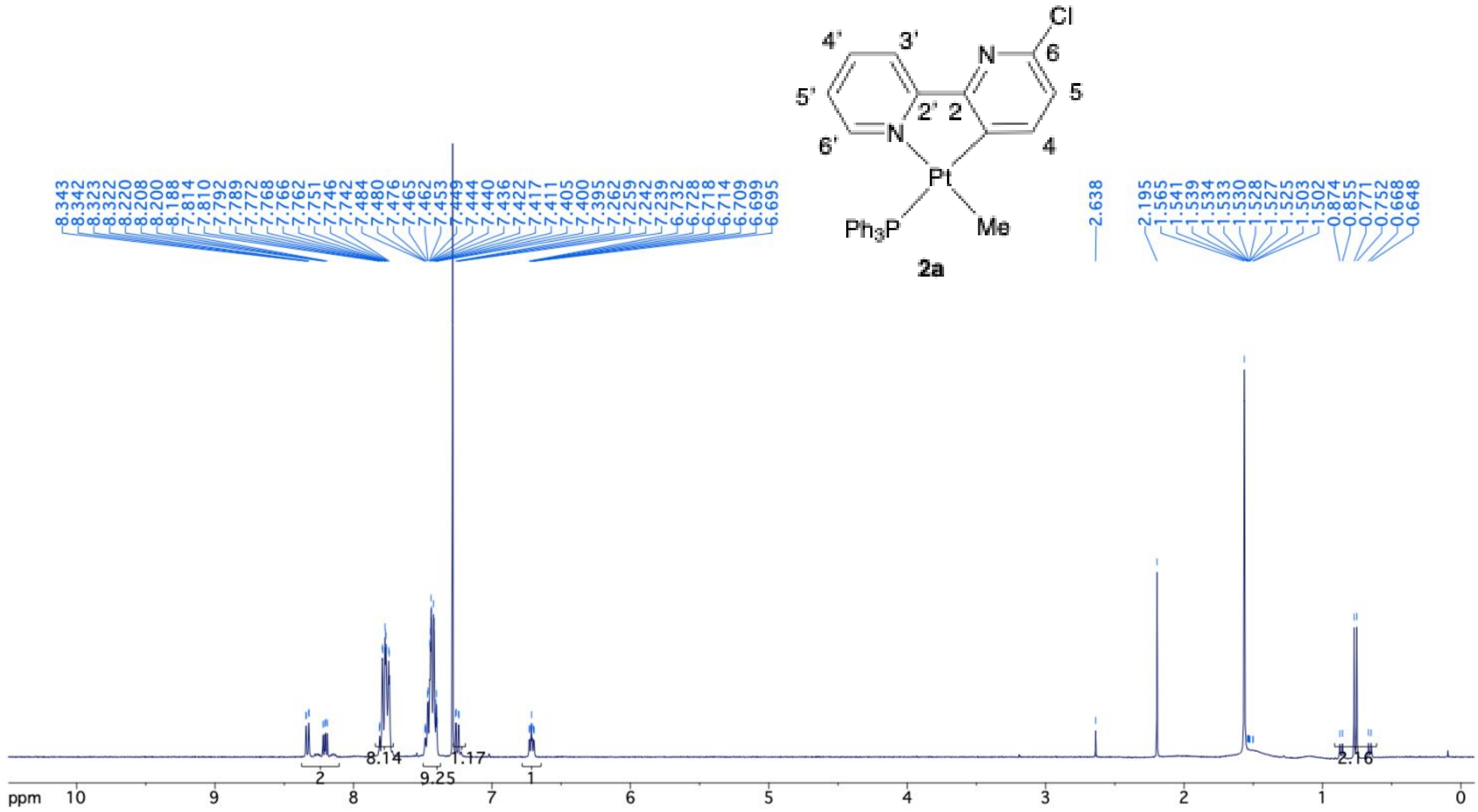

Figure S16. ^{1}H NMR spectrum ($CDCl_3$) of **2a**.

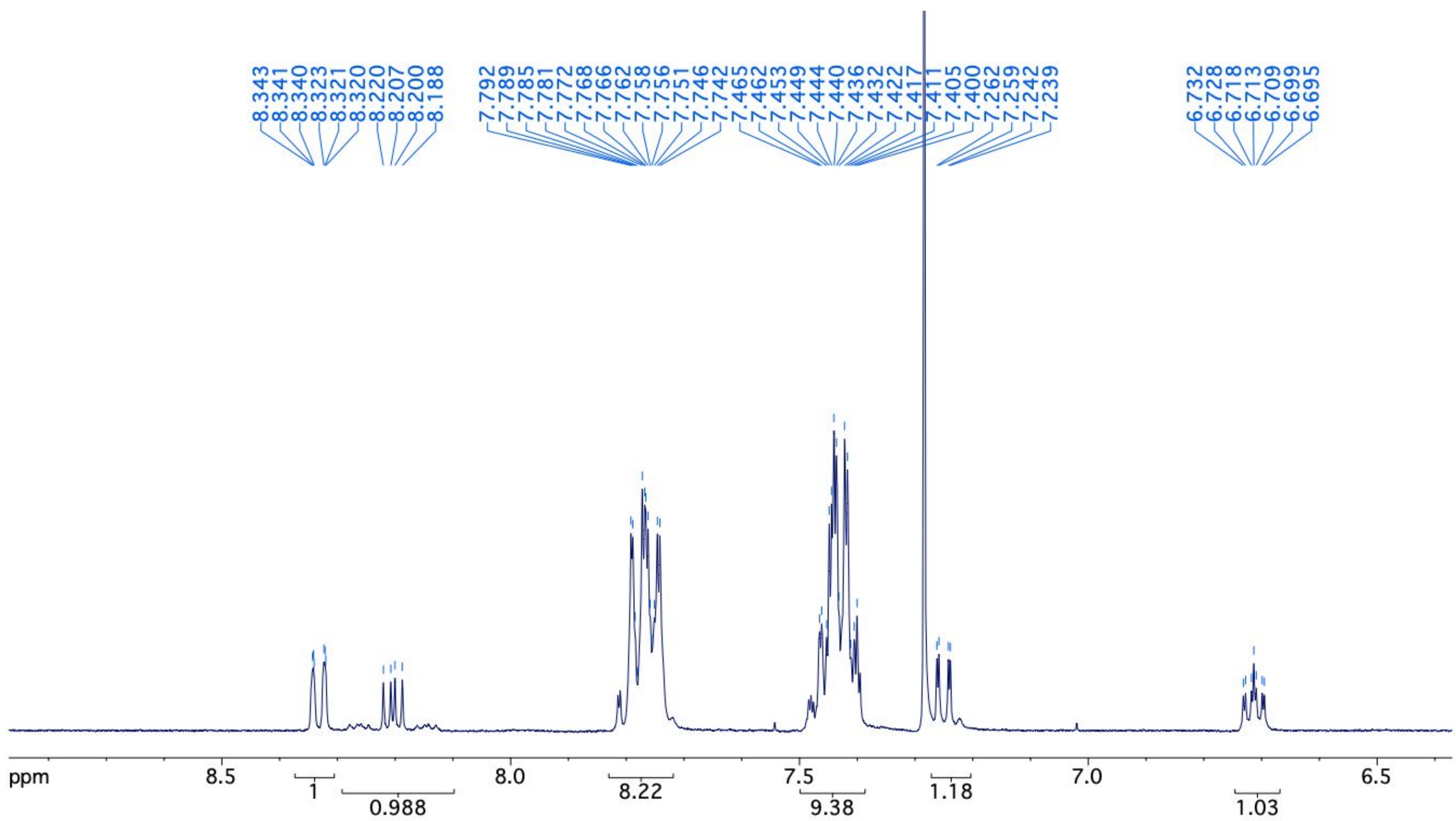

Figure S17. ^{1}H NMR spectrum (aromatic region, $CDCl_3$) of **2a**.

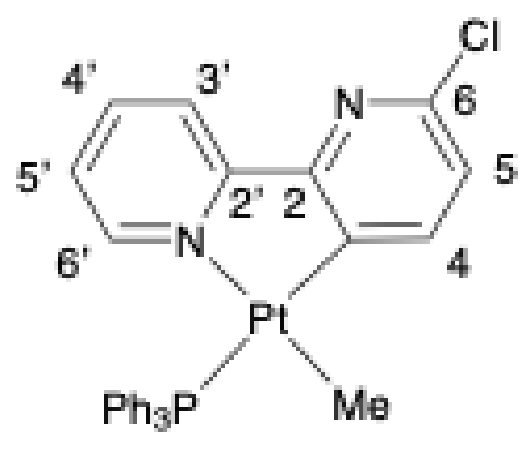

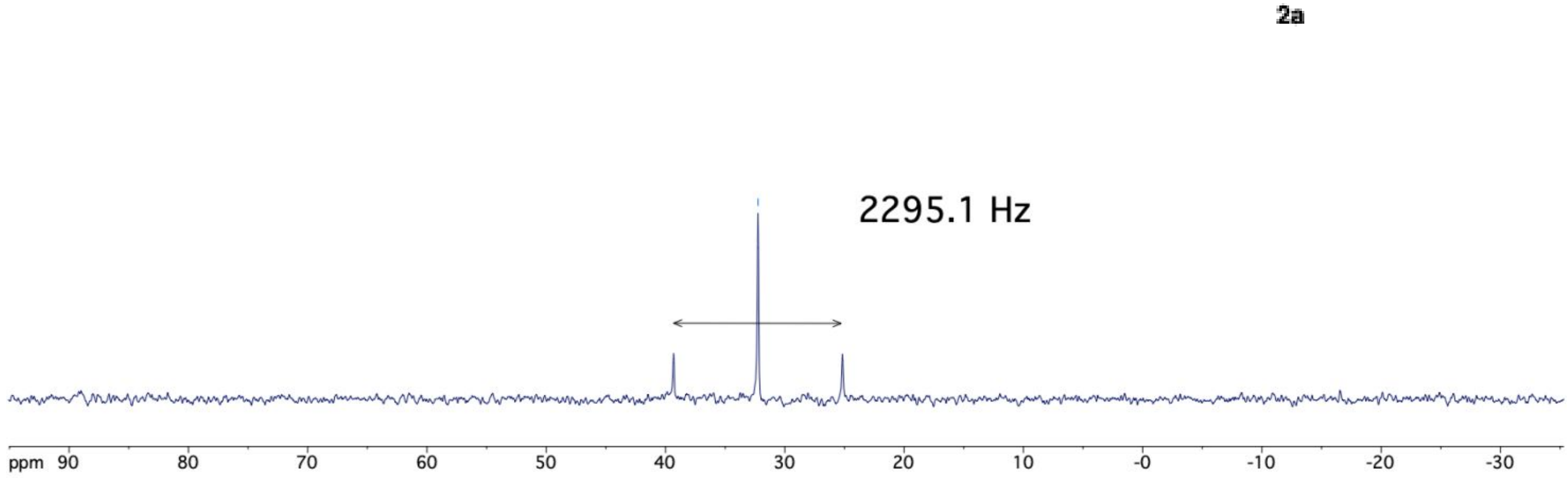

Figure S18. ^{31}P spectrum ($CDCl_3$) of **2a**

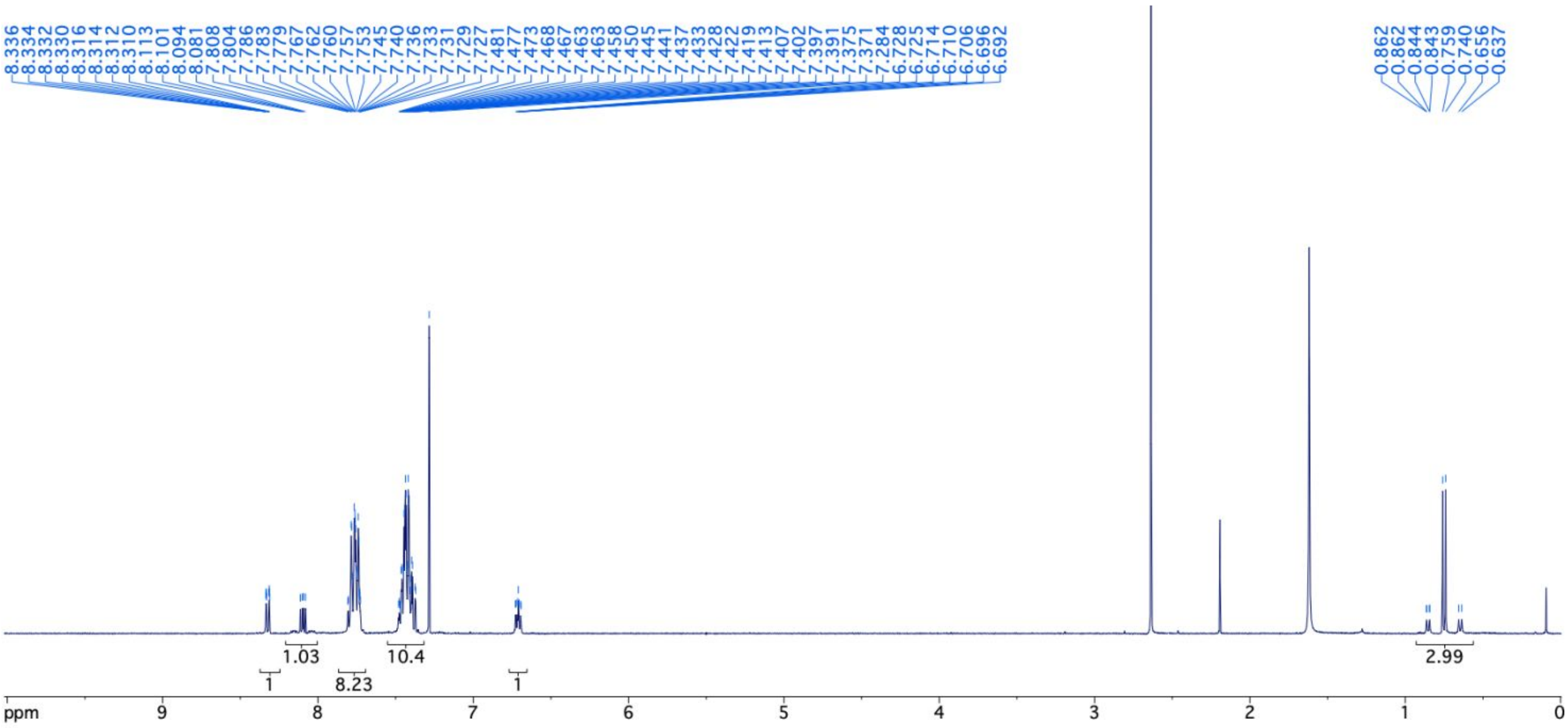

Figure S19. ^{1}H NMR spectrum ($CDCl_3$) of **2b**

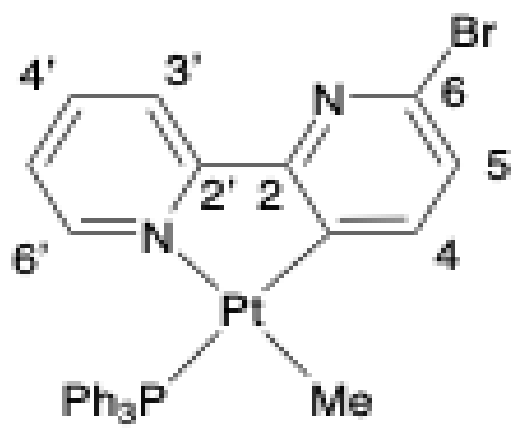

2b

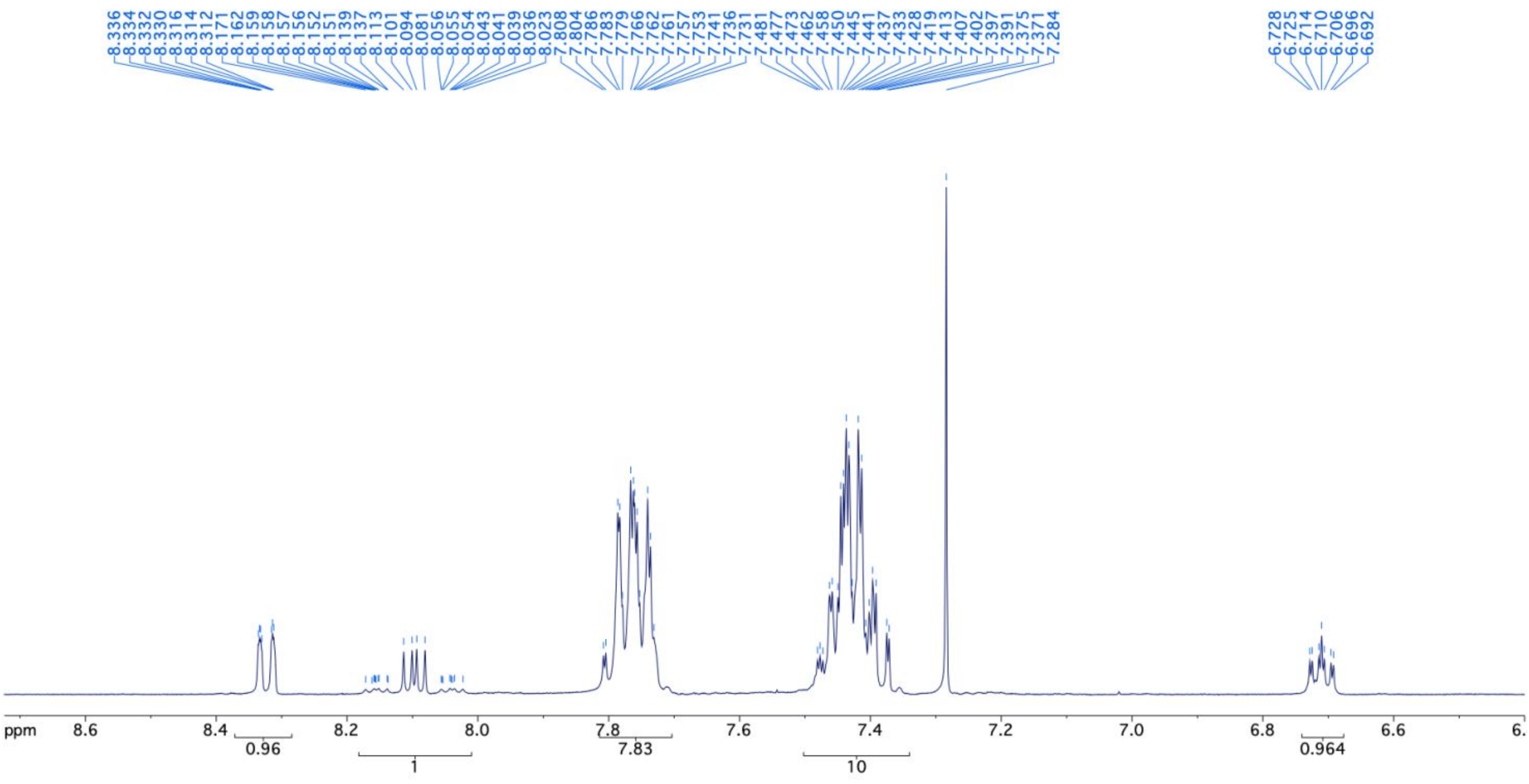

Figure S20. ^{1}H NMR spectrum (aromatic region, $CDCl_3$) of **2b**

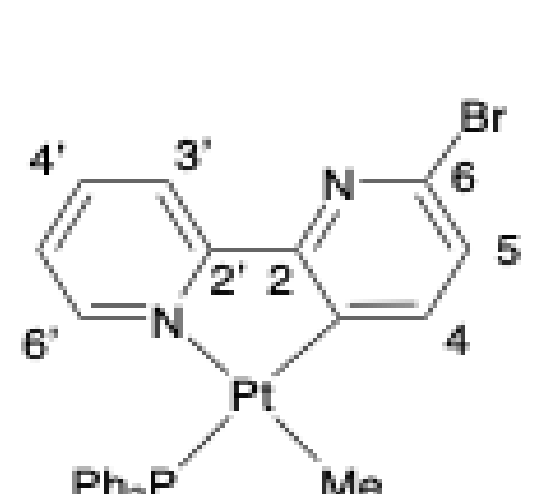

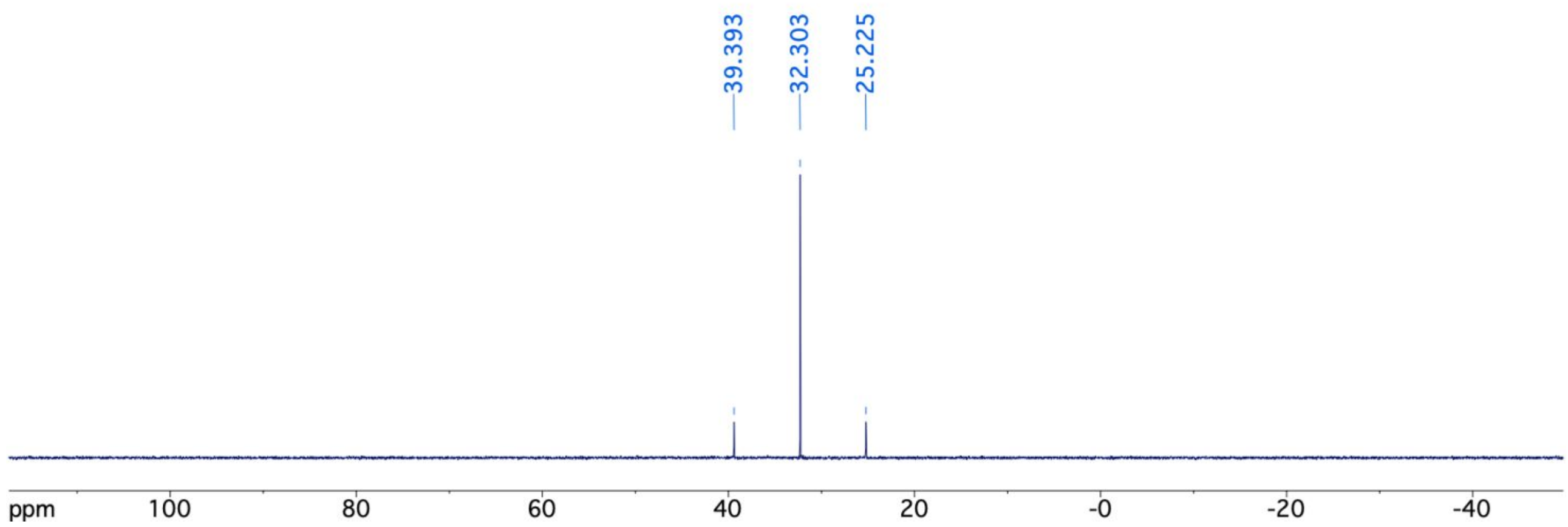

Figure S21. ^{31}P spectrum ($CDCl_3$) of **2b**

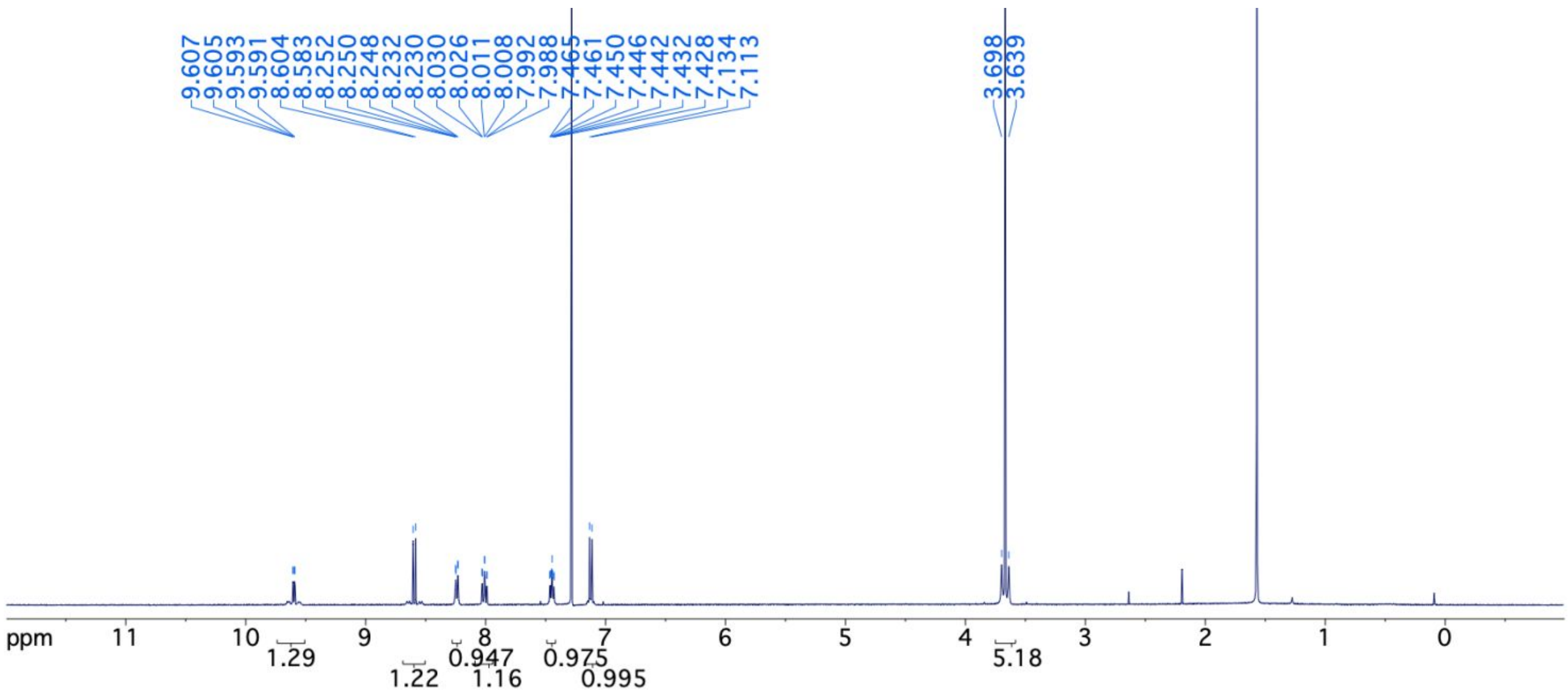

Figure S22. ^{1}H NMR spectrum ($CDCl_3$) of **3a**

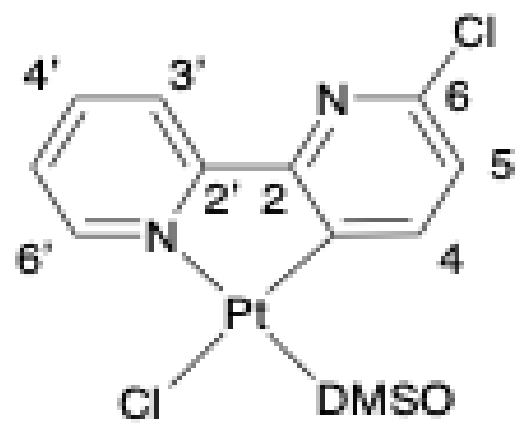

3a

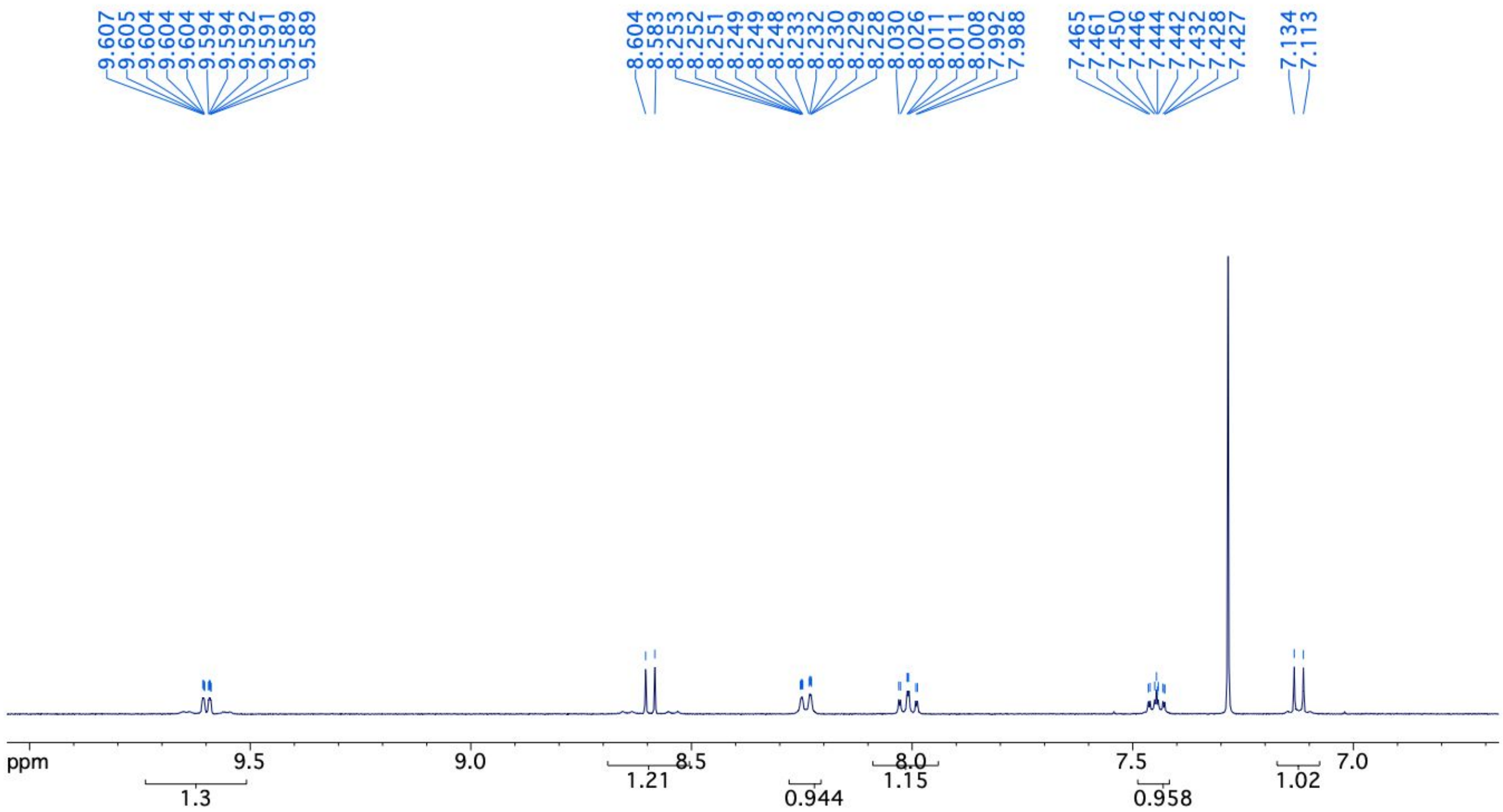

Figure S23. ^{1}H NMR spectrum (aromatic region, $CDCl_3$) of **3a**

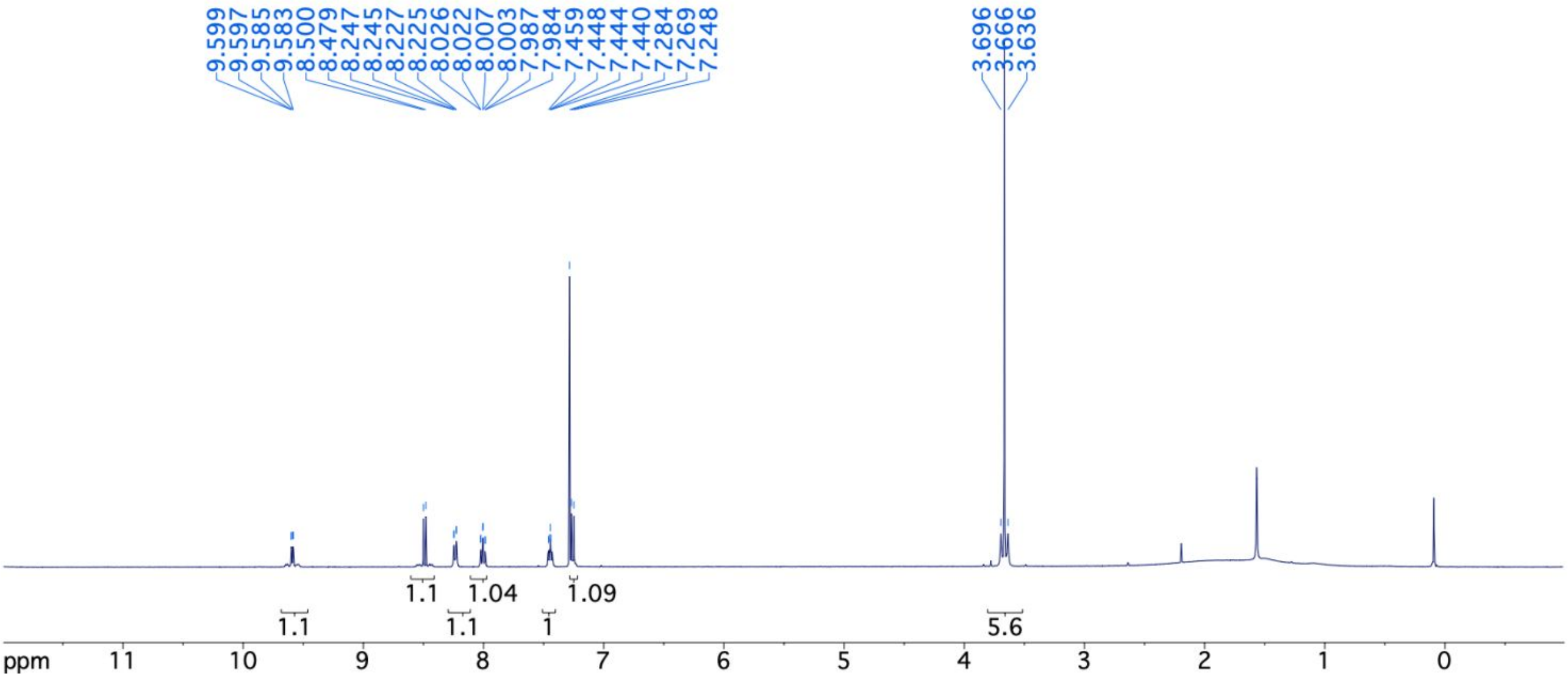

Figure S24. ^{1}H NMR spectrum ($CDCl_3$) of **3b**

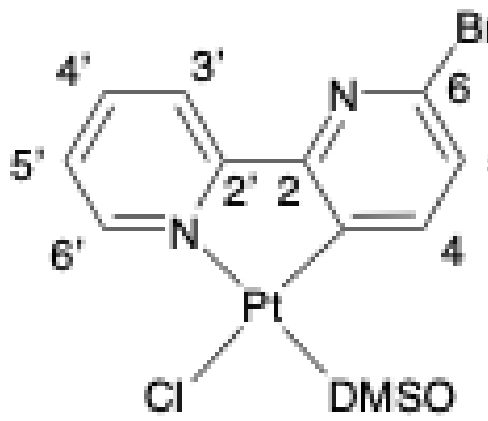

3b

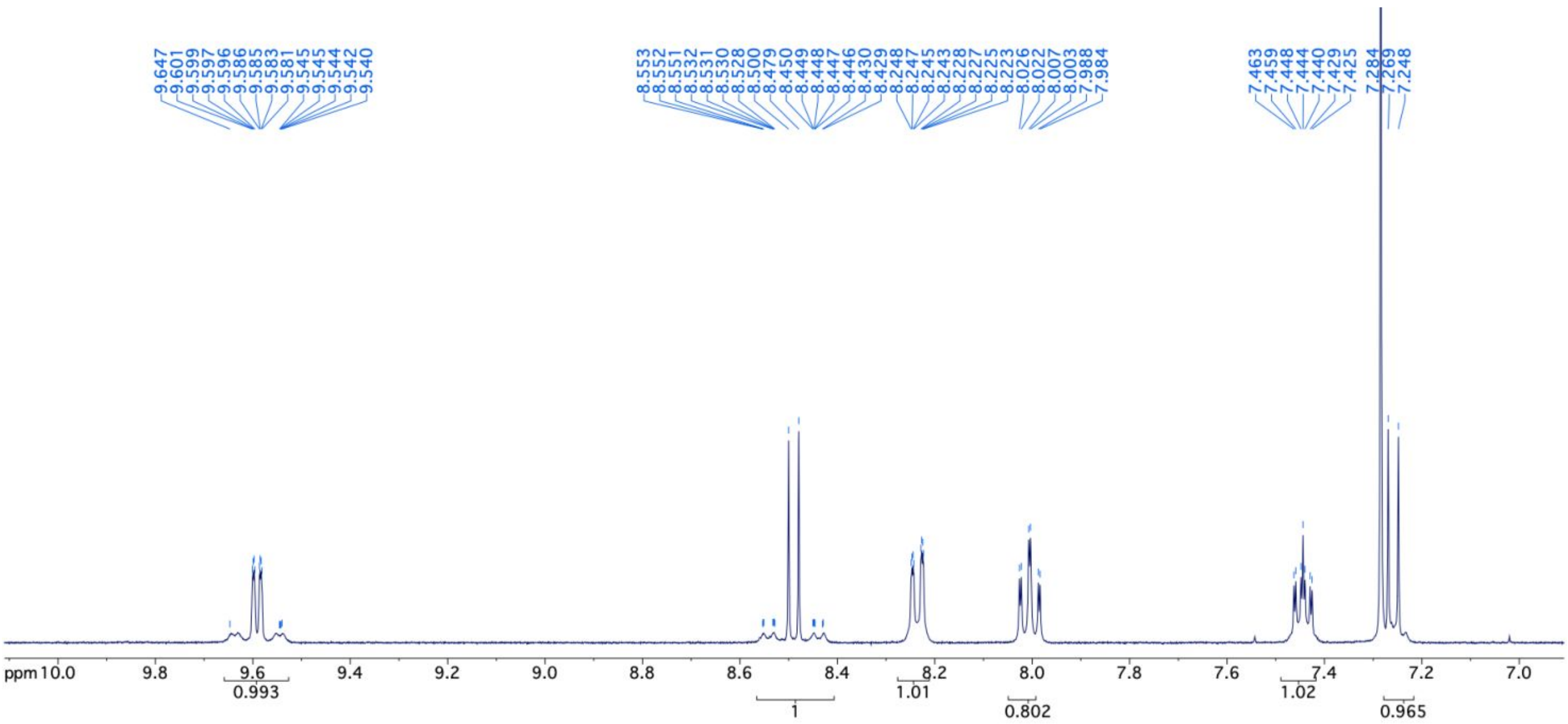

Figure S25. ^{1}H NMR spectrum (aromatic region, $CDCl_3$) of **3b**

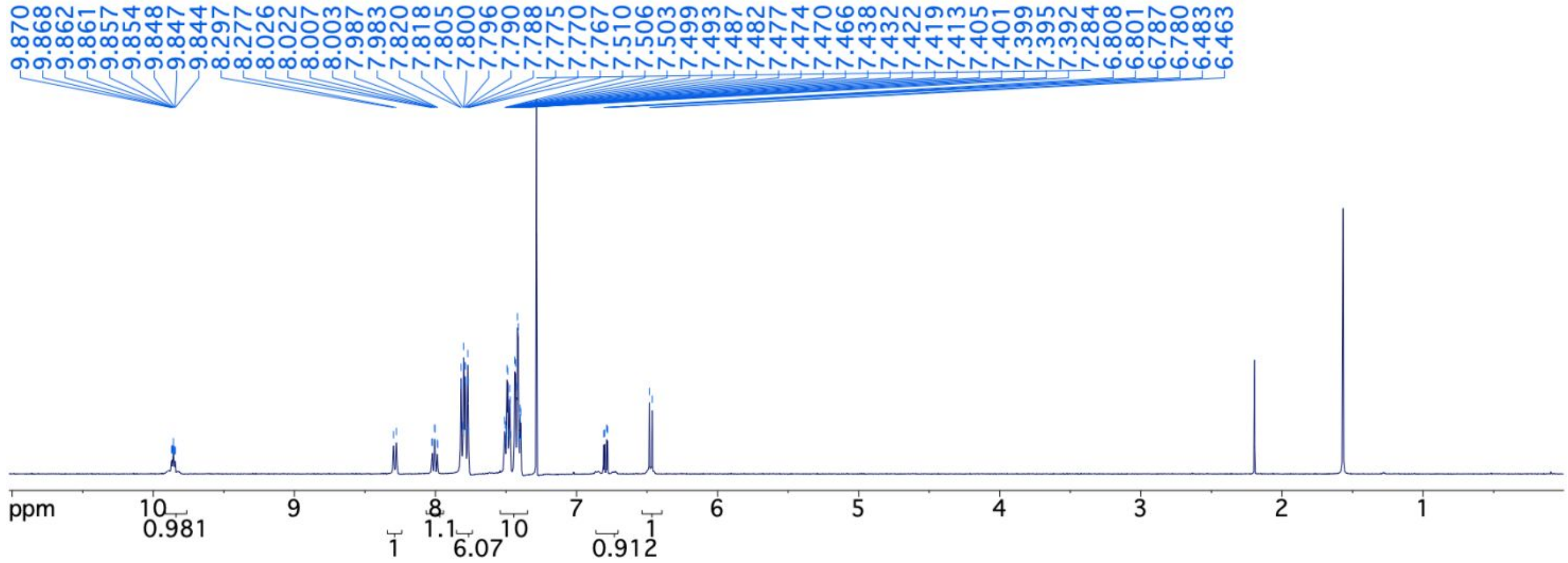

Figure S26. ^{1}H NMR spectrum ($CDCl_3$) of **4a**

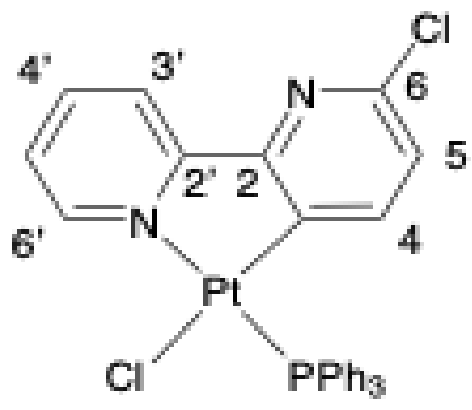

4a

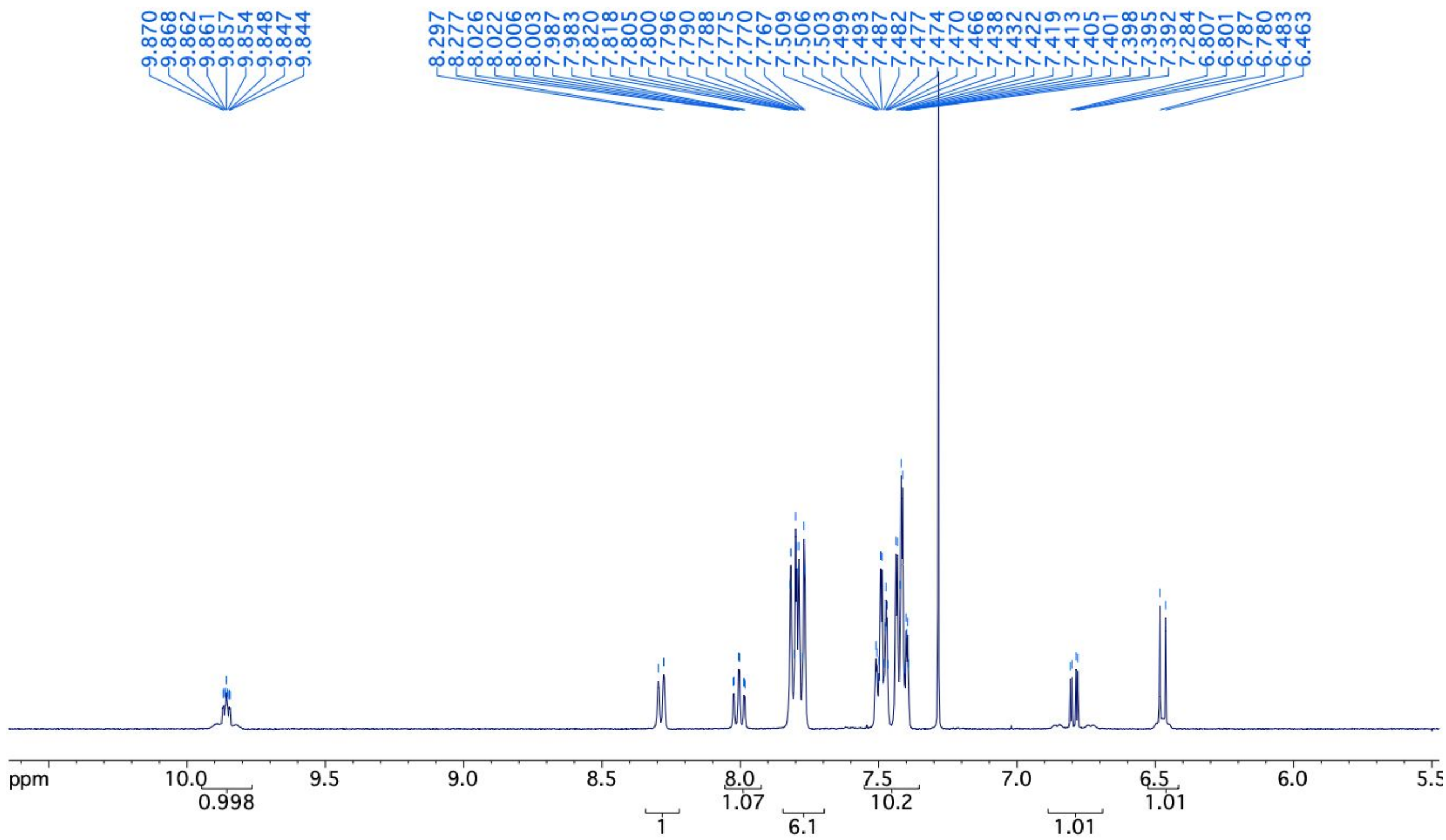

Figure S27. ^{1}H NMR spectrum (aromatic region, $CDCl_3$) of **4a**

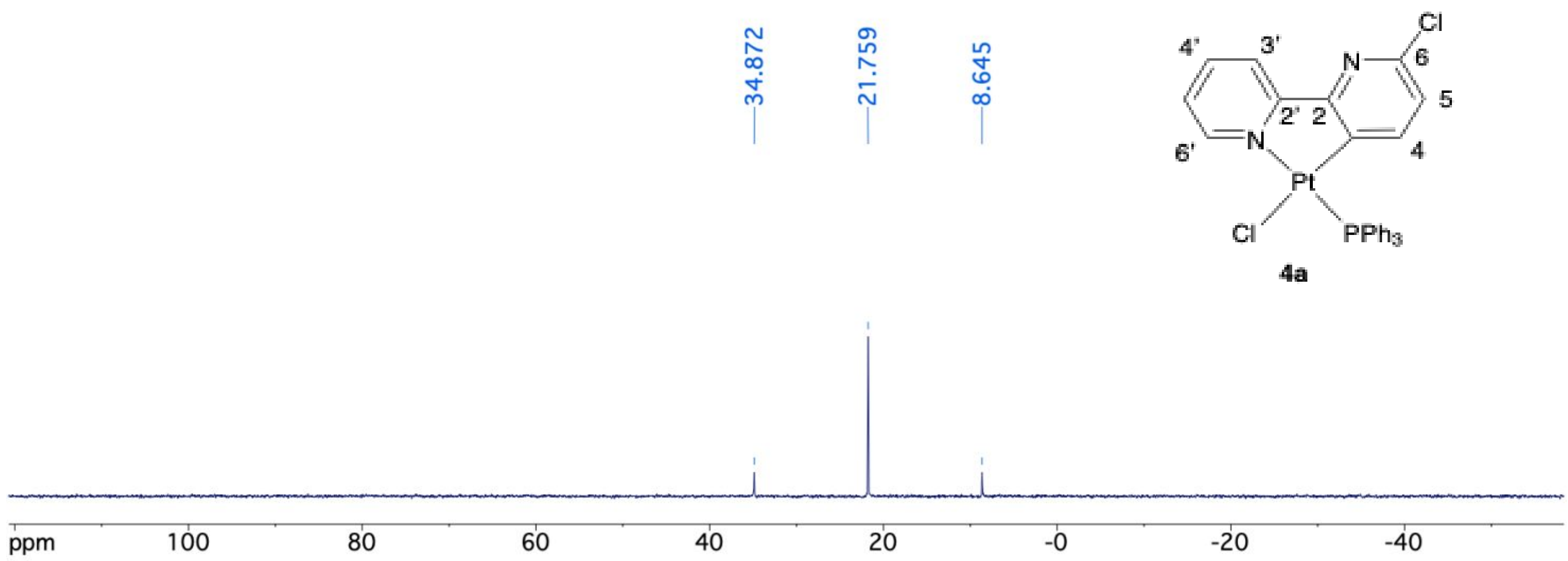

Figure S28. ^{31}P spectrum ($CDCl_3$) of **4a**

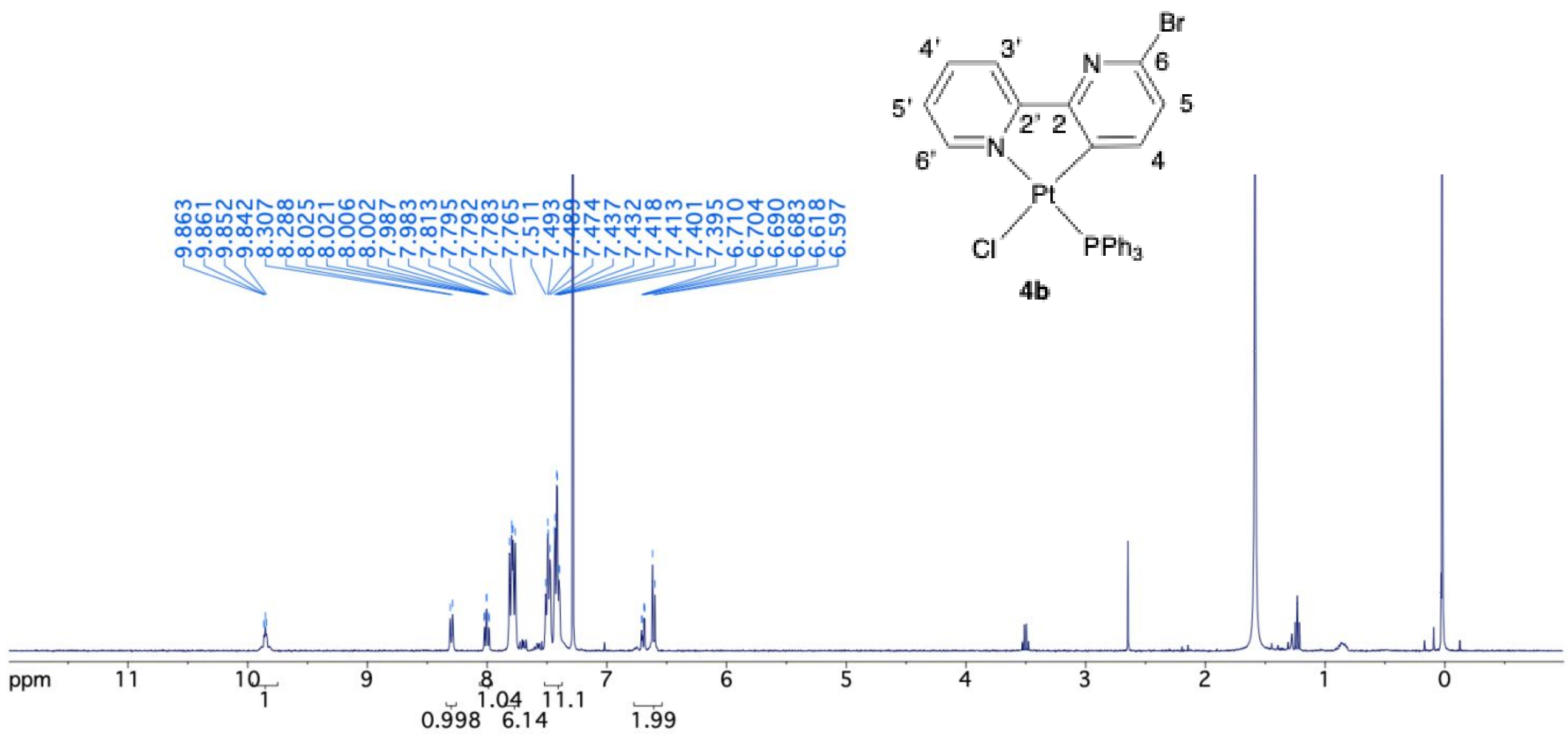

Figure S29. ^{1}H NMR spectrum ($CDCl_3$) of **4b**

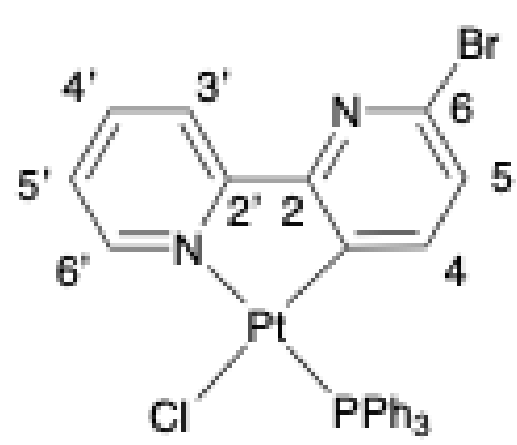

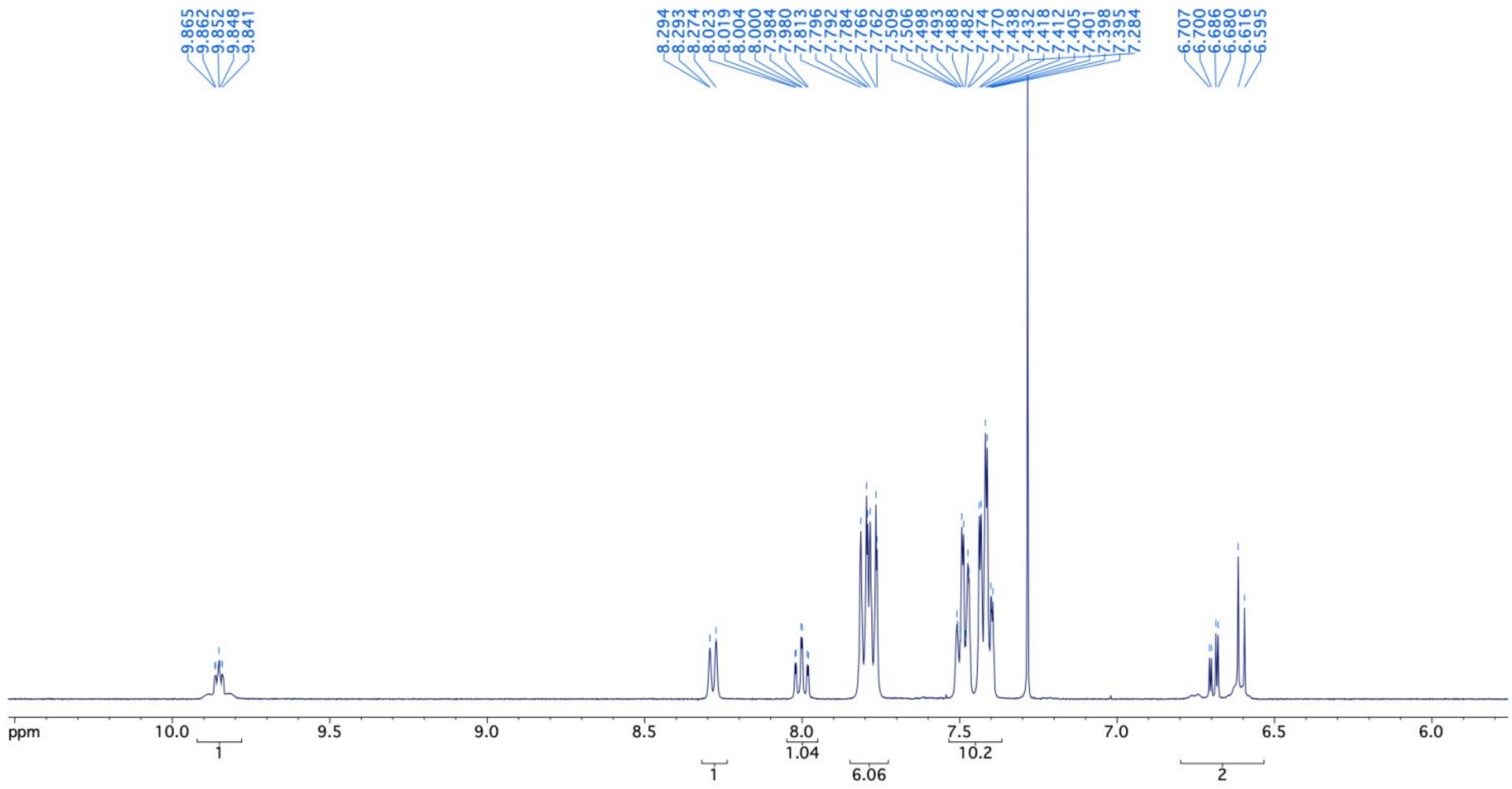

Figure S30. ^{1}H NMR spectrum (aromatic region, $CDCl_3$) of **4b**

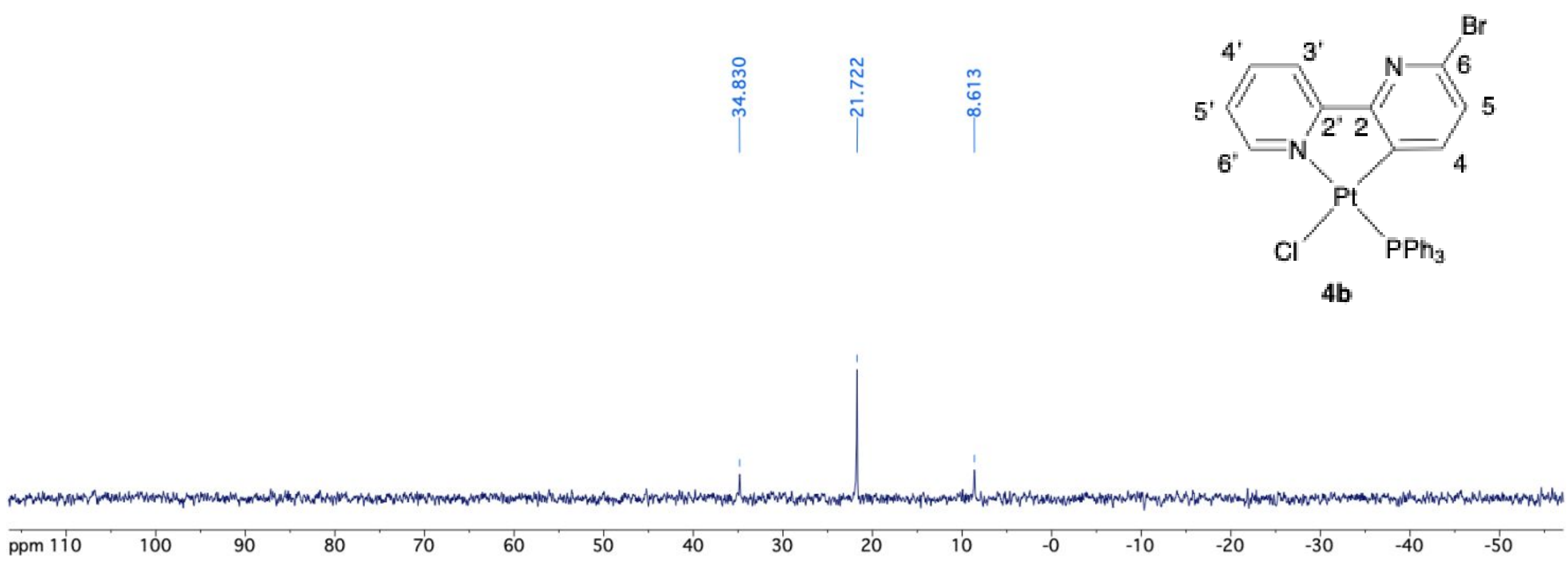

Figure S31. ^{31}P NMR spectrum ($CDCl_3$) of **4b**

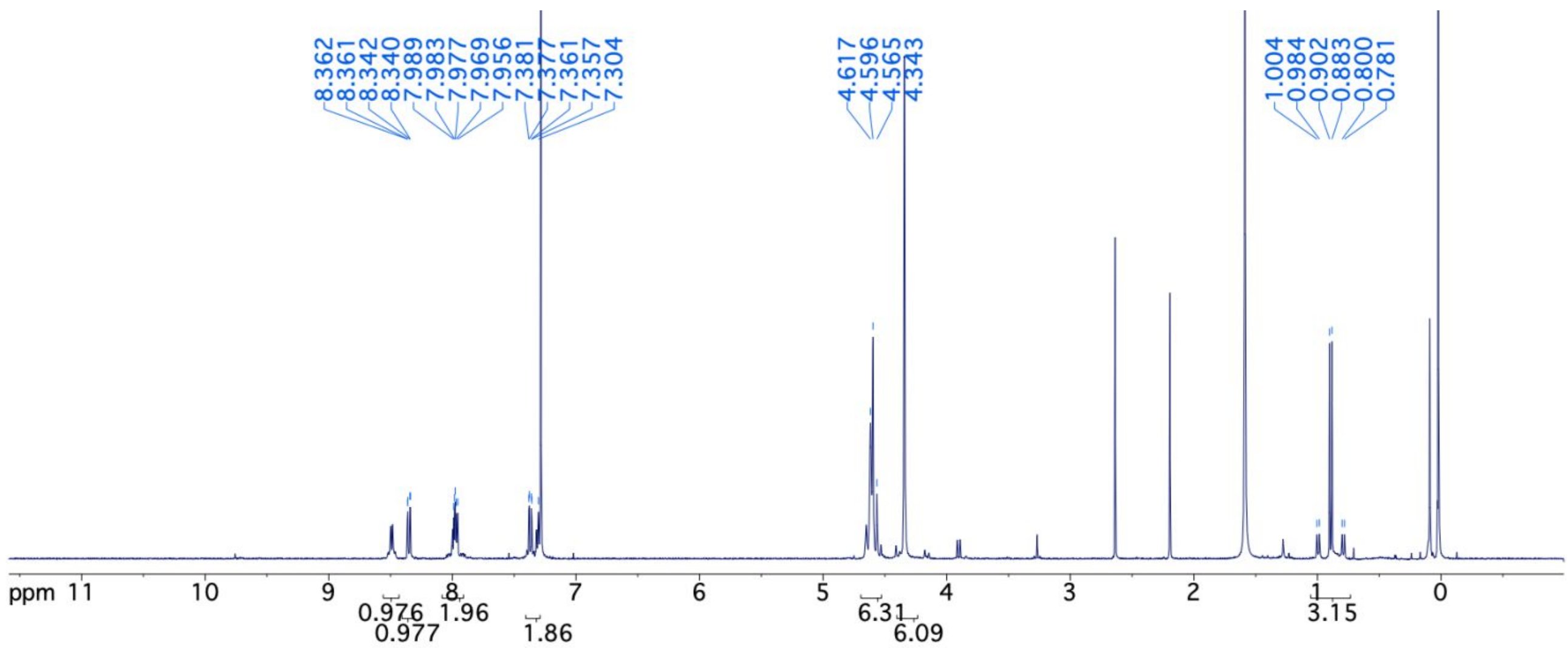

Figure S32. ^{1}H NMR spectrum ($CDCl_3$) of **5b**

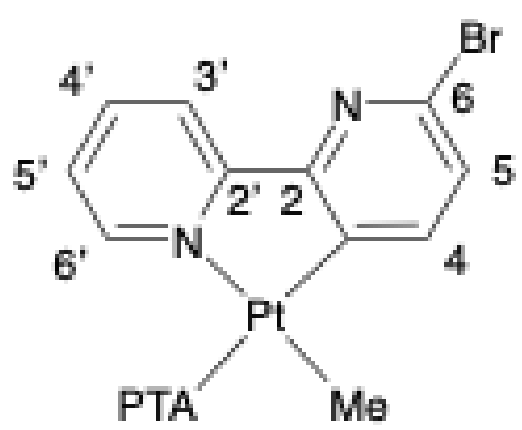

5b

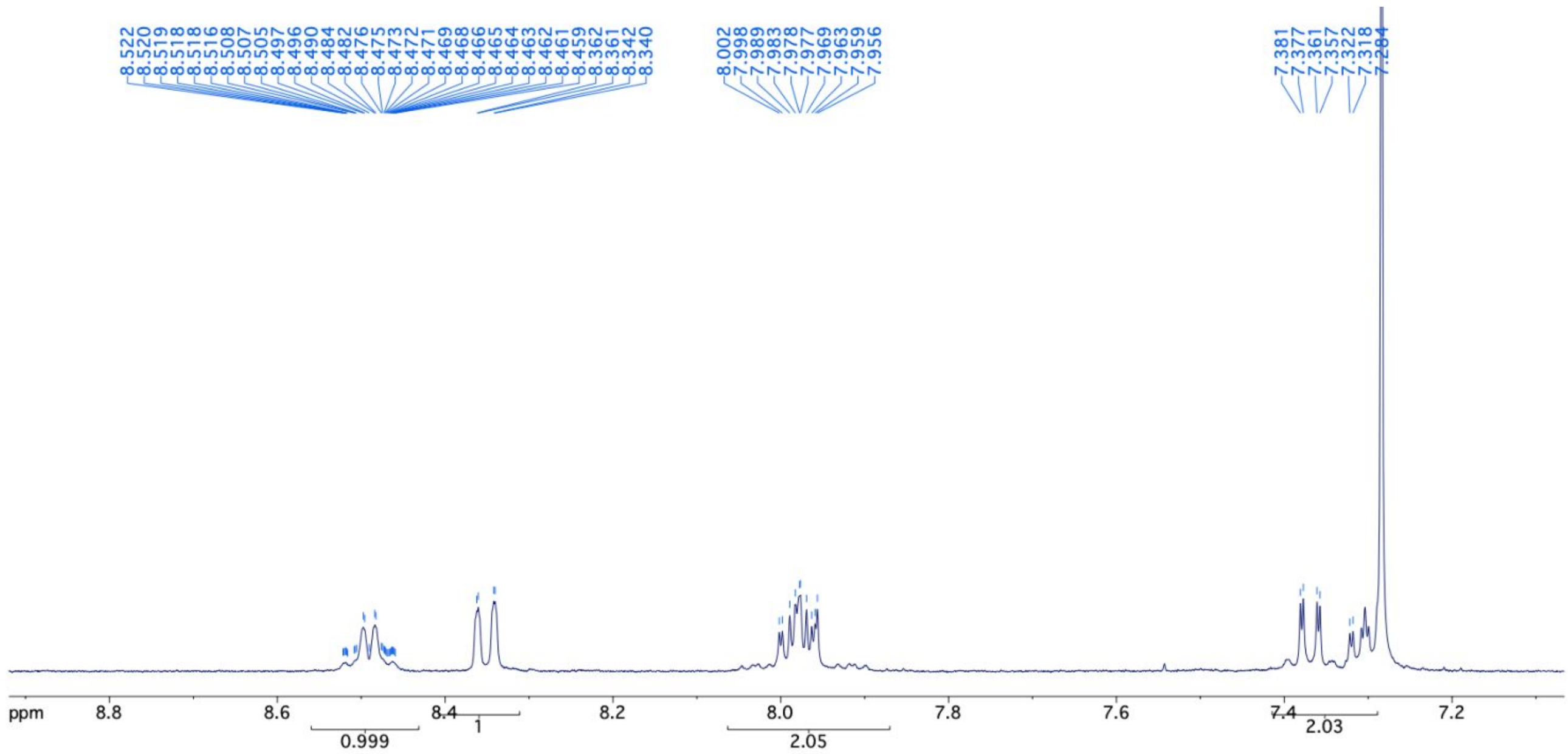

Figure S33. ^{1}H NMR spectrum (aromatic region, $CDCl_3$) of **5b**

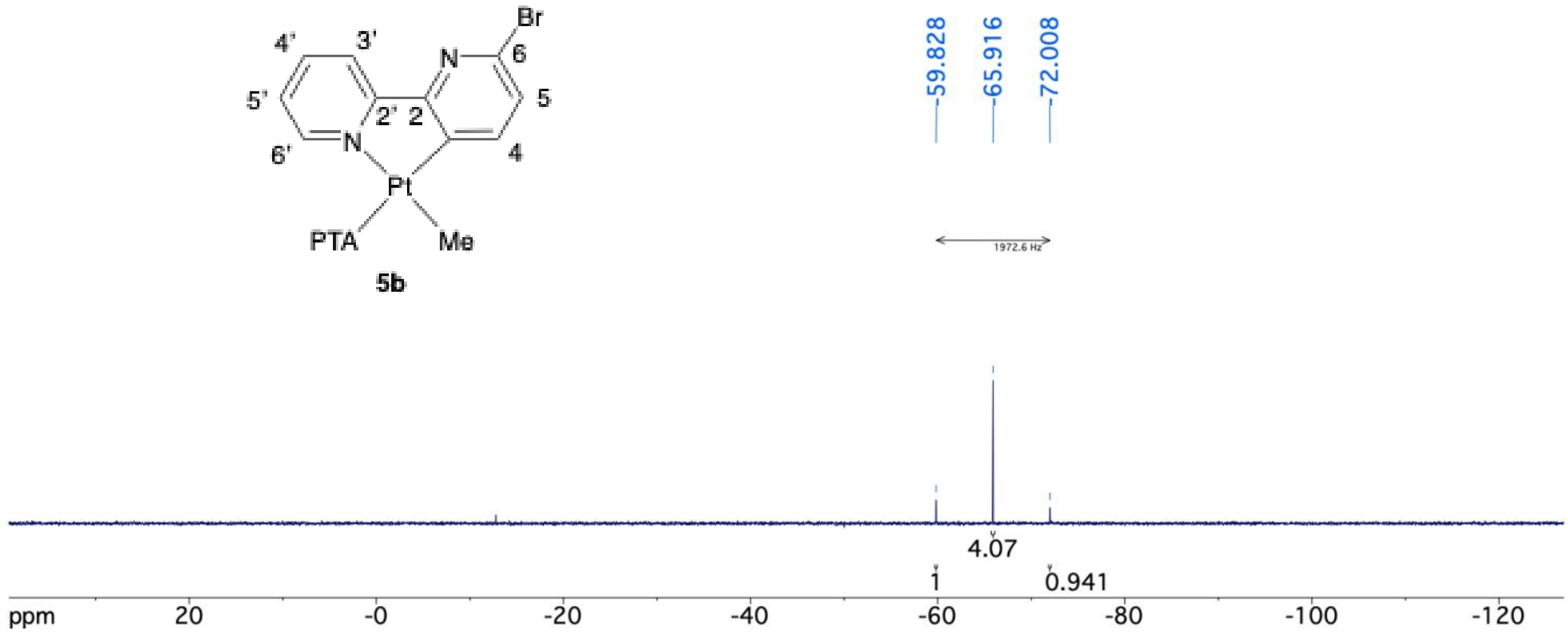

Figure S34. ^{31}P NMR spectrum ($CDCl_3$) of **5b**

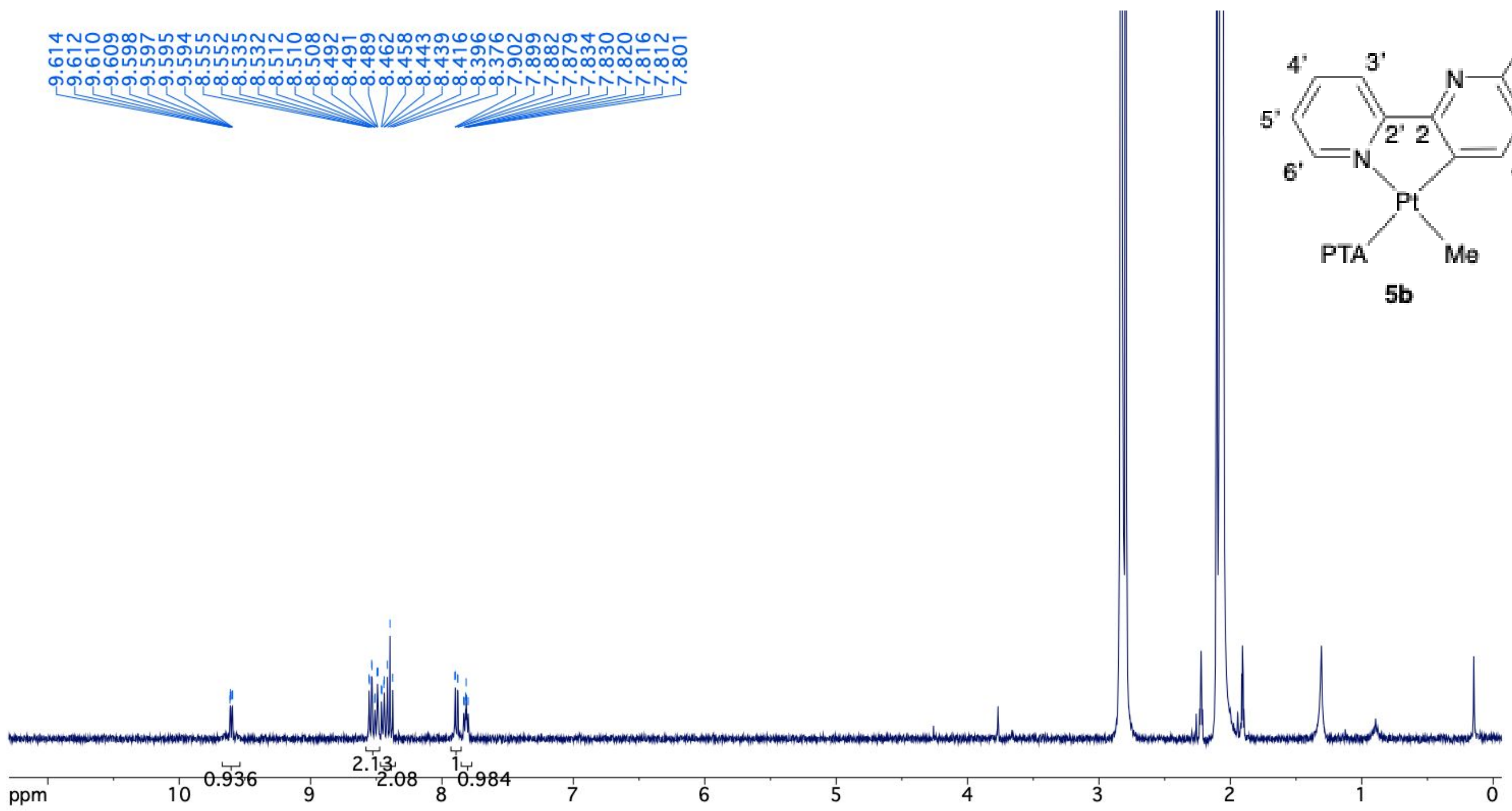

Figure S35. ^{1}H NMR spectrum (acetone-d6) of **6a**

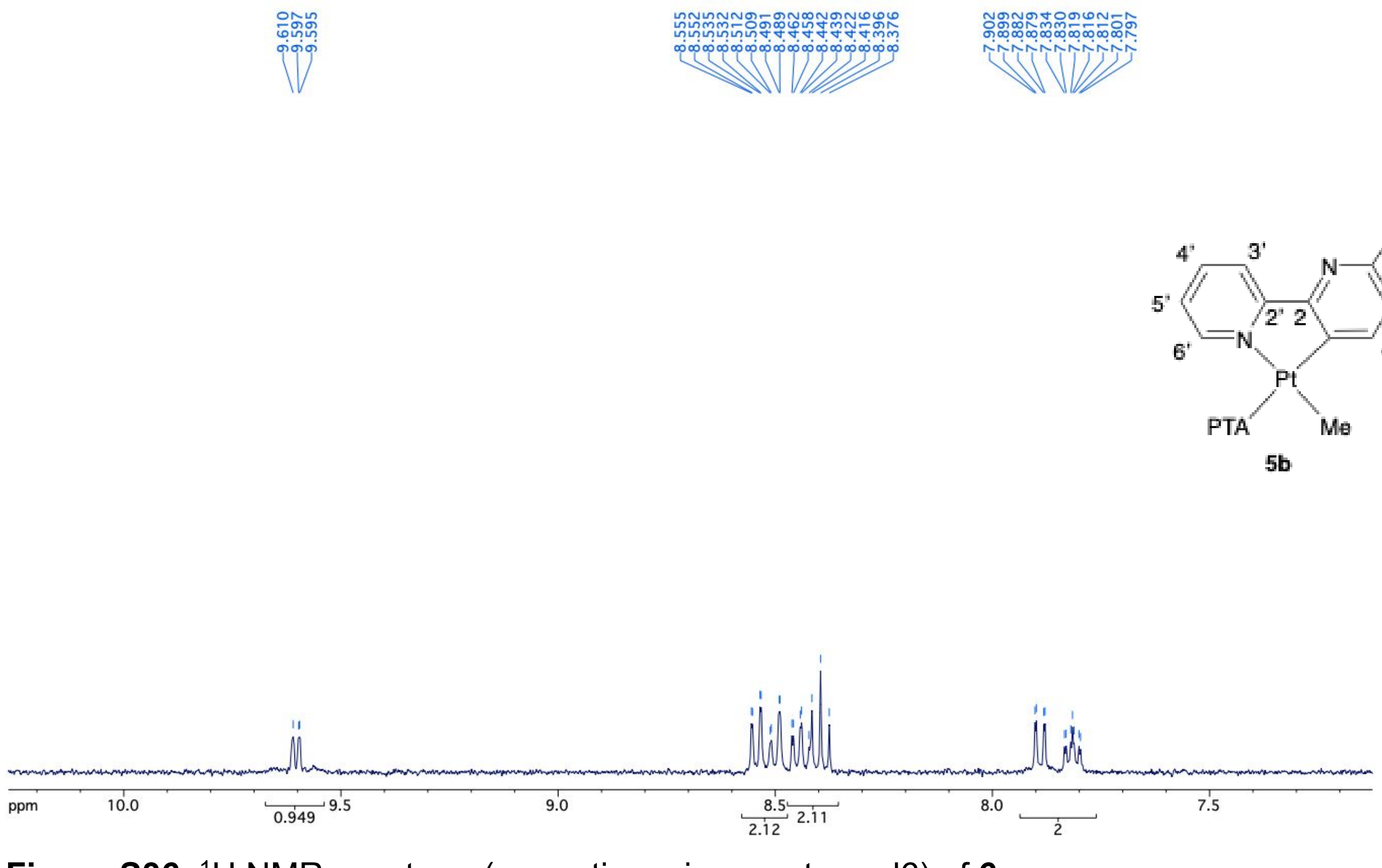

Figure S36. ^{1}H NMR spectrum (aromatic region, acetone-d6) of **6a**

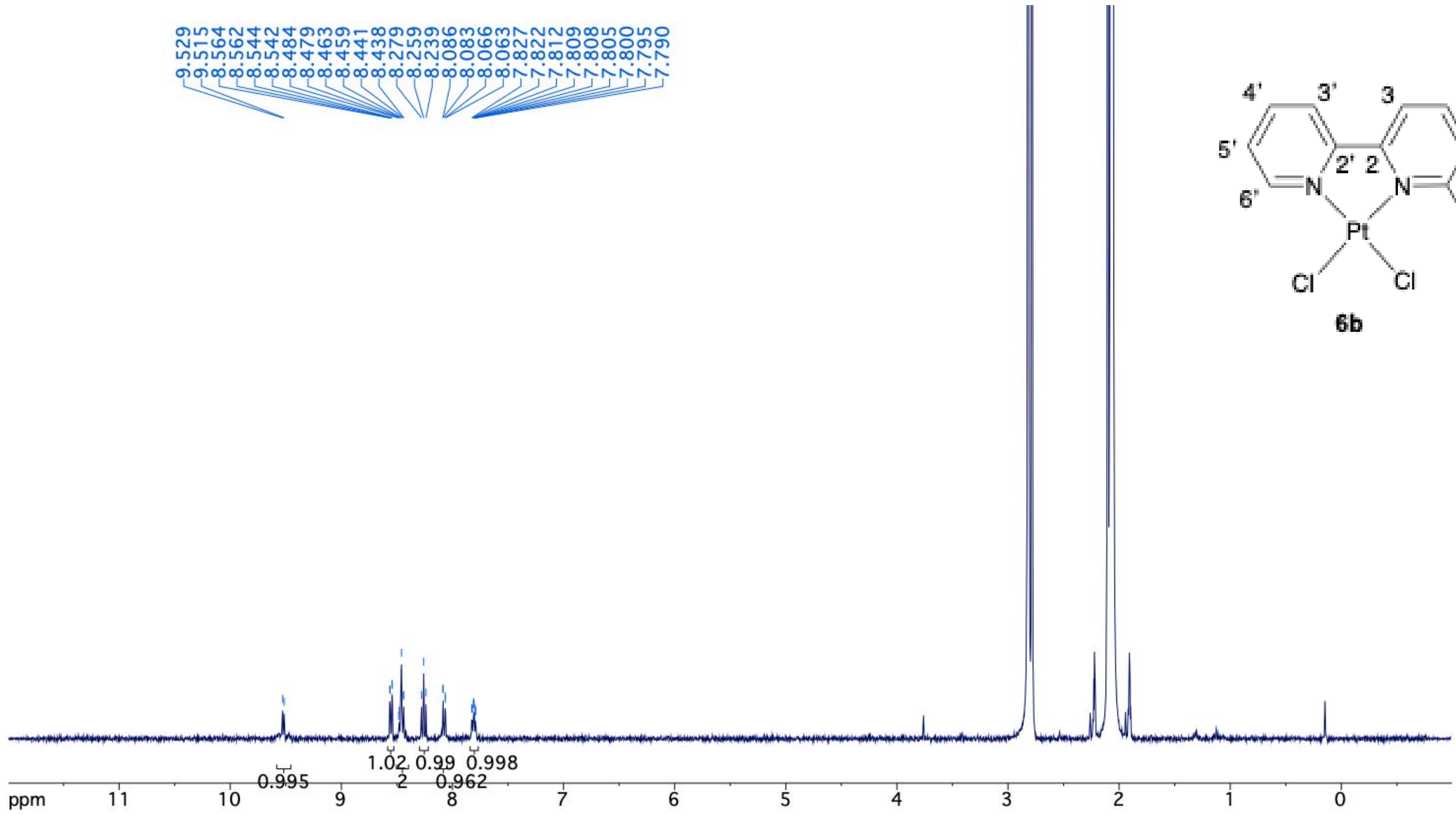

Figure S37. ^{1}H NMR spectrum (acetone-d6) of **6b**

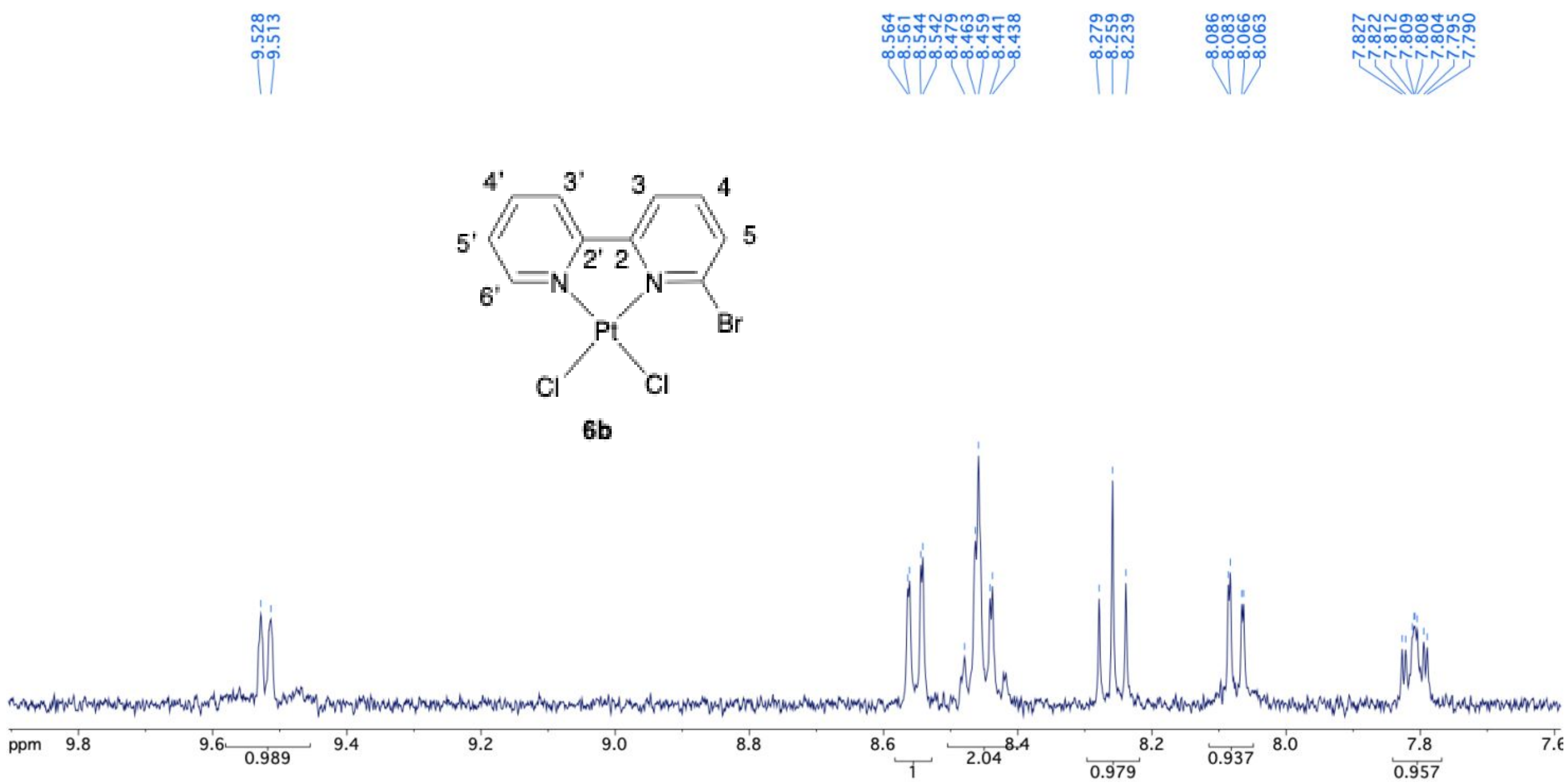

Figure S38. ^{1}H NMR spectrum (aromatic region, acetone-d6) of **6b**

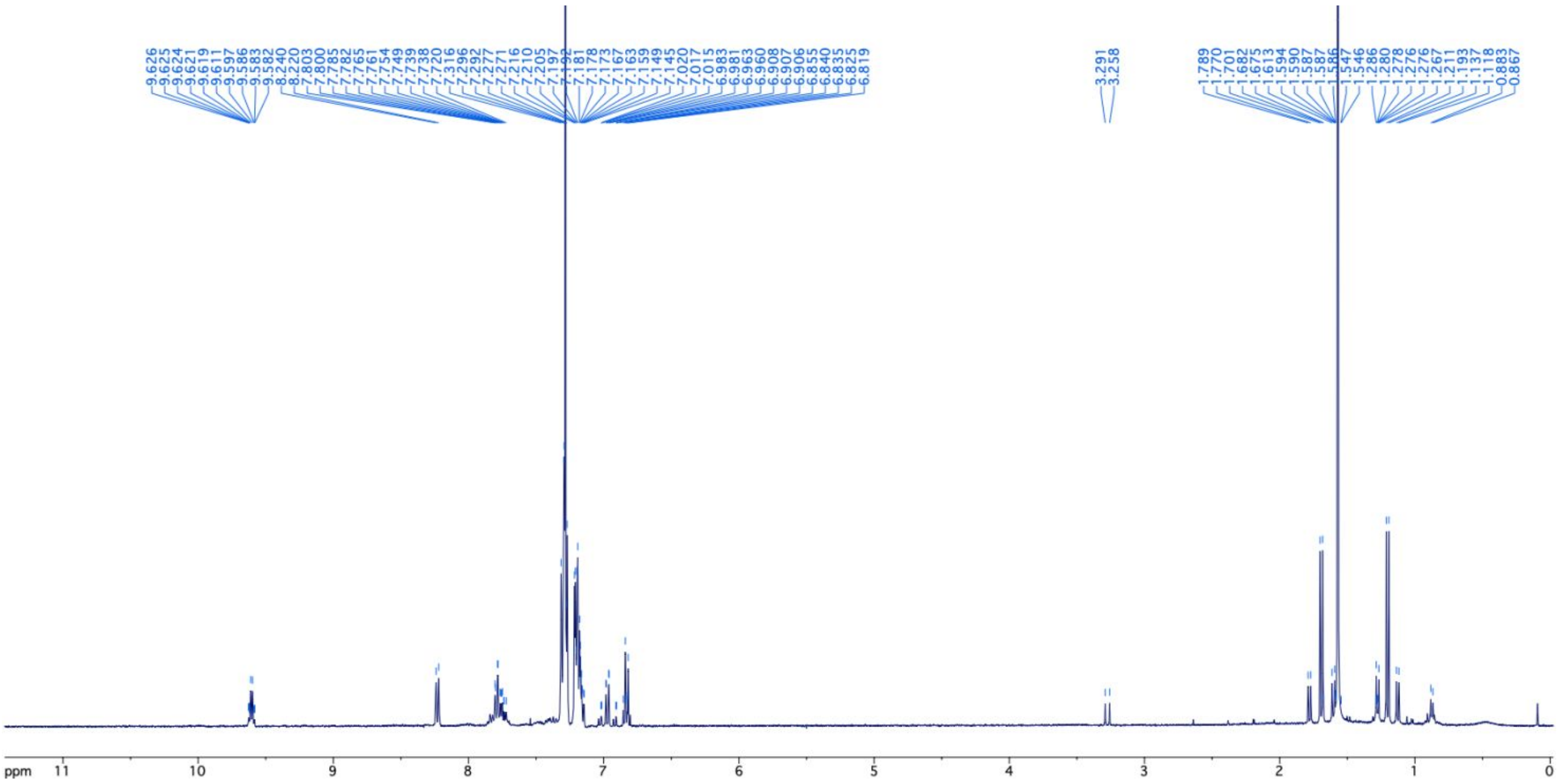

Figure S39. ^{1}H NMR spectrum ($CDCl_3$) of **7a**.

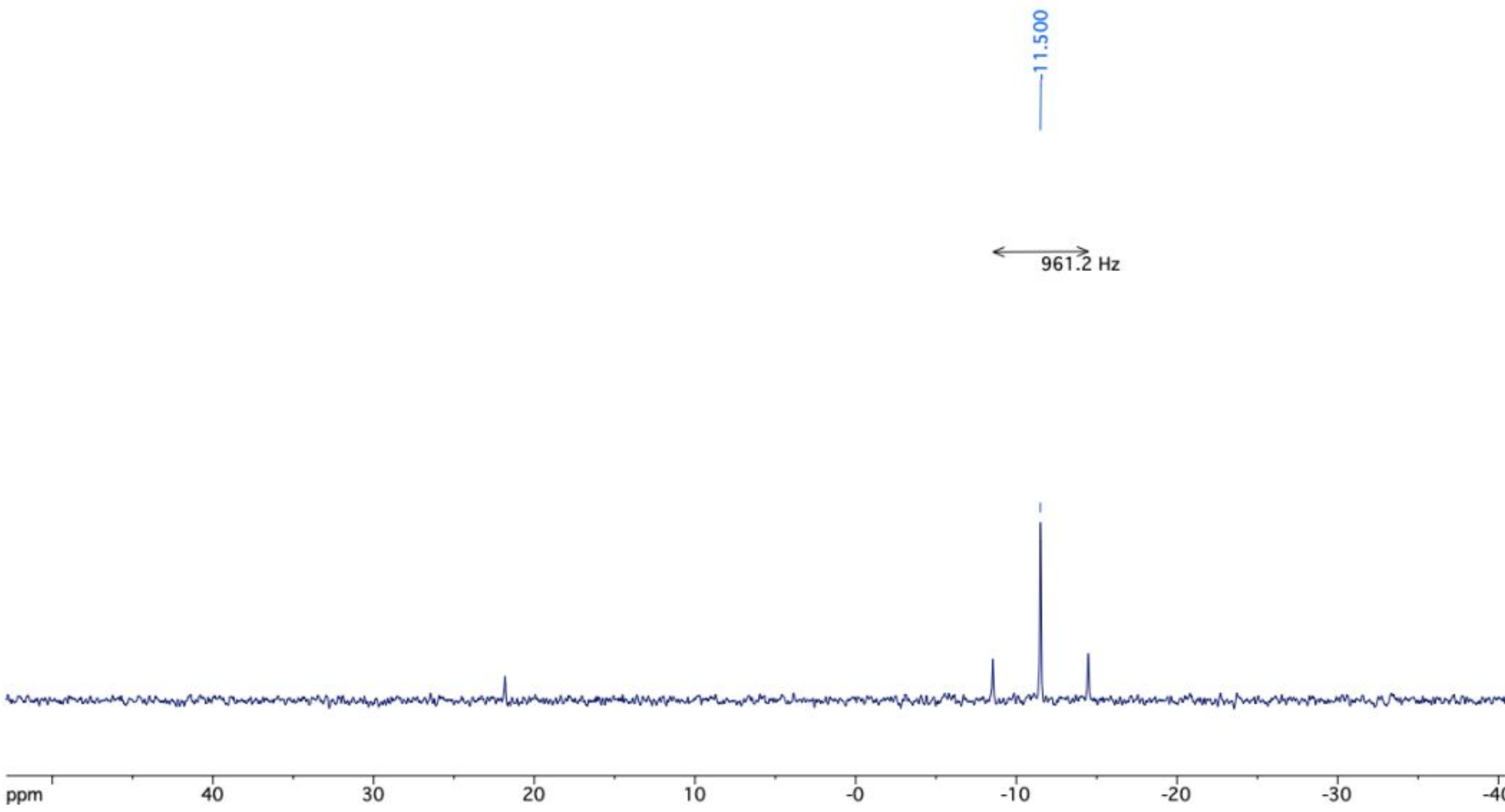

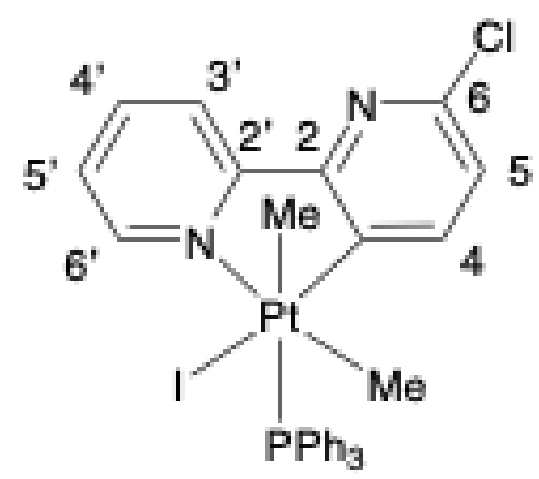

7a

Figure S40. ^{31}P NMR spectrum ($CDCl_3$) of **7a**.

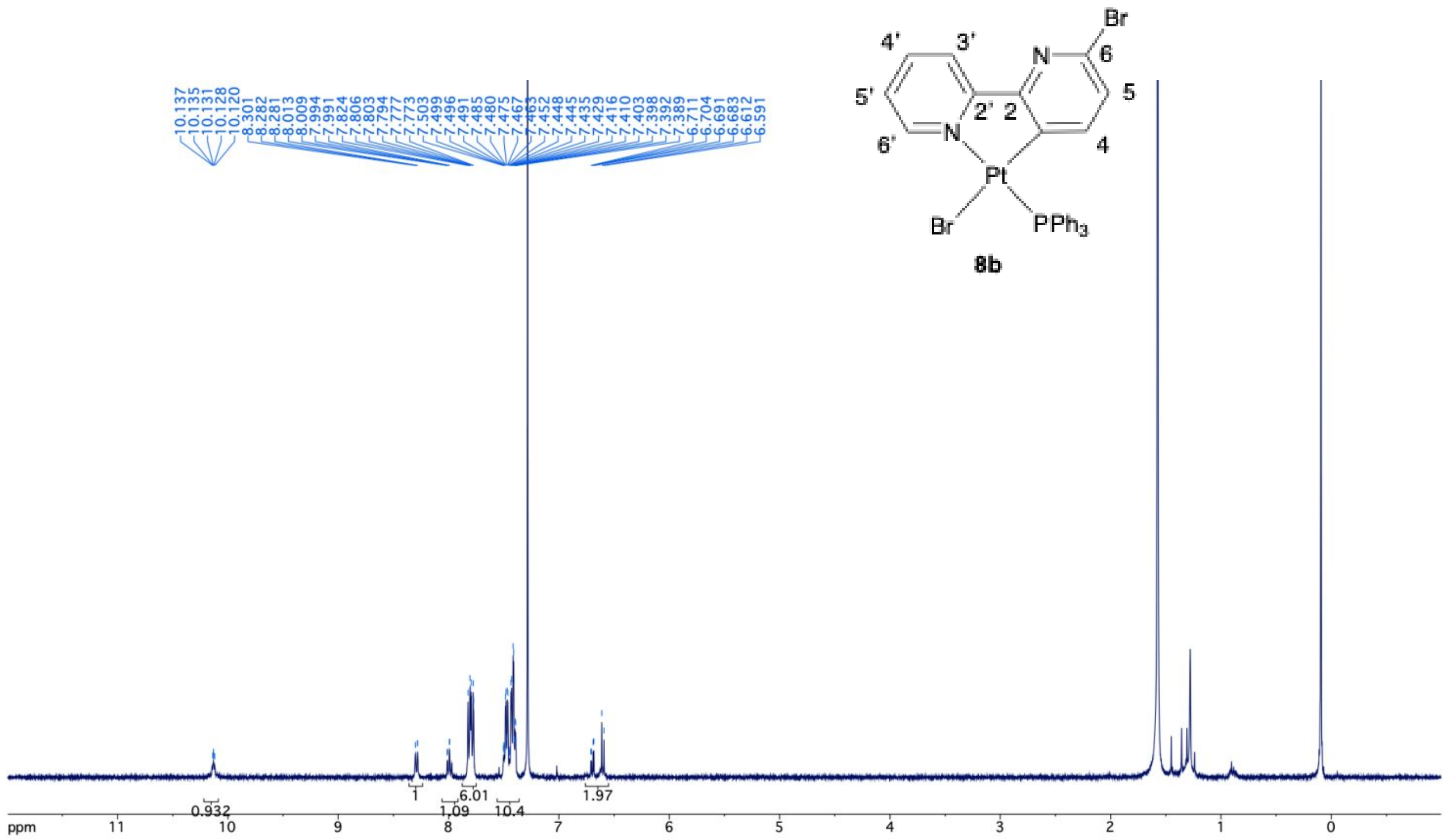

Figure S41. ^{1}H NMR spectrum ($CDCl_3$) of **8b**

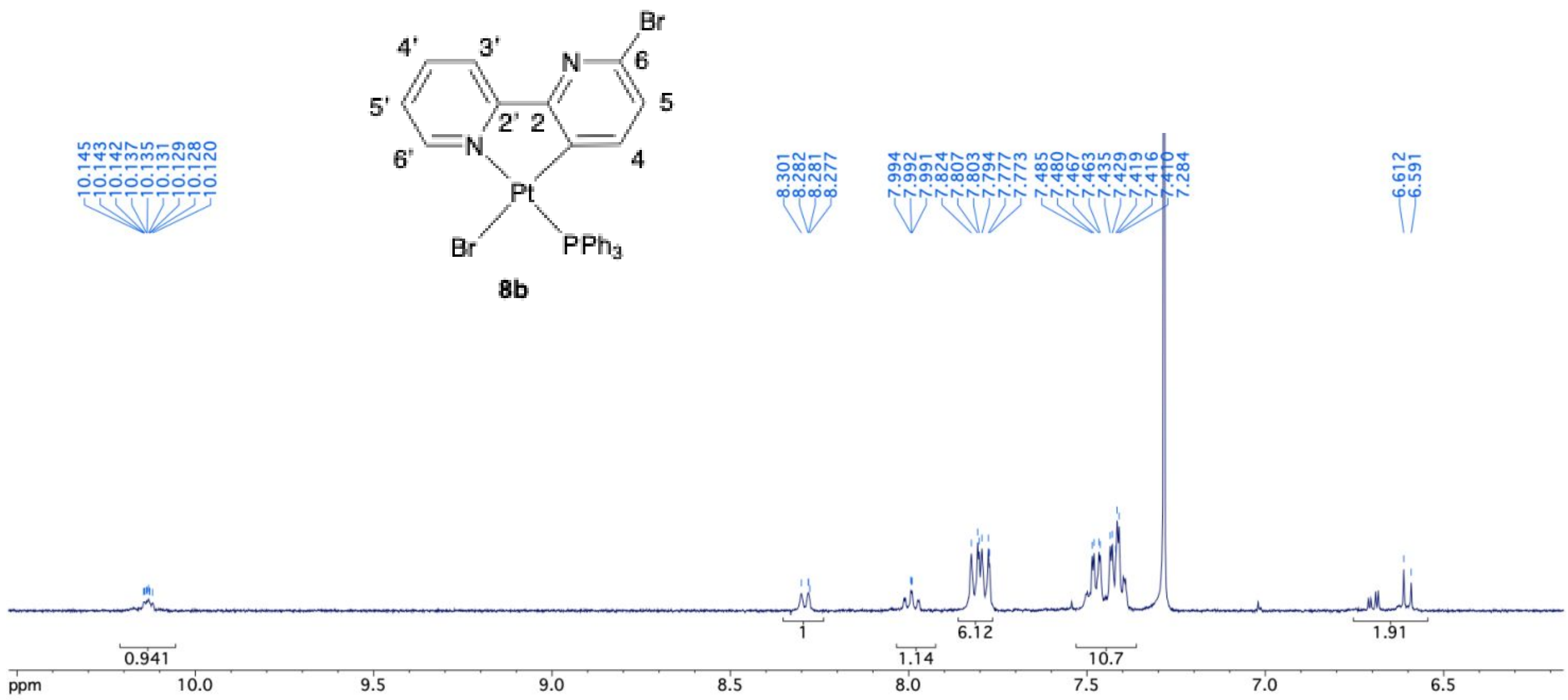

Figure S42. ^{1}H NMR spectrum (aromatic region, $CDCl_3$) of **8b**

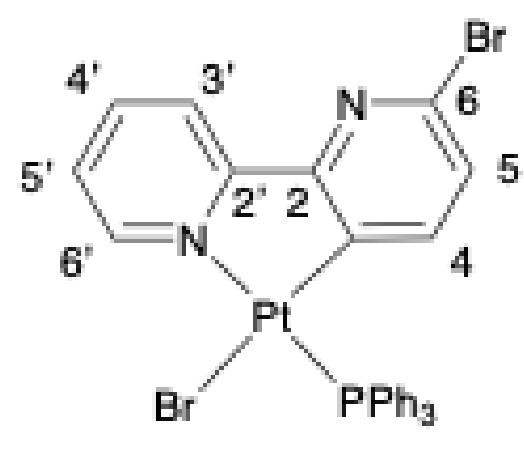

8b

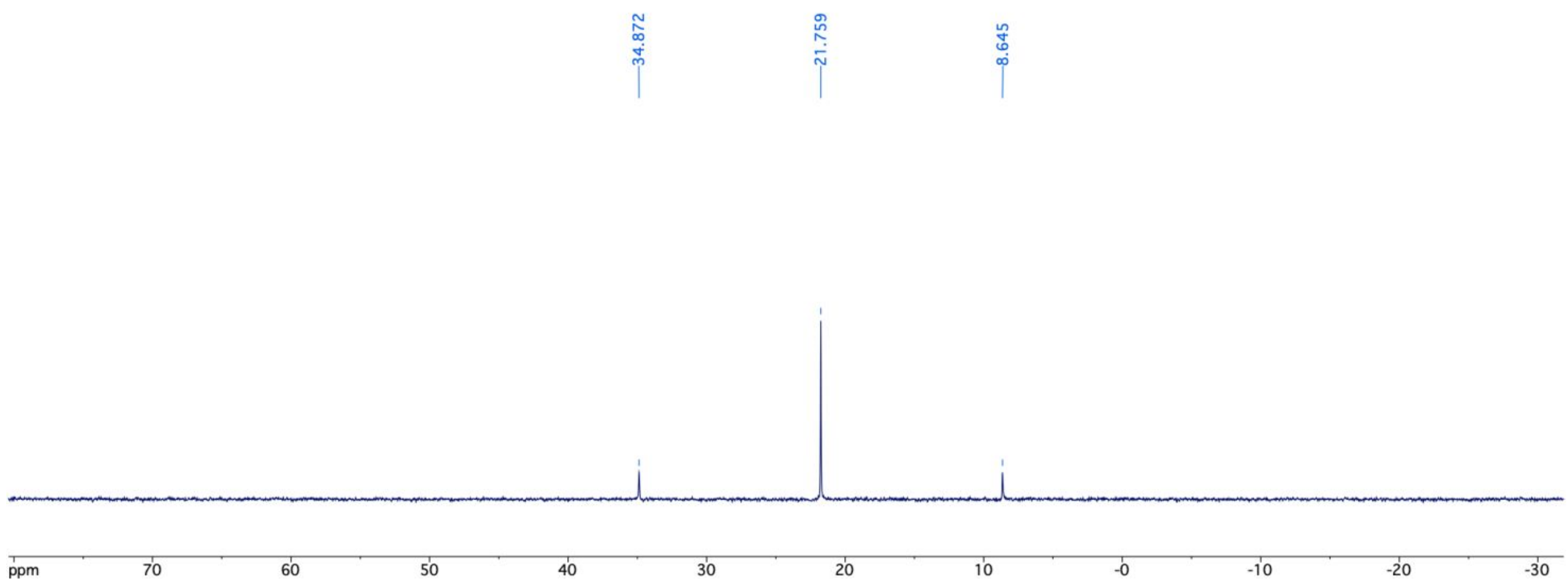

Figure S43. ^{31}P NMR spectrum ($CDCl_3$) of **8b**

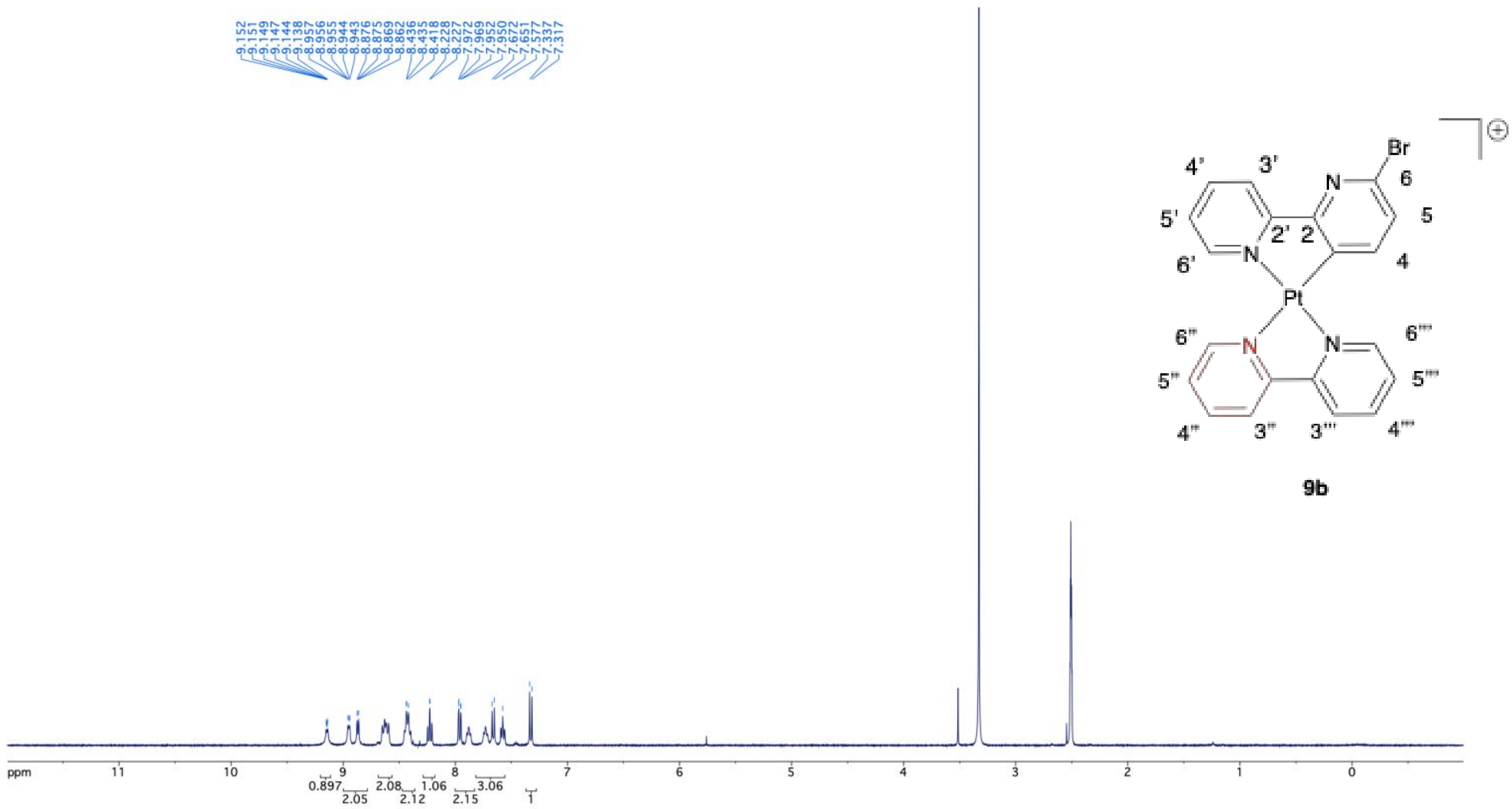

Figure S44. ^{1}H NMR spectrum ($CDCl_3$) of **9b**

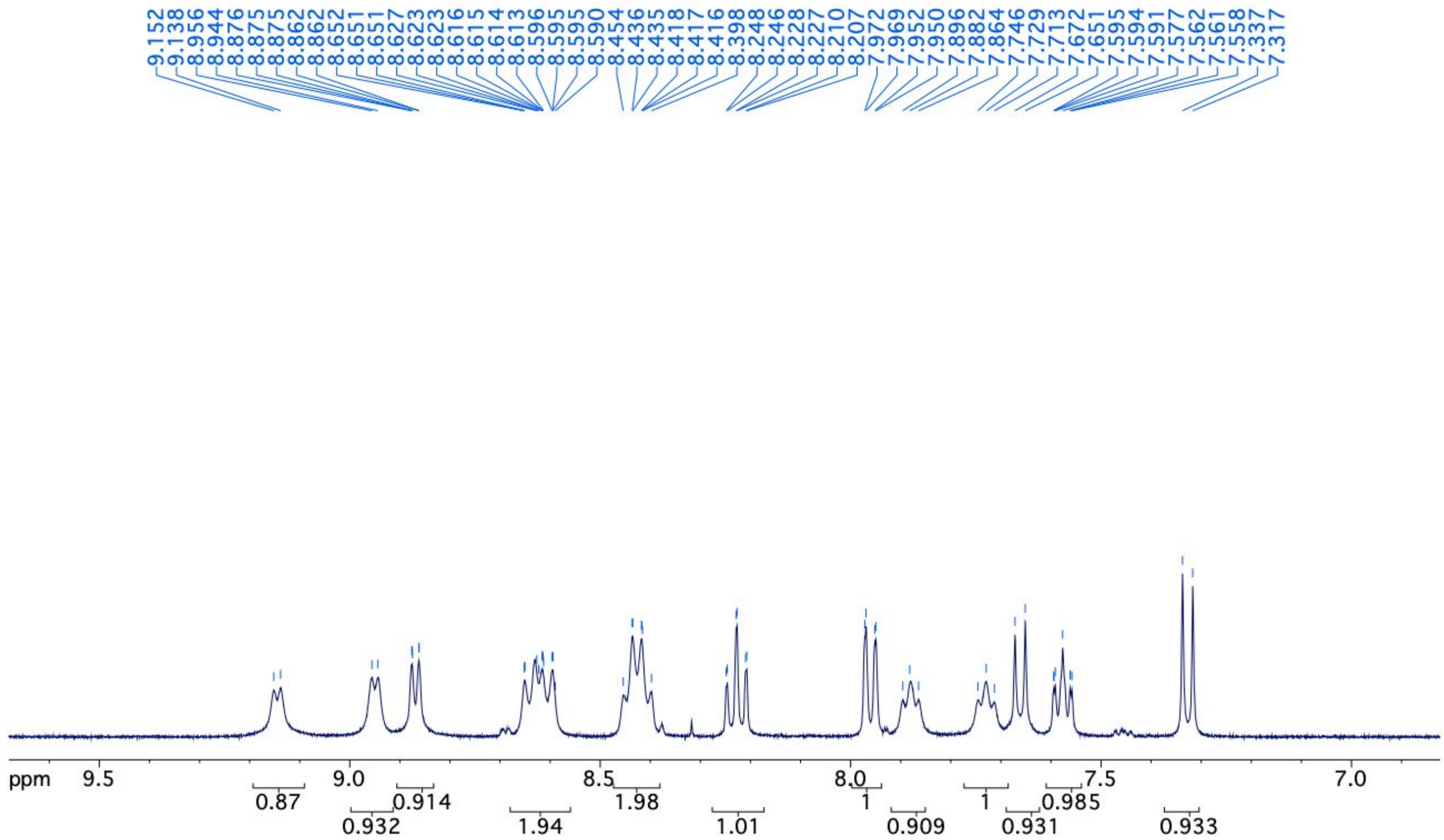

Figure S45. ^{1}H NMR spectrum (aromatic region, $CDCl_3$) of **9b**

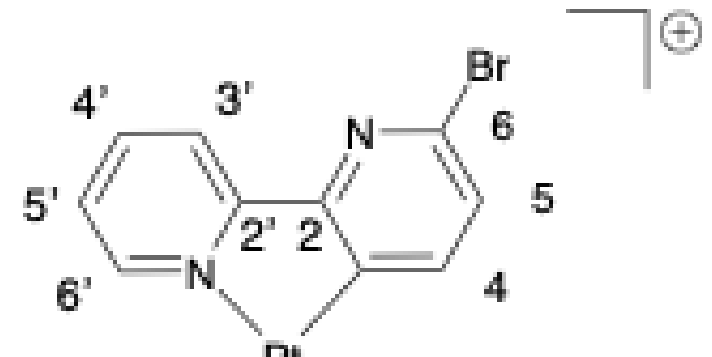

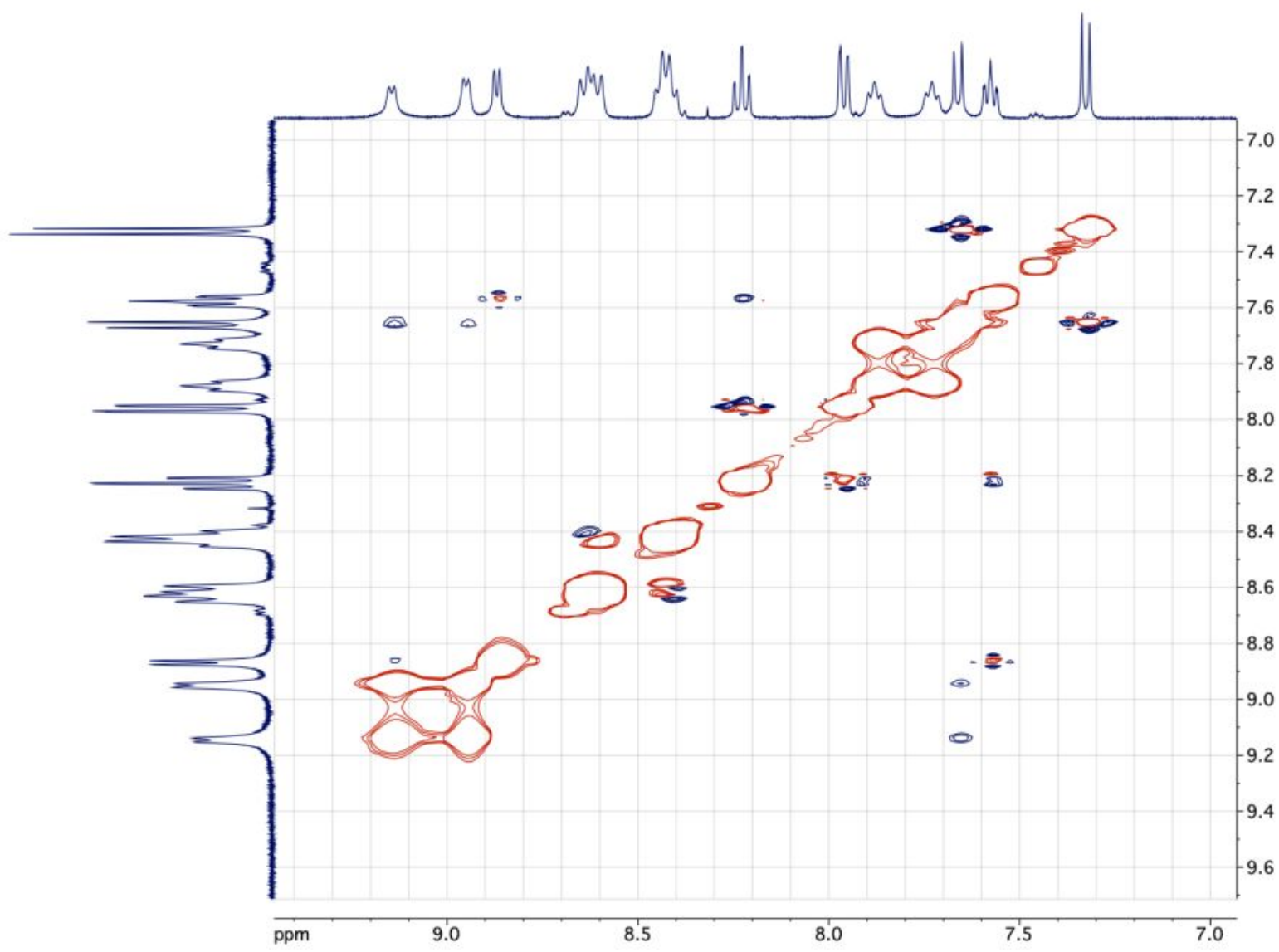

Figure S46. H-H NOESY/EXSY NMR spectrum ($CDCl_3$) of **9b** (aromatic region).

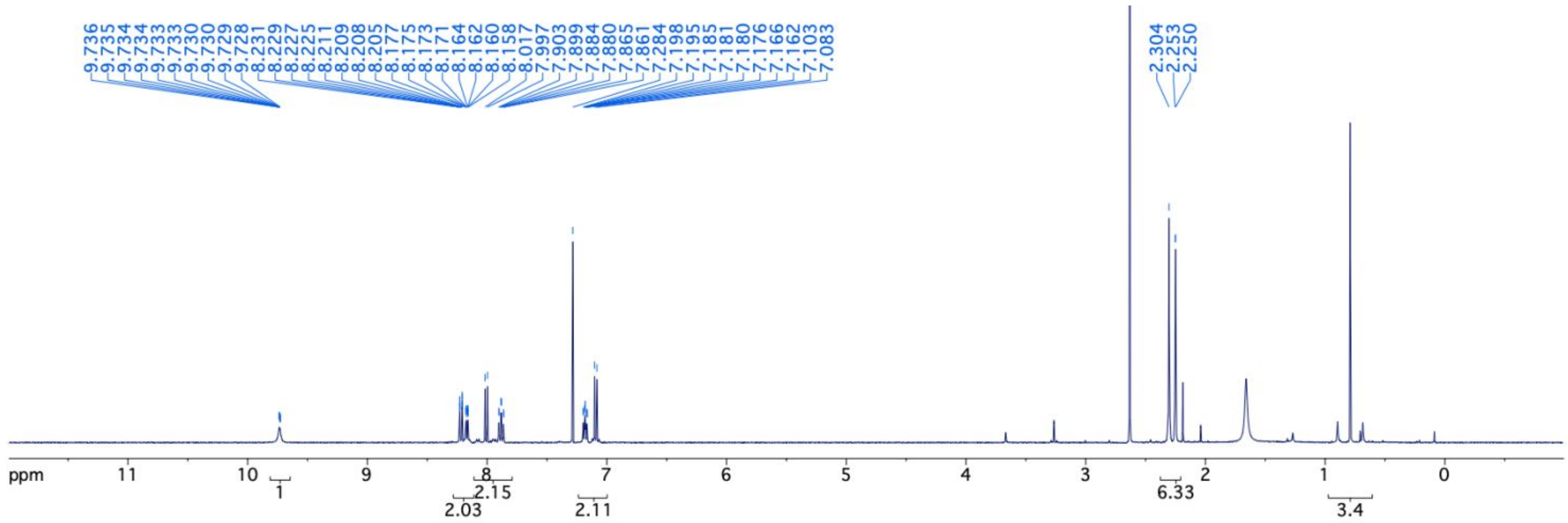

Figure S47. ^{1}H NMR spectrum ($CDCl_3$) of **10a**

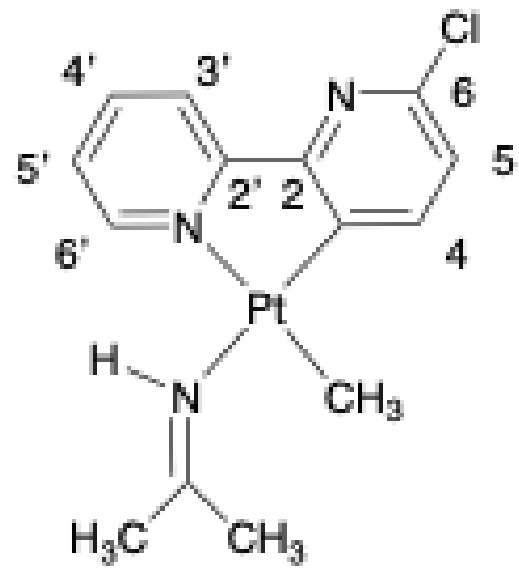

10a

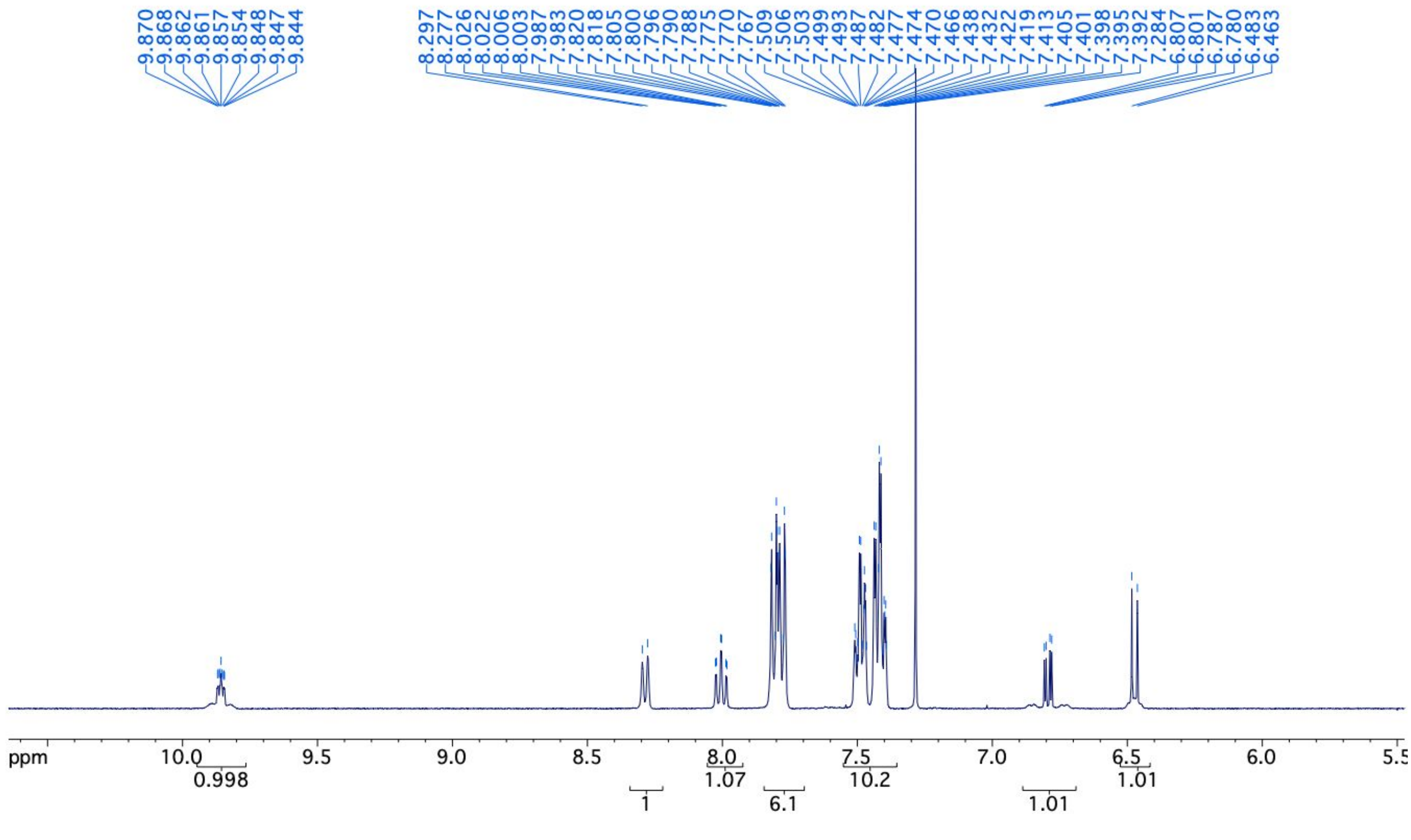

Figure S48. ^{1}H NMR spectrum (aromatic region, $CDCl_3$) of **10a**

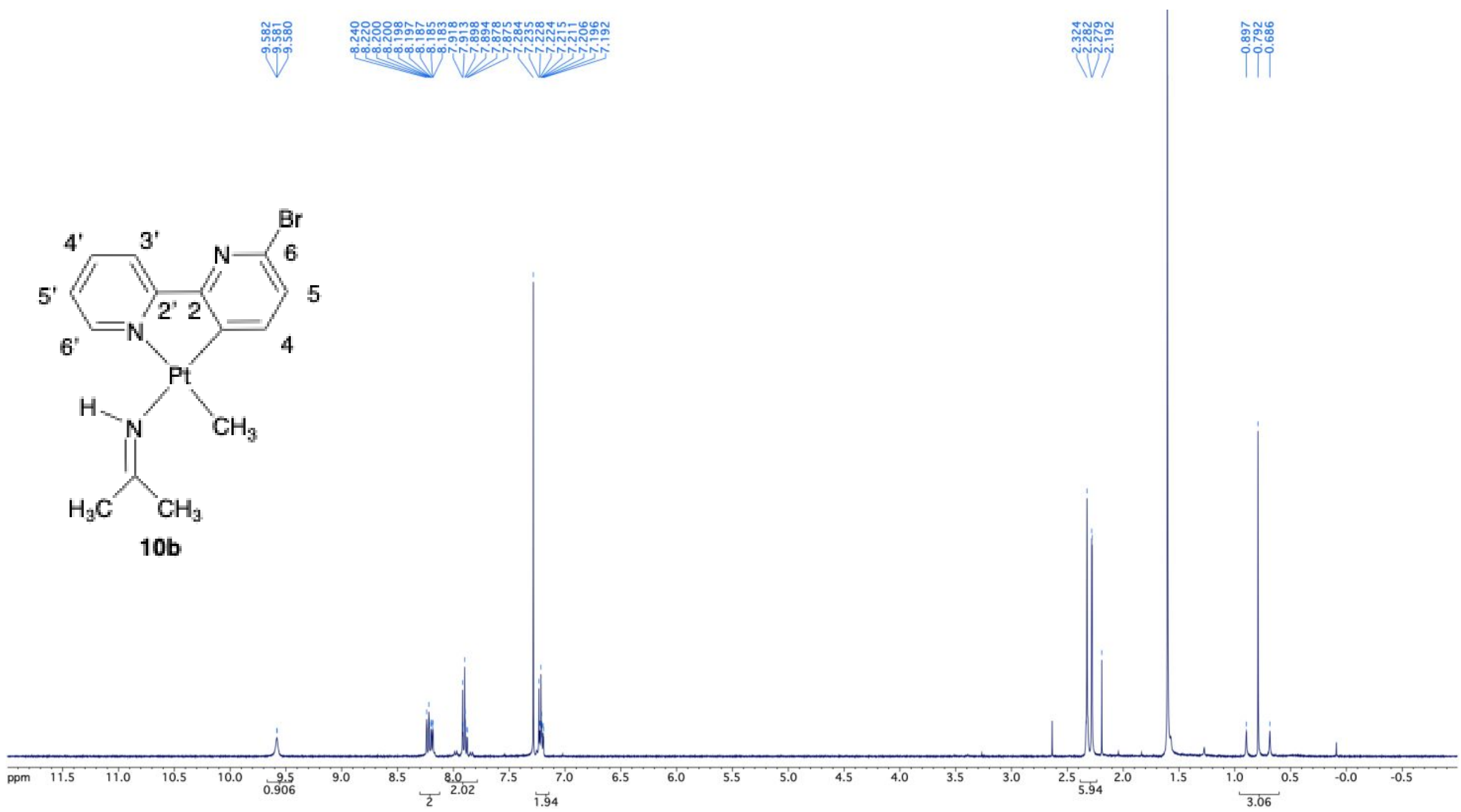

Figure S49. ^{1}H NMR spectrum ($CDCl_3$) of **10b**

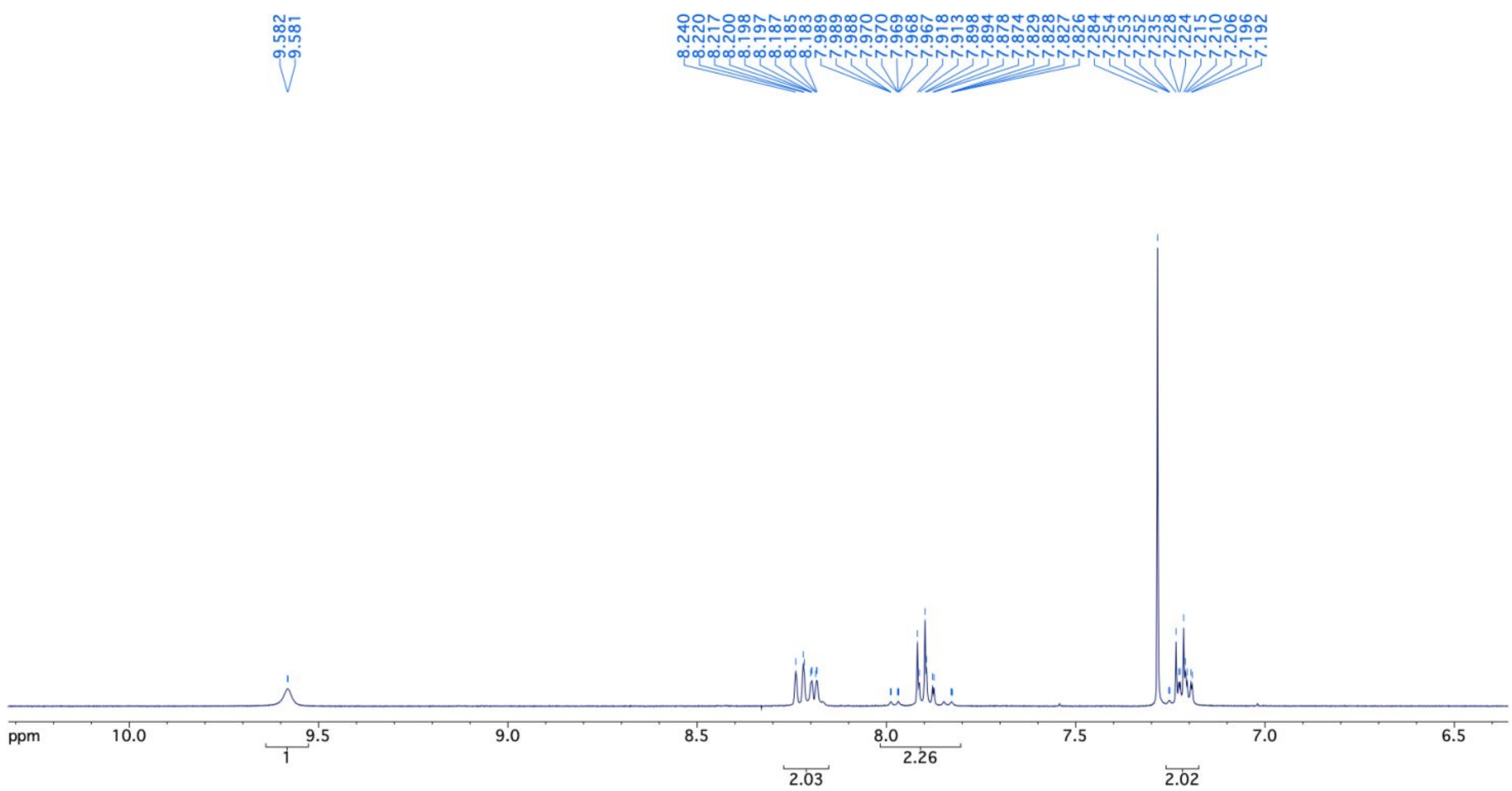

Figure S50. ^{1}H NMR spectrum (aromatic region, $CDCl_3$) of **10b**

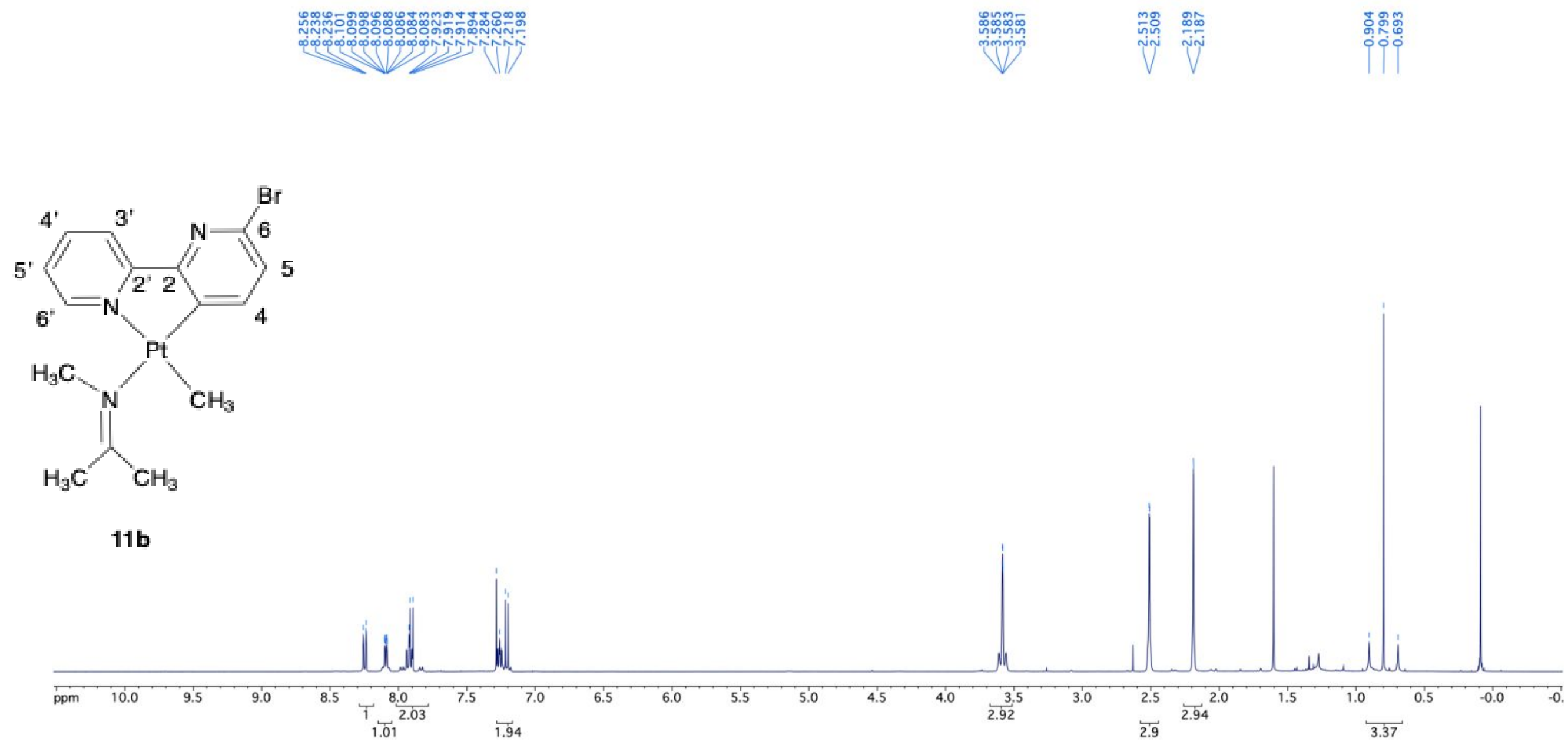

Figure S51. ^{1}H NMR spectrum ($CDCl_3$) of **11b**

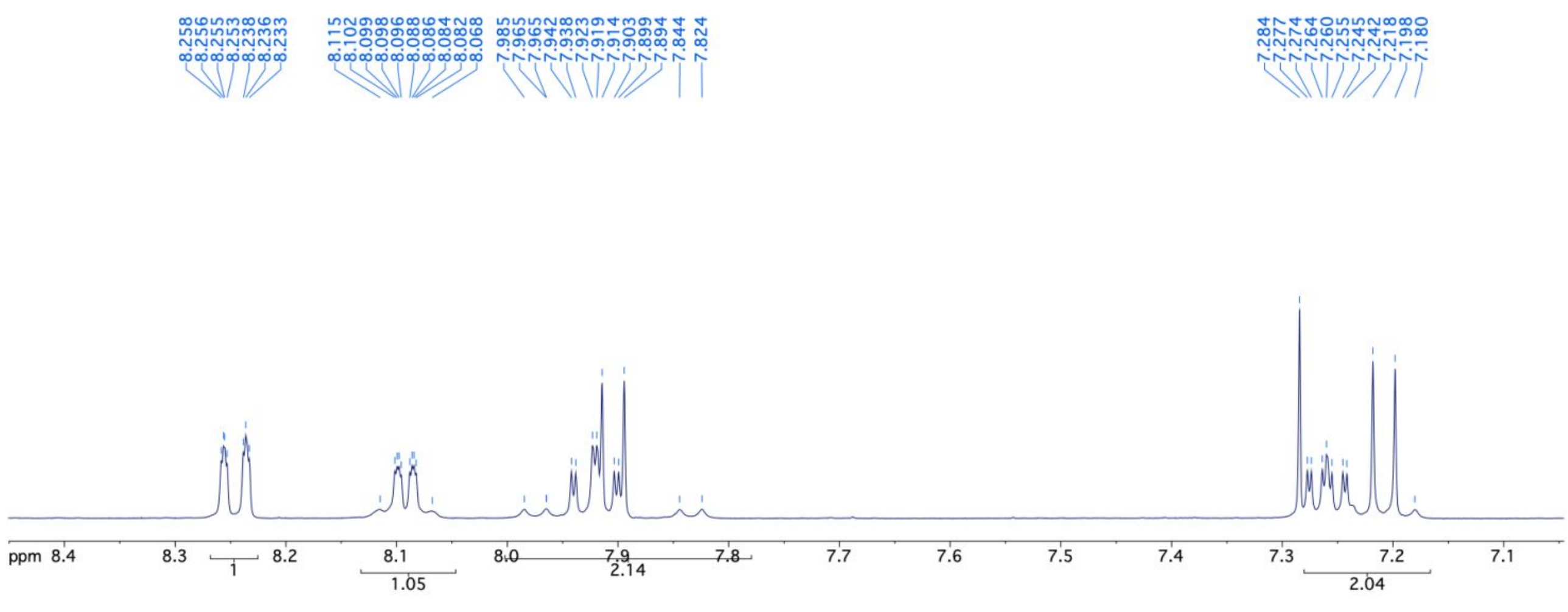

Figure S52. ^{1}H NMR spectrum (aromatic region, $CDCl_3$) of **11b**

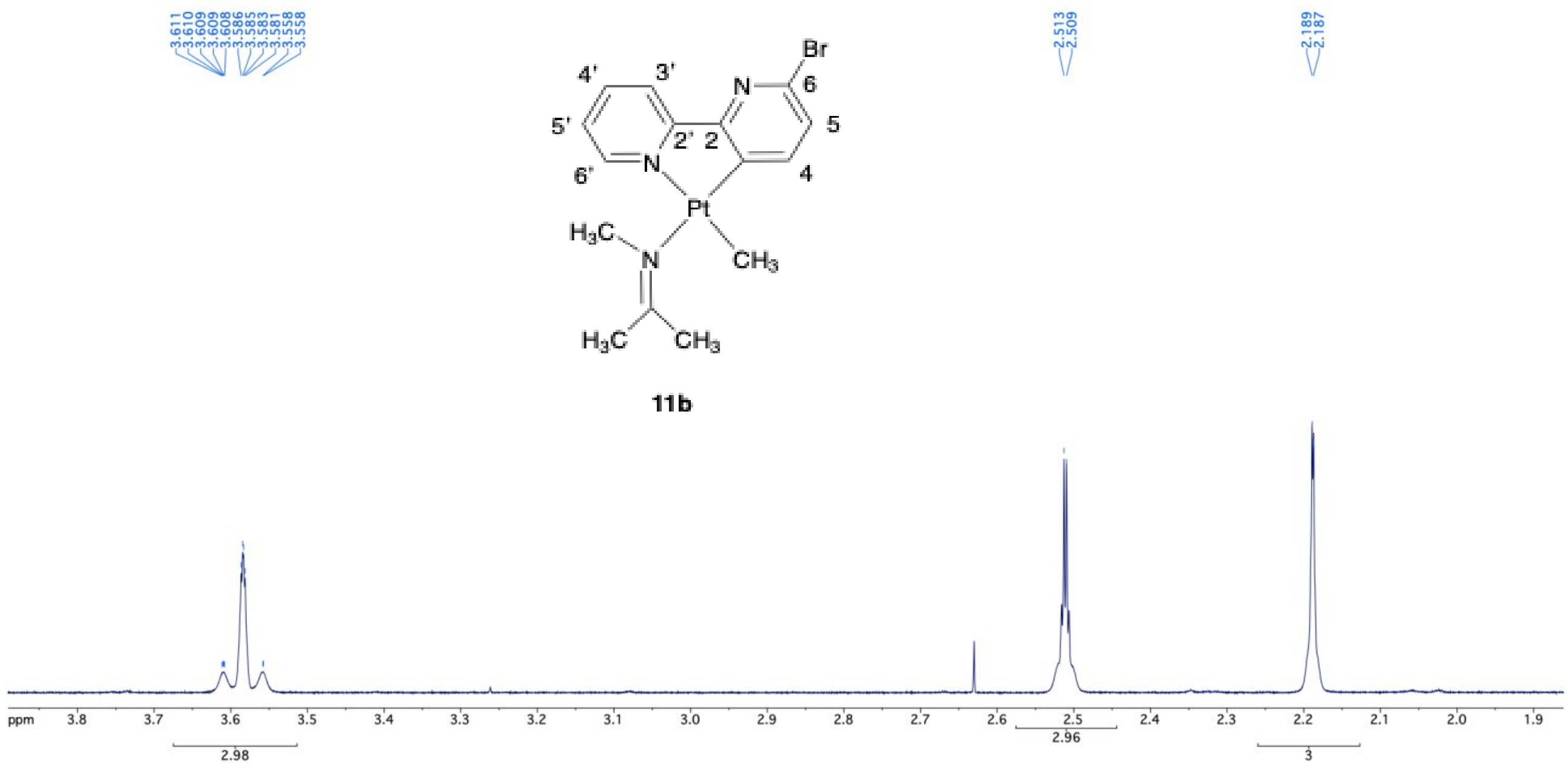

Figure S53. Expansion of the ^{1}H NMR spectrum ($CDCl_3$) of **11b**, showing the acetimine methyl signals**.**

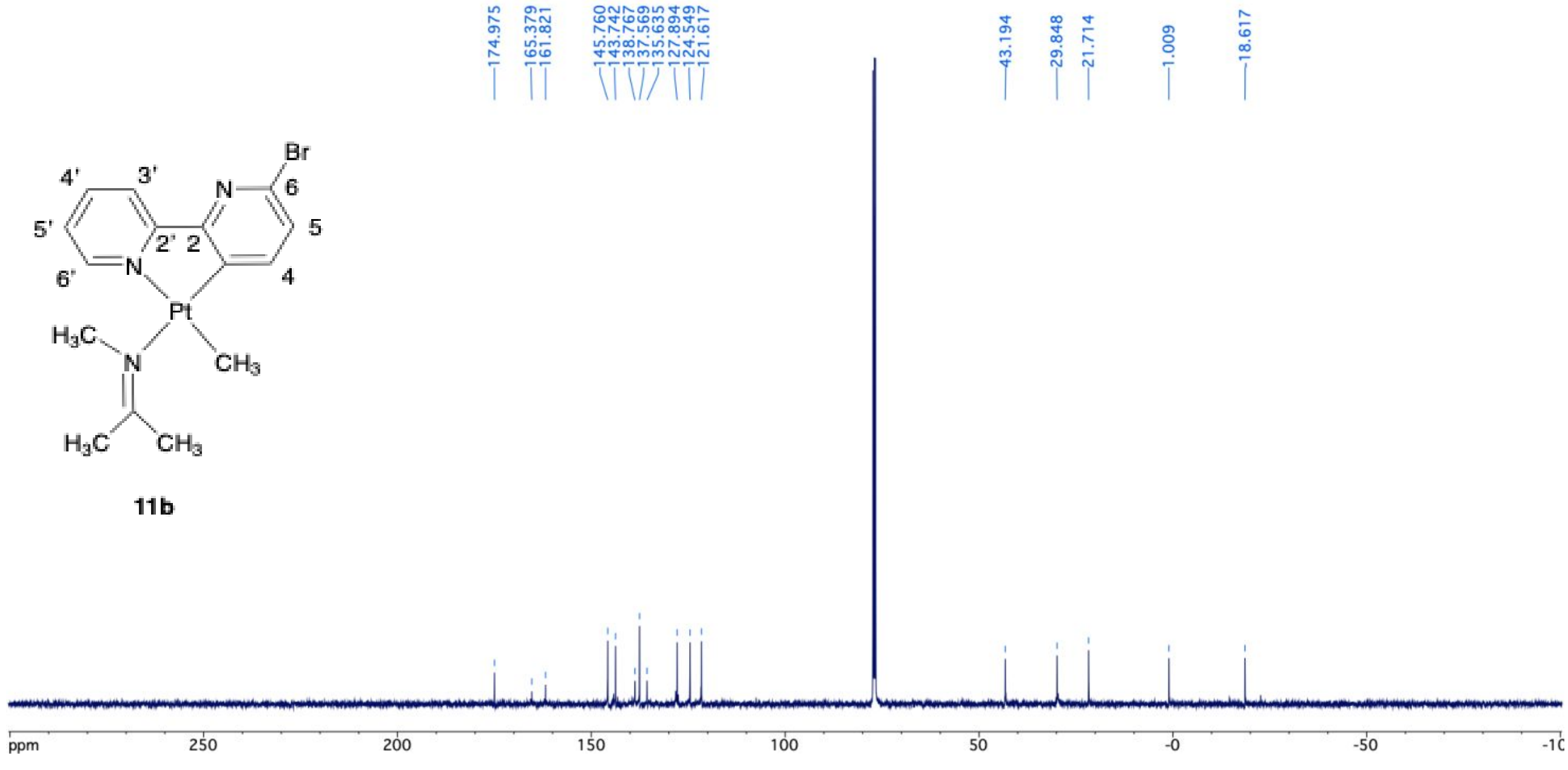

Figure S54. ^{13}C NMR spectrum ($CDCl_3$) of **11b**.

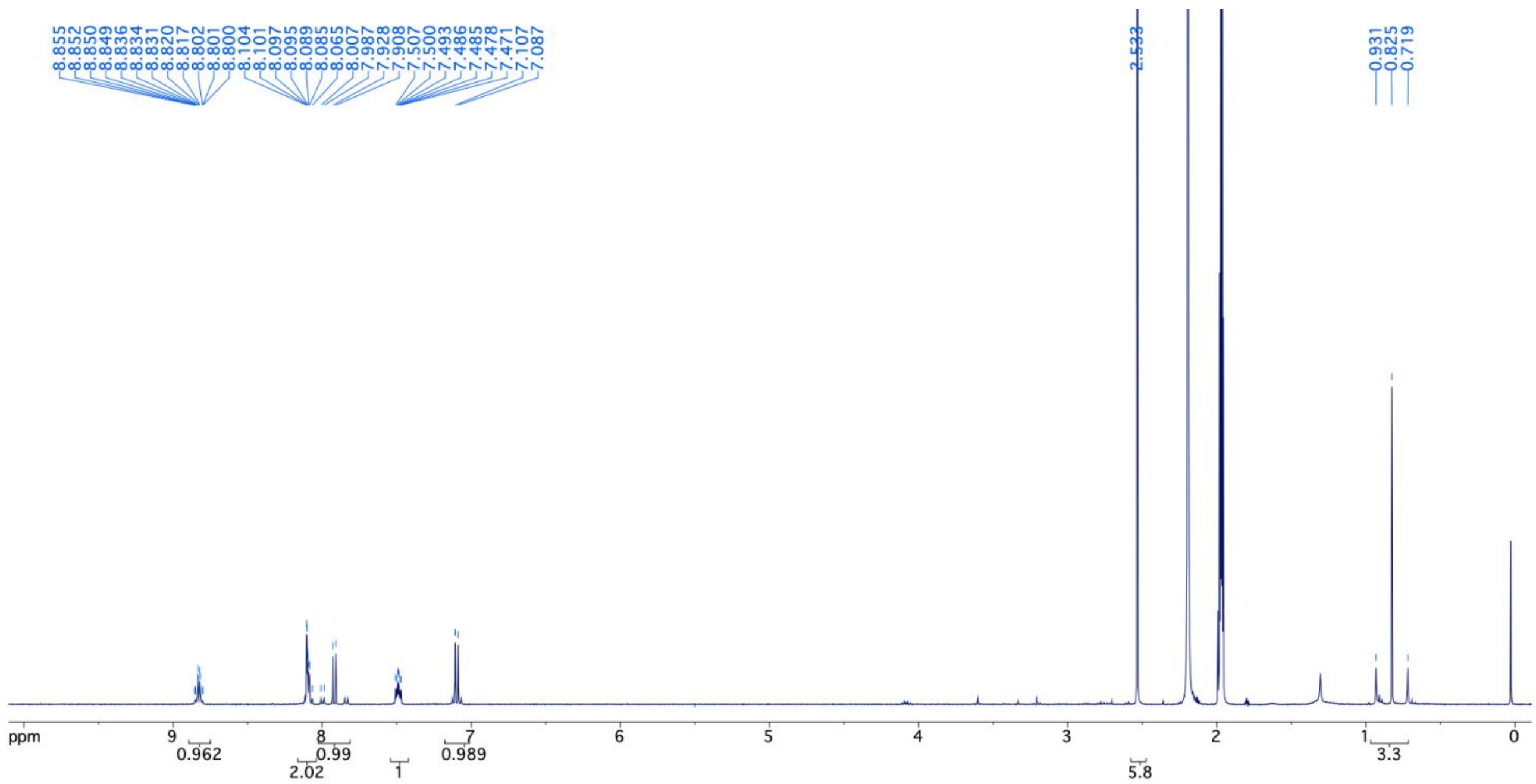

Figure S55. ^{1}H NMR spectrum (CD_3CN) of **1a**, likely corresponding to [Pt(bpy^{Br}-H)(CD_3CN)CH_3]

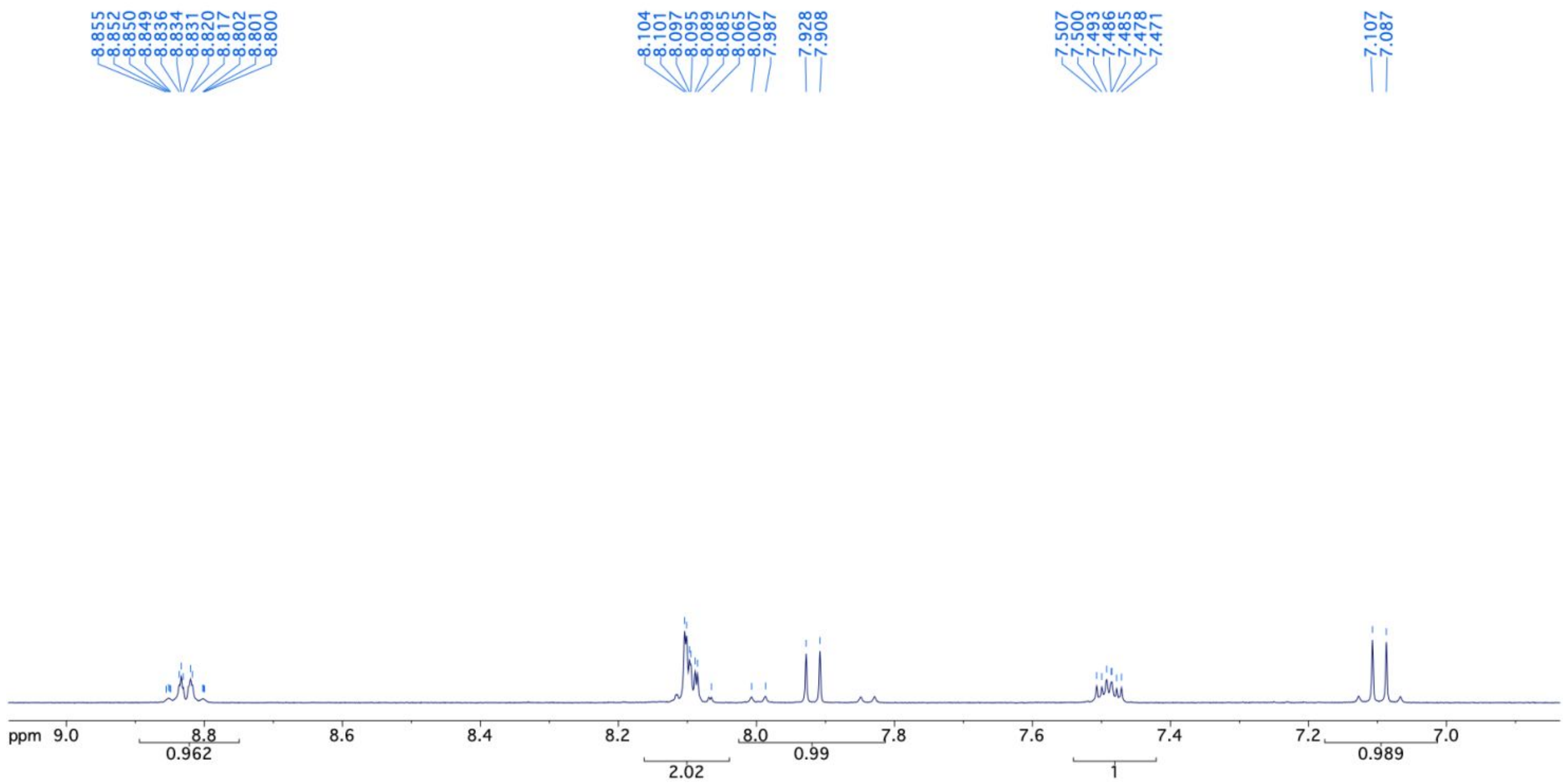

Figure S56. ^{1}H NMR spectrum (aromatic zone, CD_3CN) of **1a**, likely corresponding to [Pt(bpy^{Br}-H)(CD_3CN)CH_3]

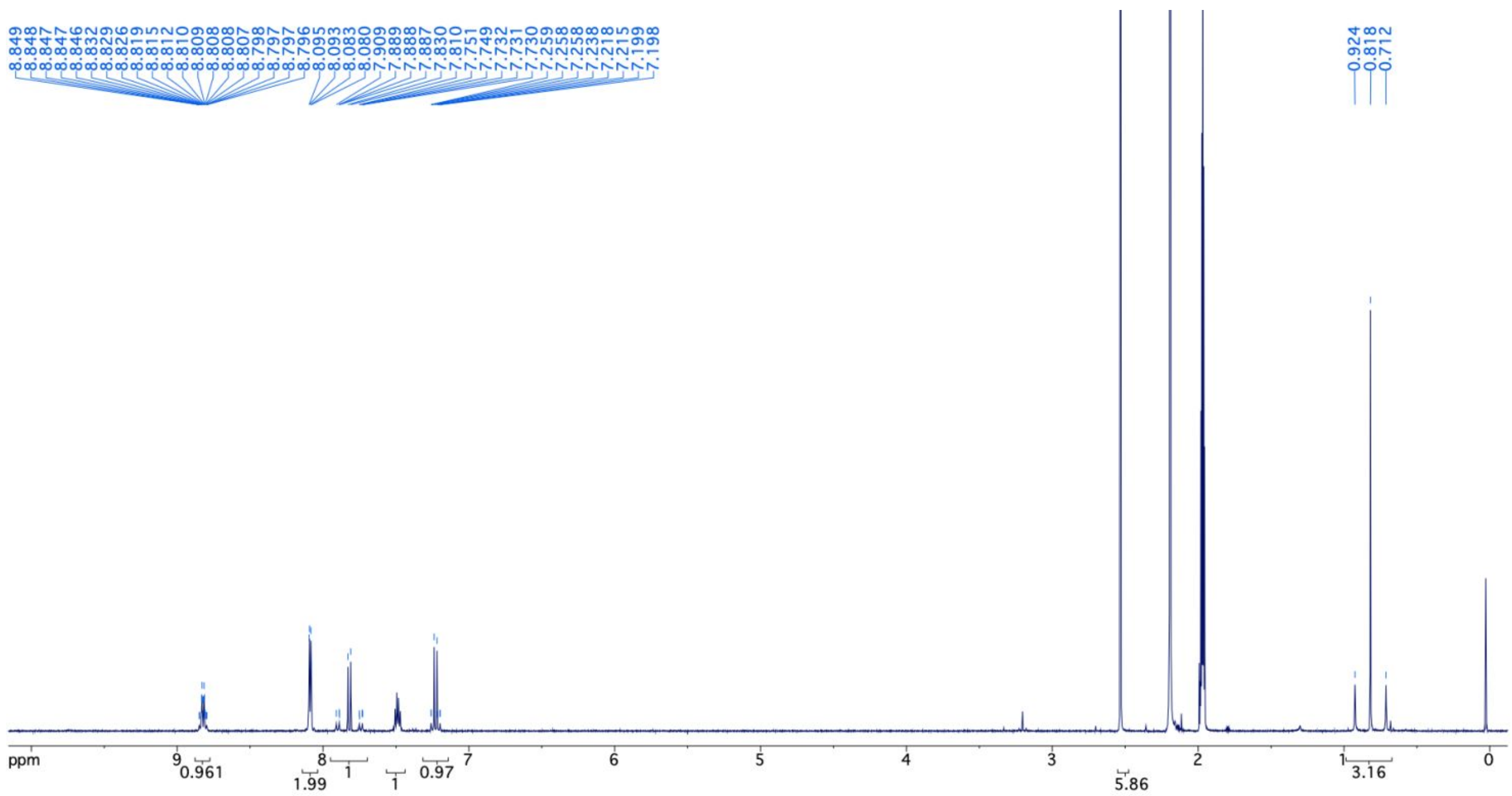

Figure S57. ^{1}H NMR spectrum (CD_3CN) of **1b**, likely corresponding to [Pt(bpy^{Br}-H)(CD_3CN)CH_3]

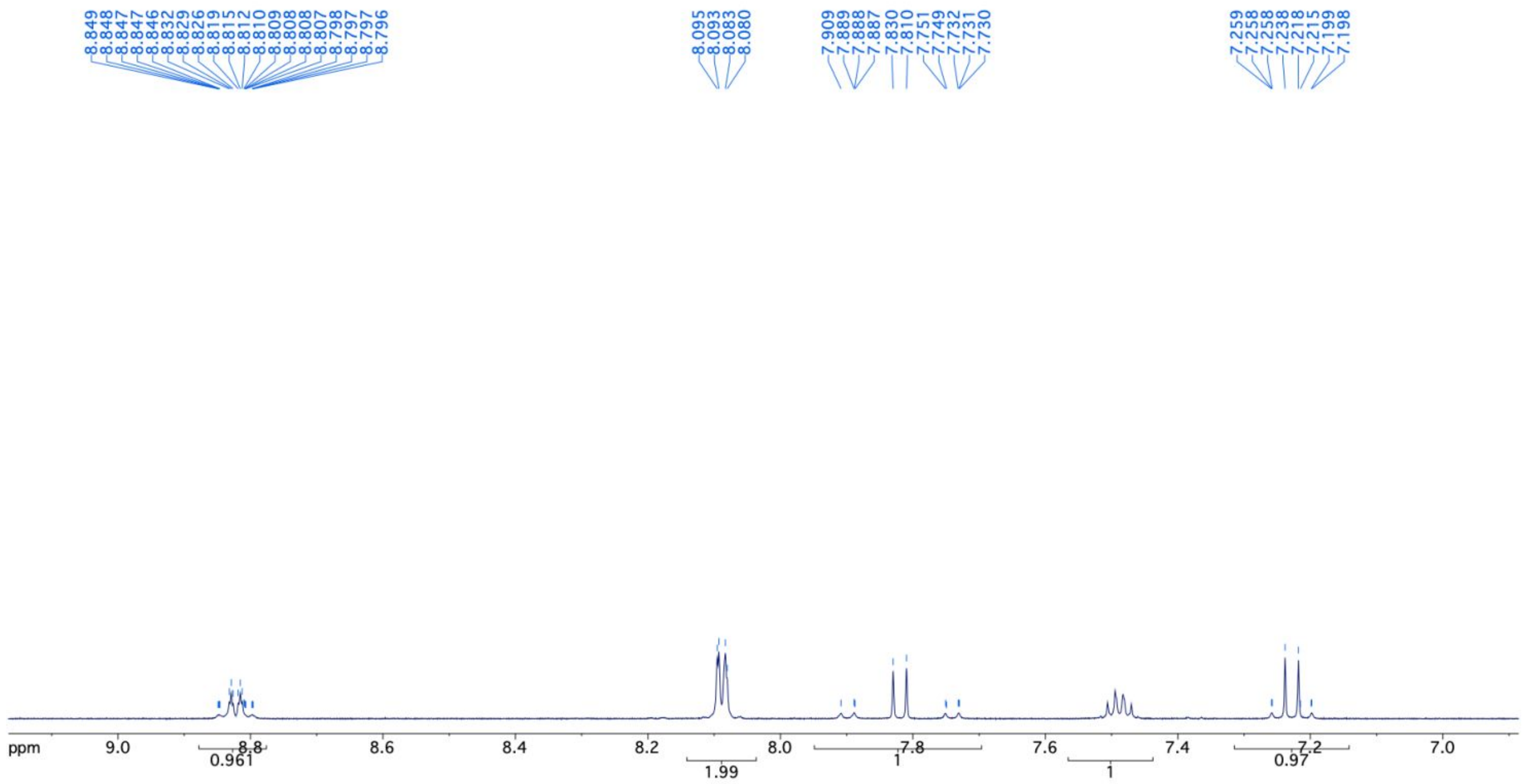

Figure S58. ^{1}H NMR spectrum (aromatic zone, CD_3CN) of **1b**, likely corresponding to [Pt(bpy^{Br}-H)(CD_3CN)CH_3]

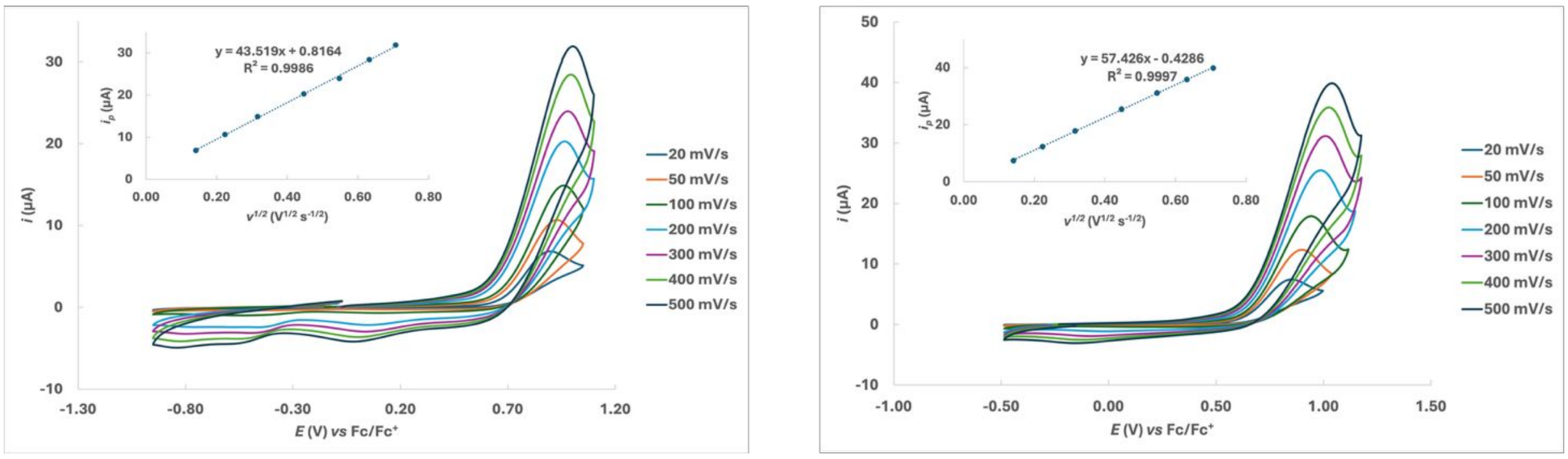

Figure S59. CV responses of **2a** (on the left) and **2b** (on the right) in CH_2Cl_2-$TEAPF_6$ 0.1 M at increasing scan rate between 20 and 500 mV s^{-1}. Inset: plot of peak current (i_p) versus potential scan rate square root ($v^{1/2}$).

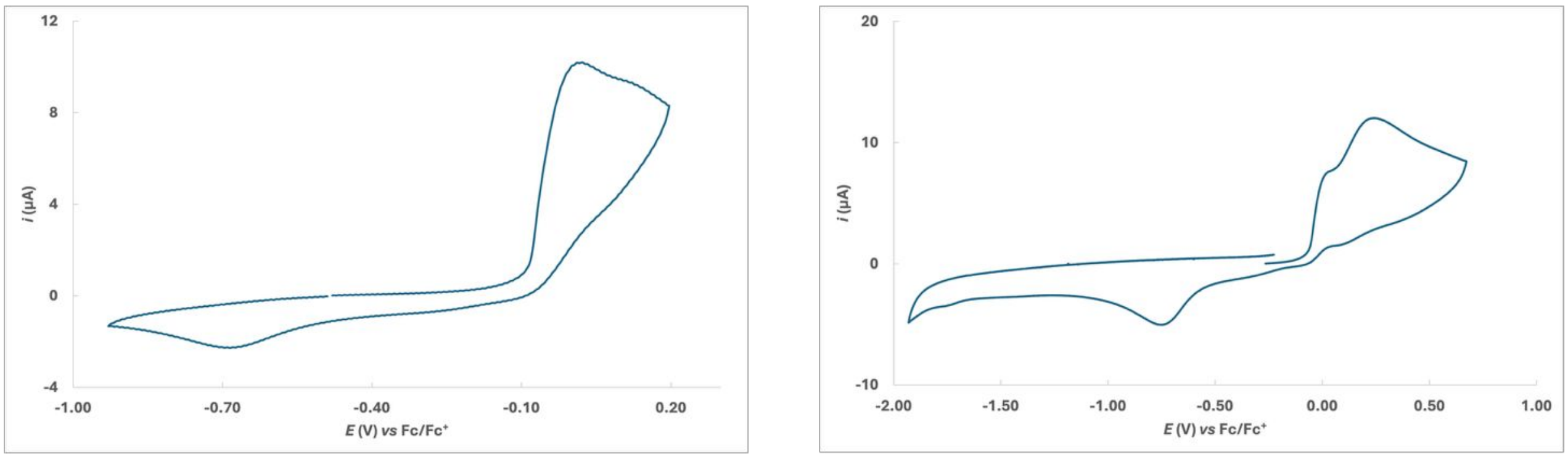

Figure S60. CV responses of **1a** (on the left) and **1b** (on the right) in CH_3CN-$TEAPF_6$ 0.1 M; v = 100 mV s^{-1}.

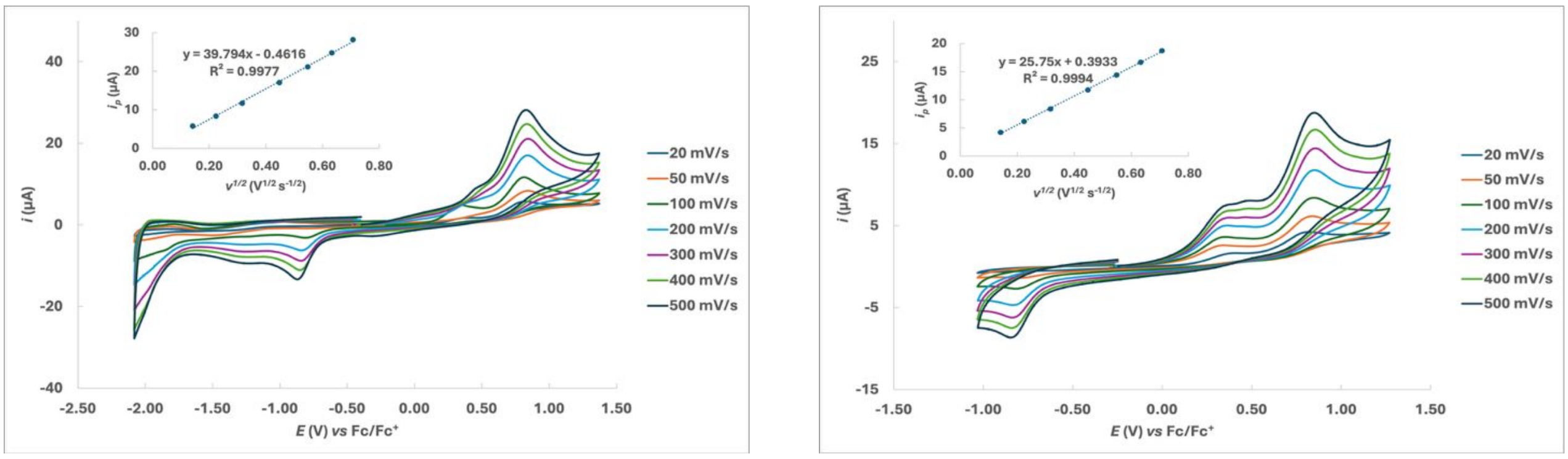

Figure S61. CV responses of **2a** (on the left) and **2b** (on the right) in CH_3CN-$TEAPF_6$ 0.1 M at increasing scan rate between 20 and 500 mV s^{-1}. Inset: plot of peak current (i_p) versus potential scan rate square root ($v^{1/2}$).

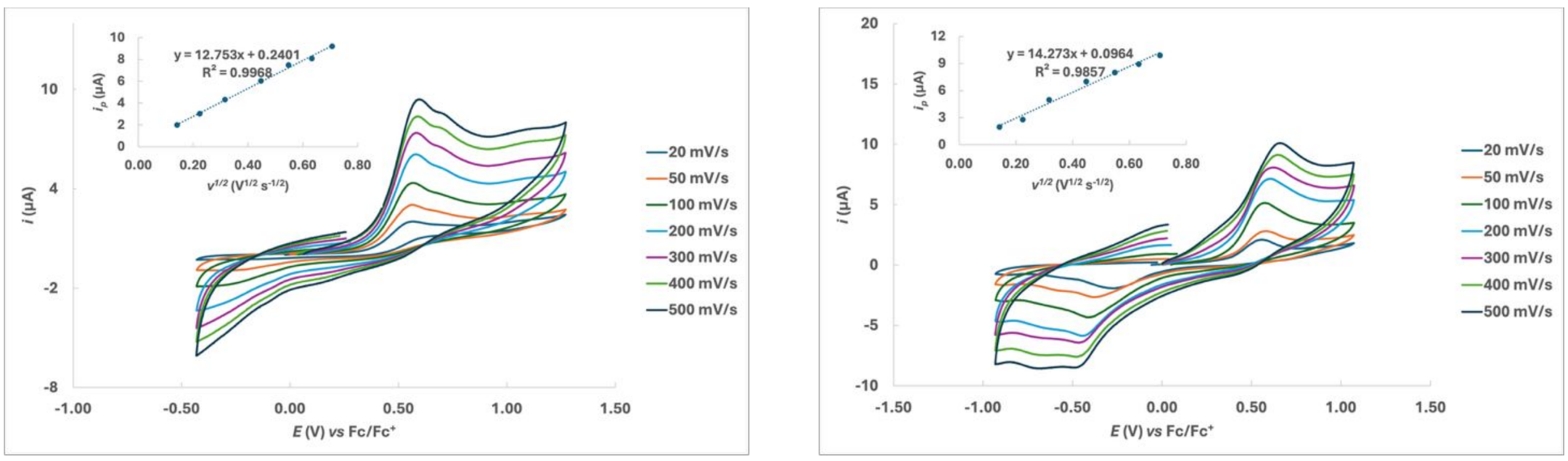

Figure **S62**. CV responses of **4a** (on the left) and **4b** (on the right) in CH_3CN-$TEAPF_6$ 0.1 M at increasing scan rate between 20 and 500 mV s^{-1}. Inset: plot of peak current (i_p) versus potential scan rate square root ($v^{1/2}$).

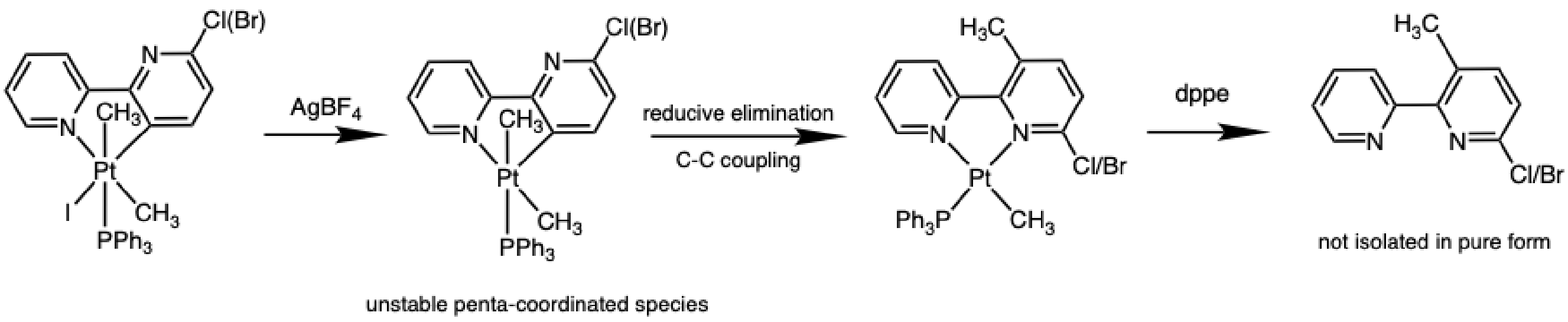

Scheme S1. Sequence of reactions proposed in the formation of 3-methyl-6-chloro-2,2'-bipyridine and 3-methyl-6-bromo-2,2'-bipyridine (not isolated in pure form)

Cartesian coordinates and free energies of all the structures optimized in the computational analysis (M062X-DFT level of theory).

Reactant **A**

Cartesian Coordinates

Pt	0.075940	-1.050911	0.041316
C	1.201944	-2.535463	0.758043
H	1.686429	-3.097204	-0.047182
H	1.986206	-2.125635	1.404243
H	0.617726	-3.250612	1.345454
C	-1.125248	-2.401485	-0.810565
H	-1.844777	-2.794187	-0.082884
H	-1.695632	-1.936098	-1.622662
H	-0.581328	-3.255377	-1.223471
O	1.425654	0.554809	1.050056
O	-1.170332	0.720196	-0.816765
S	2.662299	0.788491	0.197202
S	-2.584879	0.688054	-0.259604
C	-3.378491	2.103598	-1.005504
H	-4.365149	2.225337	-0.561830
H	-2.758133	2.982409	-0.837758
H	-3.470374	1.893195	-2.068530
C	-2.445245	1.313364	1.410374
H	-3.443689	1.431449	1.828072
H	-1.883635	0.567455	1.970529
H	-1.909278	2.261078	1.386138
C	3.566907	2.052255	1.078131
H	4.411507	2.367107	0.467488
H	2.895725	2.884635	1.283271
H	3.921073	1.604710	2.003812
C	2.105563	1.781584	-1.182267
H	2.970965	2.078864	-1.772220
H	1.438622	1.151609	-1.767939
H	1.569534	2.647277	-0.796245

Free Energy: -1305.284967 hartrees

2,2′-bipyridine

Cartesian Coordinates

N	0.298742	-0.681008	0.482845
N	-1.290547	1.507458	-0.27314
C	1.042958	-1.781993	0.515314
C	0.855666	0.442566	0.026120
C	0.004808	1.667547	0.005880
C	-2.064232	2.587829	-0.302680
C	2.365315	-1.828601	0.095097
C	2.175288	0.492251	-0.420098
C	0.560584	2.917716	0.272161
C	-1.600362	3.872575	-0.053712
H	-3.109544	2.421747	-0.539392
H	2.921086	-2.754545	0.143168
C	2.939100	-0.662831	-0.385681
H	1.609461	3.008657	0.518805
C	-0.257049	4.035592	0.243037
H	-2.277907	4.714116	-0.089984
H	3.963424	-0.652100	-0.734519
H	0.148962	5.016090	0.454709
H	2.587438	1.414021	-0.806983
H	0.560609	-2.674588	0.898560

Free Energy: -495.235428 hartrees

6-chloro-2,2′-bipyridine **(bpy^{Cl})**

Cartesian Coordinates

N	0.288583	-0.685744	0.431793
N	-1.287885	1.504452	-0.267985
C	1.021855	-1.770273	0.454647
C	0.860994	0.449979	0.014707
C	0.007950	1.671774	0.001796
C	-2.066000	2.581119	-0.294388
C	2.355672	-1.843378	0.074055
C	2.188736	0.496344	-0.394622
C	0.562157	2.922542	0.264546
C	-1.604590	3.868083	-0.050104
H	-3.112068	2.410398	-0.523726
H	2.895878	-2.777015	0.118681
C	2.940658	-0.669321	-0.363119
H	1.611613	3.018375	0.506210
C	-0.260634	4.036967	0.238820
H	-2.285421	4.707048	-0.083349
H	3.974097	-0.663850	-0.682956
H	0.142588	5.019201	0.447236
Cl	0.236747	-3.224057	1.015031
H	2.621269	1.419756	-0.751975

Free Energy: -954.854178 hartrees

6-bromo-2,2′-bipyridine **(bpy^{Br})**

Cartesian Coordinates

N	0.289343	-0.684679	0.429655
N	-1.284617	1.507012	-0.281624
C	1.021270	-1.767628	0.451100
C	0.861728	0.452018	0.011682
C	0.008899	1.673448	-0.000707
C	-2.063385	2.583294	-0.306078
C	2.354401	-1.844533	0.069256
C	2.189156	0.496552	-0.398367
C	0.560875	2.922567	0.273643
C	-1.604470	3.868721	-0.049211
H	-3.107545	2.413626	-0.544414
H	2.896375	-2.777070	0.111652
C	2.939650	-0.670099	-0.368098
H	1.608772	3.017122	0.522452
C	-0.262706	4.036583	0.250004
H	-2.285686	4.707455	-0.080850
H	3.972727	-0.665614	-0.689330
H	0.138315	5.017660	0.467818
H	2.622409	1.419607	-0.755923
Br	0.162388	-3.361942	1.065638

Free Energy: -3068.848402 hartrees

DMSO

Cartesian Coordinates

S	-0.380063	0.242633	0.857728
O	0.336062	1.261164	0.020527
C	0.255396	-1.353769	0.345880
H	-0.309764	-2.142217	0.840529
H	0.174386	-1.431022	-0.737615
H	1.297805	-1.393739	0.654343
C	-2.015406	0.075168	0.142256
H	-1.911832	-0.118834	-0.924587
H	-2.545466	-0.735081	0.640548
H	-2.533120	1.016998	0.310292

Free Energy: -553.149912 hartrees

Complex **B1**

Cartesian Coordinates

Pt	1.496931	4.215113	10.963142
N	0.624414	4.289039	8.770378
N	1.507459	6.337973	10.340044
C	0.193414	3.298015	8.008938
C	0.912652	5.463776	8.170109
C	1.251767	6.611248	9.053004
C	1.780675	7.337640	11.177264
C	0.096996	3.356524	6.624846
C	0.862545	5.608140	6.791605
C	1.257745	7.919457	8.579750
C	1.813899	8.662123	10.777485
H	1.978824	7.057631	12.202881
H	-0.254594	2.500342	6.069637
C	0.464579	4.534997	6.010389
H	1.138458	6.539129	6.322857
H	1.021321	8.137408	7.550026
C	1.545382	8.955357	9.451426
H	2.038265	9.436877	11.496193
H	0.426428	4.625589	4.933485
H	1.549814	9.977173	9.097136
C	2.369890	4.562092	12.733789
H	3.254335	5.192389	12.583335
H	1.675690	5.113861	13.379893
H	2.687641	3.673374	13.279533
C	1.510498	2.243124	11.412946
H	0.496568	1.886920	11.627055
H	1.874862	1.662758	10.558551
H	2.134422	1.986304	12.271846
Cl	-0.322577	1.847128	8.786160

Free Energy: -1153.852746 hartrees

Complex **C1**

Cartesian Coordinates

Pt	1.480576	4.221580	10.968545
N	0.549615	4.307963	8.785486
N	1.453617	6.344803	10.360908
C	0.093069	3.328974	8.026111
C	0.913101	5.459208	8.180752
C	1.246740	6.609097	9.063123
C	1.707937	7.348408	11.198873
Br	-0.639771	1.805265	8.876366
C	0.061492	3.363489	6.638411
C	0.930210	5.579950	6.799183
C	1.287306	7.911862	8.577799
C	1.770554	8.668861	10.788451

H	1.868244	7.074692	12.232994
H	-0.312526	2.520143	6.078462
C	0.519965	4.509498	6.020746
H	1.266643	6.489882	6.327951
H	1.090857	8.121677	7.537882
C	1.555943	8.952413	9.450519
H	1.977147	9.447958	11.507806
H	0.536191	4.578814	4.941619
H	1.586846	9.970724	9.087502
C	2.392111	4.562286	12.720357
H	3.259733	5.211961	12.556286
H	1.702424	5.091837	13.389308
H	2.739263	3.671979	13.245246
C	1.571575	2.243859	11.380095
H	0.604708	1.869426	11.735011
H	1.815326	1.680642	10.473004
H	2.317902	1.985752	12.135103

Free Energy: -3267.844986 hartrees

Transition State **BTS1-2**
Imaginary Frequency at -34 cm^{-1}
Cartesian Coordinates

Pt	-0.0599	-1.1576	0.0205
N	0.9358	1.4538	-0.5659
N	-1.8342	0.2617	0.0319
C	2.2074	1.4149	-0.2182
C	0.0338	1.6839	0.3996
C	-1.3981	1.5276	-0.0060
C	-3.1099	0.0110	-0.2553
C	2.6766	1.5847	1.0756
C	0.3870	1.8187	1.7348
C	-2.2263	2.5905	-0.3167
C	-4.0091	1.0172	-0.5720
H	-3.4114	-1.0285	-0.2311
H	3.7341	1.5395	1.2887
C	1.7331	1.7885	2.0681
H	-0.3777	1.9486	2.4877
H	-1.8338	3.5978	-0.3249
C	-3.5579	2.3264	-0.6084
H	-5.0382	0.7704	-0.7898
H	2.0455	1.9002	3.0976
H	-4.2327	3.1346	-0.8562
C	-0.9735	-2.8778	0.4315
H	-2.0280	-2.6831	0.6449
H	-0.9022	-3.5604	-0.4182
H	-0.5232	-3.3622	1.2995
C	1.6782	-2.1547	-0.1104
H	1.9414	-2.3260	-1.1606
H	2.4755	-1.5425	0.3326
H	1.6864	-3.1242	0.3939
Cl	3.3398	1.0962	-1.4921

Free Energy: -1153.832969 hartrees

Complex **B2**
Cartesian Coordinates

Pt	-1.6186	-0.6302	-0.2166
N	2.7542	0.3700	-0.2034
N	-0.5998	1.3822	0.1621
C	3.5646	-0.6424	-0.0087
C	1.5994	0.3854	0.4726
C	0.7257	1.5670	0.2403
C	-1.3811	2.4549	0.0012
C	3.3216	-1.6996	0.8585
C	1.2567	-0.6080	1.3811
C	1.3022	2.8273	0.1294
C	-0.8862	3.7431	-0.0965
H	-2.4437	2.2608	-0.0627
H	4.0368	-2.5009	0.9684
C	2.1368	-1.6643	1.5709
H	0.3287	-0.5459	1.9304
H	2.3769	2.9209	0.1872
C	0.4859	3.9311	-0.0396
H	-1.5669	4.5723	-0.2245
H	1.9028	-2.4525	2.2739
H	0.9132	4.9216	-0.1203
C	-3.2663	-0.1288	0.7766
H	-3.0105	0.5661	1.5821
H	-3.9665	0.3655	0.0955
H	-3.7667	-0.9977	1.2063
C	-2.4634	-2.3803	-0.7144
H	-3.4180	-2.2550	-1.2345
H	-1.7861	-2.9224	-1.3874
H	-2.6459	-3.0190	0.1555
Cl	5.0497	-0.6316	-0.9185

Free Energy: -1153.843496 hartrees

Transition State **BTS2-3**

Imaginary Frequency at -256 cm^{-1}

Cartesian Coordinates

Pt	-1.4026	-0.6481	0.0254
N	2.6735	0.5263	-0.0429
N	-0.7707	1.4908	0.0342
C	3.3847	-0.5748	-0.0529
C	1.3566	0.4171	0.1418
C	0.5579	1.6619	0.1056
C	-1.5689	2.5542	-0.0053
C	2.8632	-1.8527	0.1083
C	0.7092	-0.8127	0.3187
C	1.1296	2.9259	0.1317
C	-1.0748	3.8481	0.0222
H	-2.6307	2.3526	-0.0665
H	3.5067	-2.7195	0.0853
C	1.4993	-1.9578	0.3093
H	-0.3623	-0.8989	1.0762
H	2.2044	3.0195	0.1835
C	0.2981	4.0328	0.0905
H	-1.7564	4.6855	-0.0115
H	1.0510	-2.9314	0.4570
H	0.7171	5.0299	0.1120
C	-3.3205	-0.2789	-0.6488
H	-3.8897	0.2171	0.1436
H	-3.2680	0.4101	-1.4984
H	-3.8698	-1.1685	-0.9568
C	-1.8894	-2.6175	0.0410
H	-1.9353	-2.9884	-0.9866
H	-1.1620	-3.2213	0.5873
H	-2.8698	-2.7801	0.4920
Cl	5.0986	-0.3912	-0.2887

Free Energy: -1153.8187751 hartrees

Complex **B3**

Cartesian Coordinates

Pt	-1.4133	-0.6482	-0.0105
N	2.6989	0.5213	0.0090
N	-0.7566	1.5014	0.0024
C	3.4070	-0.5777	-0.0008
C	1.3643	0.3883	0.0134
C	0.5786	1.6477	0.0117
C	-1.5435	2.5742	-0.0113
C	2.8718	-1.8592	-0.0065
C	0.6733	-0.8325	0.0115
C	1.1596	2.9098	0.0114
C	-1.0360	3.8622	-0.0133
H	-2.6092	2.3844	-0.0236
H	3.5181	-2.7251	-0.0185
C	1.4897	-1.9651	0.0013
H	-1.4958	-0.7771	1.4681
H	2.2364	2.9919	0.0173
C	0.3415	4.0269	-0.0016
H	-1.7081	4.7078	-0.0261
H	1.0487	-2.9562	-0.0061
H	0.7745	5.0186	-0.0049
C	-3.4643	-0.2370	-0.0259
H	-3.7353	0.5148	0.7212
H	-3.7217	0.1742	-1.0101
H	-4.0926	-1.1124	0.1452
C	-1.7730	-2.6335	-0.0206
H	-1.5891	-2.9761	-1.0444
H	-1.0914	-3.1643	0.6443
H	-2.7964	-2.8819	0.2542
Cl	5.1465	-0.3896	-0.0115

Free Energy: -1153.825474 hartrees

Transition State **BTS3-4**

Imaginary Frequency at -486 cm^{-1}

Cartesian Coordinates

Pt	-1.4133	-0.6482	-0.0105
N	2.6989	0.5213	0.0090
N	-0.7566	1.5014	0.0024
C	3.4070	-0.5777	-0.0008
C	1.3643	0.3883	0.0134
C	0.5786	1.6477	0.0117
C	-1.5435	2.5742	-0.0113
C	2.8718	-1.8592	-0.0065
C	0.6733	-0.8325	0.0115
C	1.1596	2.9098	0.0114
C	-1.0360	3.8622	-0.0133
H	-2.6092	2.3844	-0.0236
H	3.5181	-2.7251	-0.0185
C	1.4897	-1.9651	0.0013
H	-1.4958	-0.7771	1.4681
H	2.2364	2.9919	0.0173
C	0.3415	4.0269	-0.0016
H	-1.7081	4.7078	-0.0261

H	1.0487	-2.9562	-0.0061
H	0.7745	5.0186	-0.0049
C	-3.4643	-0.2370	-0.0259
H	-3.7353	0.5148	0.7212
H	-3.7217	0.1742	-1.0101
H	-4.0926	-1.1124	0.1452
C	-1.7730	-2.6335	-0.0206
H	-1.5891	-2.9761	-1.0444
H	-1.0914	-3.1643	0.6443
H	-2.7964	-2.8819	0.2542
Cl	5.1465	-0.3896	-0.0115

Free Energy: -1153.821008 hartrees

Complex **B4**
Cartesian Coordinates

Pt	0.6546	-0.4642	-0.2747
N	-3.3836	0.3729	0.1654
N	-0.0350	1.6083	-0.1157
C	-3.9877	-0.7866	0.1908
C	-2.0537	0.3717	0.0241
C	-1.3699	1.6806	0.0119
C	0.6921	2.7221	-0.1330
C	-3.3383	-2.0094	0.0828
C	-1.2552	-0.7845	-0.1031
C	-2.0185	2.9025	0.1274
C	0.1159	3.9772	-0.0233
H	1.7616	2.5853	-0.2359
H	-3.8974	-2.9337	0.1161
C	-1.9618	-1.9920	-0.0650
H	-3.0937	2.9213	0.2299
C	-1.2631	4.0634	0.1090
H	0.7380	4.8603	-0.0411
H	-1.4353	-2.9340	-0.1483
H	-1.7451	5.0281	0.1979
C	1.1188	-2.4343	-0.4289
H	0.8753	-2.9786	0.4899
H	0.5669	-2.9117	-1.2438
H	2.1833	-2.5964	-0.6318
Cl	-5.7267	-0.7640	0.3794
O	2.7981	0.2495	-0.4088
S	3.9529	-0.6339	0.0543
C	3.6030	-0.9990	1.7679
H	2.7427	-1.6656	1.7758
H	4.4704	-1.4915	2.2044
H	3.3740	-0.0660	2.2808
C	5.2884	0.5254	0.2857
H	5.5486	0.9086	-0.6982
H	4.9476	1.3279	0.9374
H	6.1358	-0.0047	0.7170

Free Energy: -1666.556975 hartrees

CH_4
Cartesian Coordinates

C	0.136266	-0.199180	0.000000
H	0.498957	-1.225080	0.000000
H	0.498963	0.313735	0.888467
H	0.498963	0.313735	-0.888467
H	-0.951851	-0.199108	-0.000000

Free Energy: -40.475348 hartrees

Transition State **CTS1-2**
Imaginary Frequency at -39 cm^{-1}
Cartesian Coordinates

Pt	-0.5193	-1.1694	0.0451
N	0.8367	1.2594	-0.2549
N	-2.1193	0.4421	-0.0479
C	2.0347	1.0448	0.2513
C	-0.1475	1.5966	0.5931
C	-1.5208	1.6390	-0.0009
C	-3.3682	0.3623	-0.5018
Br	3.3933	0.5604	-0.9854
C	2.3483	1.1280	1.5990
C	0.0407	1.6541	1.9668
C	-2.1547	2.8036	-0.3929
C	-4.0800	1.4803	-0.9080
H	-3.8015	-0.6291	-0.5420
H	3.3525	0.9410	1.9482
C	1.3152	1.4371	2.4686
H	-0.7930	1.8729	2.6193
H	-1.6370	3.7504	-0.3298
C	-3.4600	2.7181	-0.8591

H	-5.0953	1.3729	-1.2615
H	1.5006	1.4869	3.5331
H	-3.9856	3.6091	-1.1755
C	-1.6684	-2.7784	0.2815
H	-2.7090	-2.4629	0.3963
H	-1.5898	-3.4373	-0.5860
H	-1.3729	-3.3430	1.1676
C	1.0900	-2.3702	0.0390
H	1.5221	-2.4176	-0.9675
H	1.8569	-1.9499	0.7035
H	0.8945	-3.3947	0.3652

Free Energy: -3267.826902 hartrees

Intermediate **C2**

Cartesian Coordinates

Pt	-2.0085	-0.6902	-0.2549
N	2.2705	0.5525	0.0333
N	-1.1471	1.3865	0.1553
C	3.1110	-0.4177	0.2947
C	1.0727	0.5194	0.6291
C	0.1570	1.6494	0.3179
C	-1.9773	2.4079	-0.0760
Br	4.7978	-0.3404	-0.5945
C	2.8614	-1.4767	1.1569
C	0.7147	-0.4806	1.5246
C	0.6671	2.9388	0.2212
C	-1.5514	3.7213	-0.1647
H	-3.0202	2.1496	-0.2055
H	3.6016	-2.2439	1.3271
C	1.6295	-1.4902	1.7880
H	-0.2506	-0.4572	2.0085
H	1.7285	3.0963	0.3473
C	-0.1990	3.9899	-0.0226
H	-2.2684	4.5075	-0.3522
H	1.3866	-2.2819	2.4841
H	0.1749	5.0025	-0.0953
C	-3.7007	-0.2833	0.7072
H	-3.5057	0.4477	1.4974
H	-4.4276	0.1415	0.0077
H	-4.1412	-1.1759	1.1539
C	-2.7456	-2.4882	-0.7492
H	-3.7115	-2.4227	-1.2596
H	-2.0451	-2.9931	-1.4272
H	-2.8817	-3.1341	0.1242

Free Energy: -3267.837849 hartrees

Transition State **CTS2-3**

Imaginary Frequency at -316 cm^{-1}

Cartesian Coordinates

Pt	-1.8688	-0.6738	0.0020
N	2.2073	0.6176	0.0357
N	-1.2635	1.4733	0.0436
C	2.9593	-0.4550	0.0559
C	0.8973	0.4692	0.2431
C	0.0553	1.6847	0.1673
C	-2.0876	2.5153	-0.0325
Br	4.8229	-0.1902	-0.2346
C	2.4942	-1.7470	0.2693
C	0.3129	-0.7787	0.4786
C	0.5903	2.9641	0.2098
C	-1.6318	3.8228	0.0085
H	-3.1403	2.2848	-0.1351
H	3.1686	-2.5900	0.2675
C	1.1381	-1.8960	0.4961
H	-0.7292	-0.8762	1.0707
H	1.6591	3.0881	0.3037
C	-0.2689	4.0475	0.1304
H	-2.3355	4.6400	-0.0563
H	0.7247	-2.8773	0.6880
H	0.1210	5.0559	0.1643
C	-3.7455	-0.3525	-0.7333
H	-4.3579	0.1100	0.0470
H	-3.6821	0.3498	-1.5708
H	-4.2526	-1.2551	-1.0742
C	-2.3143	-2.6497	0.0073
H	-2.3493	-3.0280	-1.0185
H	-1.5730	-3.2362	0.5563
H	-3.2914	-2.8398	0.4566

Free Energy: -3267.814085 hartrees

Intermediate **C3**

Cartesian Coordinates

Pt	-1.8563	-0.6805	-0.0104
N	2.2156	0.6245	0.0122
N	-1.2707	1.4919	0.0022
C	2.9581	-0.4493	0.0033
C	0.8853	0.4487	0.0169
C	0.0590	1.6817	0.0138
C	-2.0917	2.5387	-0.0123
r	4.8561	-0.1814	-0.0098
C	2.4684	-1.7485	-0.0011
C	0.2361	-0.7947	0.0163
C	0.5990	2.9617	0.0148
C	-1.6260	3.8425	-0.0128
H	-3.1507	2.3146	-0.0261
H	3.1403	-2.5946	-0.0126
C	1.0898	-1.8992	0.0072
H	-1.9362	-0.8112	1.4682
H	1.6726	3.0786	0.0225
C	-0.2547	4.0519	0.0010
H	-2.3253	4.6659	-0.0262
H	0.6822	-2.9046	0.0007
H	0.1462	5.0569	-0.0012
C	-3.9188	-0.3377	-0.0359
H	-4.2178	0.4042	0.7105
H	-4.1839	0.0662	-1.0210
H	-4.5193	-1.2332	0.1311
C	-2.1549	-2.6750	-0.0195
H	-1.9749	-3.0096	-1.0467
H	-1.4486	-3.1861	0.6349
H	-3.1664	-2.9553	0.2683

Free Energy: -3267.821263 hartrees

Transition State **CTS3-4**

Imaginary Frequency at -584 cm^{-1}

Cartesian Coordinates

Pt	1.8481	-0.6805	-0.0264
N	-2.2097	0.6300	0.0016
N	1.2794	1.4909	-0.0186
C	-2.9527	-0.4443	0.0116
C	-0.8802	0.4537	0.0016
C	-0.0506	1.6846	-0.0076
C	2.0976	2.5400	-0.0136
Br	-4.8489	-0.1749	0.0095
C	-2.4651	-1.7445	0.0246
C	-0.2354	-0.7910	0.0092
C	-0.5910	2.9635	0.0014
C	1.6324	3.8443	-0.0020
H	3.1576	2.3222	-0.0170
H	-3.1382	-2.5894	0.0343
C	-1.0869	-1.8965	0.0249
H	2.8675	-0.6900	-1.1547
H	-1.6648	3.0791	0.0093
C	0.2618	4.0546	0.0040
H	2.3328	4.6667	0.0030
H	-0.6768	-2.9006	0.0357
H	-0.1392	5.0595	0.0130
C	3.9283	-0.3776	0.2442
H	4.3812	0.4563	-0.2909
H	3.9106	-0.1424	1.3121
H	4.5359	-1.2656	0.0838
C	2.0553	-2.6906	-0.0022
H	1.6237	-3.0638	0.9310
H	1.5092	-3.1451	-0.8310
H	3.0958	-3.0101	-0.0565

Free Energy: -3267.813276 hartrees

Intermediate **C4**

Cartesian Coordinates

Pt	1.0844	-0.4576	-0.2759
N	-2.9194	0.5611	0.0841
N	0.4879	1.6432	-0.1170
C	-3.5745	-0.5688	0.0893
C	-1.5874	0.5011	-0.0257
C	-0.8446	1.7773	-0.0177
C	1.2665	2.7217	-0.1142
C	-2.9813	-1.8205	-0.0073
C	-0.8401	-0.6906	-0.1396
C	-1.4380	3.0277	0.0882
C	0.7471	4.0019	-0.0121
H	2.3303	2.5352	-0.1948
H	-3.5799	-2.7199	0.0082
C	-1.6016	-1.8645	-0.1220
H	-2.5132	3.0966	0.1673
C	-0.6289	4.1521	0.0905
H	1.4098	4.8552	-0.0130
H	-1.1169	-2.8294	-0.1967
H	-1.0672	5.1380	0.1722
C	1.4608	-2.4454	-0.4411
H	1.1815	-2.9859	0.4697
H	0.8983	-2.8901	-1.2671
H	2.5195	-2.6535	-0.6323
O	3.2570	0.1582	-0.3856
S	4.3717	-0.7738	0.0804
C	4.0000	-1.1295	1.7912
H	3.1143	-1.7621	1.7940
H	4.8460	-1.6571	2.2289
H	3.8060	-0.1900	2.3067
C	5.7528	0.3283	0.3231
H	6.0379	0.7005	-0.6580
H	5.4404	1.1443	0.9724
H	6.5726	-0.2376	0.7617
Br	-5.4810	-0.4604	0.2462

Free Energy: -3780.549316 hartrees
